# Health Care Workers’ Need for Headspace: Findings From a Multisite Definitive Randomized Controlled Trial of an Unguided Digital Mindfulness-Based Self-help App to Reduce Healthcare Worker Stress

**DOI:** 10.2196/31744

**Published:** 2022-08-25

**Authors:** Heather Taylor, Kate Cavanagh, Andy P Field, Clara Strauss

**Affiliations:** 1 School of Psychology University of Sussex Brighton United Kingdom; 2 Sussex Partnership NHS Foundation Trust Hove United Kingdom

**Keywords:** self-help, mindfulness, randomized control trial, health care worker, National Health Service, NHS, doctors, nurses, stress, mental health, burnout, mobile phone

## Abstract

**Background:**

Health care workers experience high stress. Accessible, affordable, and effective approaches to reducing stress are lacking. In-person mindfulness-based interventions can reduce health care worker stress but are not widely available or accessible to busy health care workers. Unguided, digital, mindfulness-based self-help (MBSH) interventions show promise and can be flexibly engaged with. However, their effectiveness in reducing health care worker stress has not yet been explored in a definitive trial.

**Objective:**

This study aimed to investigate the effectiveness of an unguided digital MBSH app (Headspace) in reducing health care worker stress.

**Methods:**

This was a definitive superiority randomized controlled trial with 2182 National Health Service staff in England recruited on the web and allocated in a 1:1 ratio to fully automated Headspace (n=1095, 50.18%) or active control (Moodzone; n=1087, 49.82%) for 4.5 months. Outcomes were subscales of the Depression, Anxiety, and Stress (primary outcome) Scale short form; Short Warwick Edinburgh Mental Well-being Scale; Maslach Burnout Inventory; 15-item Five-Facet Mindfulness Questionnaire minus Observe items; Self-Compassion Scale–Short Form; Compassionate Love Scale; Penn State Worry Questionnaire; Brooding subscale of the Ruminative Response Scale; and sickness absence.

**Results:**

Intention-to-treat analyses found that Headspace led to greater reductions in stress over time than Moodzone (*b*=–0.31, 95% CI –0.47 to –0.14; *P*<.001), with small effects. Small effects of Headspace versus Moodzone were found for depression (*b*=–0.24, 95% CI –0.40 to –0.08; *P*=.003), anxiety (*b*=–0.19, 95% CI –0.32 to –0.06; *P*=.004), well-being (*b*=0.14, 95% CI 0.05-0.23; *P*=.002), mindfulness (*b*=0.22, 95% CI 0.09-0.34; *P*=.001), self-compassion (*b*=0.48, 95% CI 0.33-0.64; **P*<*.001), compassion for others (*b*=0.02, 95% CI 0.00-0.04; *P*=.04), and worry (*b*=–0.30, 95% CI –0.51 to –0.09; *P*=.005) but not for burnout (*b*=–0.19, –0.04, and 0.13, all 95% CIs >0; *P*=.65, .67, and .35), ruminative brooding (*b*=–0.06, 95% CI –0.12 to 0.00; *P*=.06), or sickness absence (γ=0.09, 95% CI –0.18 to 0.34). Per-protocol effects of Headspace (454/1095, 41.46%) versus Moodzone (283/1087, 26.03%) over time were found for stress, self-compassion, and compassion for others but not for the other outcomes. Engagement (practice days per week) and improvements in self-compassion during the initial 1.5-month intervention period mediated pre- to postintervention improvements in stress. Improvements in mindfulness, rumination, and worry did not mediate pre- to postintervention improvements in stress. No serious adverse events were reported.

**Conclusions:**

An unguided digital MBSH intervention (Headspace) can reduce health care workers’ stress. Effect sizes were small but could have population-level benefits. Unguided digital MBSH interventions can be part of the solution to reducing health care worker stress alongside potentially costlier but potentially more effective in-person mindfulness-based interventions, nonmindfulness courses, and organizational-level interventions.

**Trial Registration:**

International Standard Randomised Controlled Trial Number ISRCTN15424185; https://tinyurl.com/rv9en5kc

## Introduction

### Background

Even before the COVID-19 pandemic, findings from meta-analyses demonstrated a high prevalence of stress in health care workers worldwide [1-3]. Stress is a vulnerability factor for work-related burnout [4], anxiety, and depression [5], all of which are disproportionately prevalent among health care workers [6-8], and stress also increases the risk of several long-term physical health conditions [9-11]. In the National Health Service (NHS) in England, which employs >1.3 million health care staff [12], 46.8% of staff reported feeling unwell because of work-related stress [12], a figure that has steadily risen since 2016. Almost one-quarter of the days lost to staff sickness in the NHS are because of stress, anxiety, depression, or other mental health problems [13], and similar concerns have been noted in health care systems worldwide [14]. Moreover, stress among health care workers can compromise patient outcomes and safety [15]. The COVID-19 pandemic has further exacerbated stress and distress for health care workers [16,17]; therefore, there is an urgent need to find effective, accessible, and affordable ways of reducing health care workers’ stress.

Mindfulness involves intentionally bringing curiosity and nonjudgmental awareness to present-moment experiences such as thoughts, feelings, and physical sensations as they arise [18,19]. Mindfulness-based interventions (MBIs) typically involve teaching mindfulness in in-person group settings through 8-week courses such as mindfulness-based cognitive therapy (MBCT) [20] and mindfulness-based stress reduction (MBSR) [21], with mindfulness practice and teacher-led discussion of practice being core intervention ingredients. There is substantial evidence from meta-analyses of randomized controlled trials (RCTs) that MBCT reduces the risk of relapse in people with a history of recurrent depression [22] and that MBIs improve symptoms of a range of mental health problems [23]. The degree of engagement in mindfulness practice during MBIs is associated with treatment outcomes [24], and MBI mechanisms of action include mindfulness, rumination, worry, and self-compassion [25].

The benefits of MBIs extend beyond clinical populations, with RCTs demonstrating beneficial effects on stress in nonclinical populations [26], including working adults [27] and, specifically, health care workers [28-30]. However, there are several barriers to health care workers attending in-person MBIs, including the lack of availability [31]; high workplace demands [32,33] that make it difficult for health care workers to find the time to attend; and stigma-related concerns regarding negative social judgments and disclosure and confidentiality, which are more common among health care workers than among those working in other settings [34].

Fortunately, mindfulness-based self-help (MBSH) has the potential to increase opportunities for engagement with MBIs through a plethora of MBSH books, web-based courses, and available smartphone apps. In addition, meta-analyses of RCTs of MBSH have indicated promising effects on stress and mental health outcomes across a range of populations [35,36]. Digital MBSH using smartphone apps has the potential to be particularly accessible as it does not rely on the user having a computer or book on hand to engage with the intervention when needed. Headspace [37] is a smartphone app with >70 million users to date worldwide [38]. There is emerging empirical literature exploring the effectiveness of MBSH apps, including Headspace [39]. Preliminary findings show potential benefits in nonclinical samples, including health care workers; however, the study sample sizes were too small to draw definitive conclusions regarding this working population. Given the early stage of research in this area and studies with small sample sizes, the potential of unguided digital MBSH as a health care–wide solution to reduce health care worker stress is yet to be explored in an adequately powered trial. Although MBSH can effectively reduce stress in a range of nonclinical populations, it is possible that the particularly high demands of working in health care [32,33] will mean that when offered at scale, health care staff may struggle to engage with the intervention, leading to disappointing outcomes. The learnings available from a definitive trial of unguided digital MBSH are particularly important in the current context of rising health care worker stress during the COVID-19 pandemic.

### Objectives

This study sought to overcome some of the methodological limitations of previous related studies and extend our understanding of the potential effects of unguided MBSH among health care workers. The aim of this large multisite RCT was to explore the effectiveness of unguided digital MBSH in comparison with an active control condition (it should be noted that comparisons with active controls are lacking in RCTs of MBIs [29]) for health care workers in targeting stress (primary outcome), mental health outcomes (depression, anxiety, and well-being), work-related outcomes (work-related burnout, sickness absence, and compassion for others), and proposed mechanisms of action (intervention engagement, rumination, worry, mindfulness, and self-compassion). To explore its potential as a health care–wide intervention to reduce health care worker stress, the trial recruited across the full range of NHS organization types (general practitioner or primary care, hospital trusts, community trusts, mental health and/or learning disability trusts, and ambulance trusts), across geographically and sociodemographically diverse regions of England, and across a range of NHS job roles (medical, nursing, allied health professions, and psychological and wider health care support roles). The primary hypothesis was that participants allocated to unguided digital MBSH will show greater reductions in stress from the baseline to postintervention time points (4.5 months following randomization) in comparison with participants in the active control trial arm. The secondary hypotheses were that unguided digital MBSH will be more effective than active control in improving mental health outcomes, work-related outcomes, and potential mechanisms of action from baseline to after the initial intervention period (1.5 months after randomization) and from the baseline to postintervention time points. Analyses examining whether intervention engagement and improvements in mindfulness, self-compassion, worry, and rumination mediated the effects of the intervention on improvements in stress were planned to ascertain intervention-specific mechanisms of action.

## Methods

### Trial Design and Ethics Approval

This study was a 2-arm superiority definitive RCT, with a 1:1 allocation and no stratification, comparing unguided digital MBSH (Headspace [37]) with an active control group (the NHS digital platform for work-related stress, Moodzone [40]). Assessments were performed at 3 time points: baseline (time point 1 [T1]), after the initial intervention period (time point 2 [T2]; 1.5 months after randomization), and at the postintervention time point (time point 3 [T3]; 4.5 months after randomization).

Ethics approval (reference ER/HT207/8) was provided by the University of Sussex, and study approval was granted by the Health Research Authority (reference 16/HRA/5525). The study was prospectively registered on the International Standard Randomised Controlled Trial Number register (reference number: 15424185) [41].

### Participants and Recruitment

Participants had to (1) be employed within an NHS Trust or general practitioner practice in England, (2) be working in roles that involved direct contact with patients for a minimum of 1 day per week, (3) be currently in work (ie, not on long-term sickness absence), (4) be willing to refrain from engaging in other psychological interventions during the course of the study, (5) have regular personal access to an Apple, Android smartphone, or tablet or a computer with internet access, (6) be aged ≥18 years, and (7) have sufficient English language skills to read and understand the intervention materials. There were no additional exclusion criteria. Recruitment took place between February 21, 2017, and September 18, 2018.

Sample size calculations were conducted using G*Power [42], which indicated that 527 complete cases per study arm (1054 total) would be needed to detect a small between-group difference of Cohen *d*=0.20 (*P*=.05; 90% power; 2-tailed) on the primary outcome (stress at T3), with this estimate based on a meta-analysis of MBSH on stress outcomes [36]. A conservative estimate of a 50% study dropout rate was assumed [35], giving a total required sample size of 2108 (n=1054 per arm).

**Figure 1:**
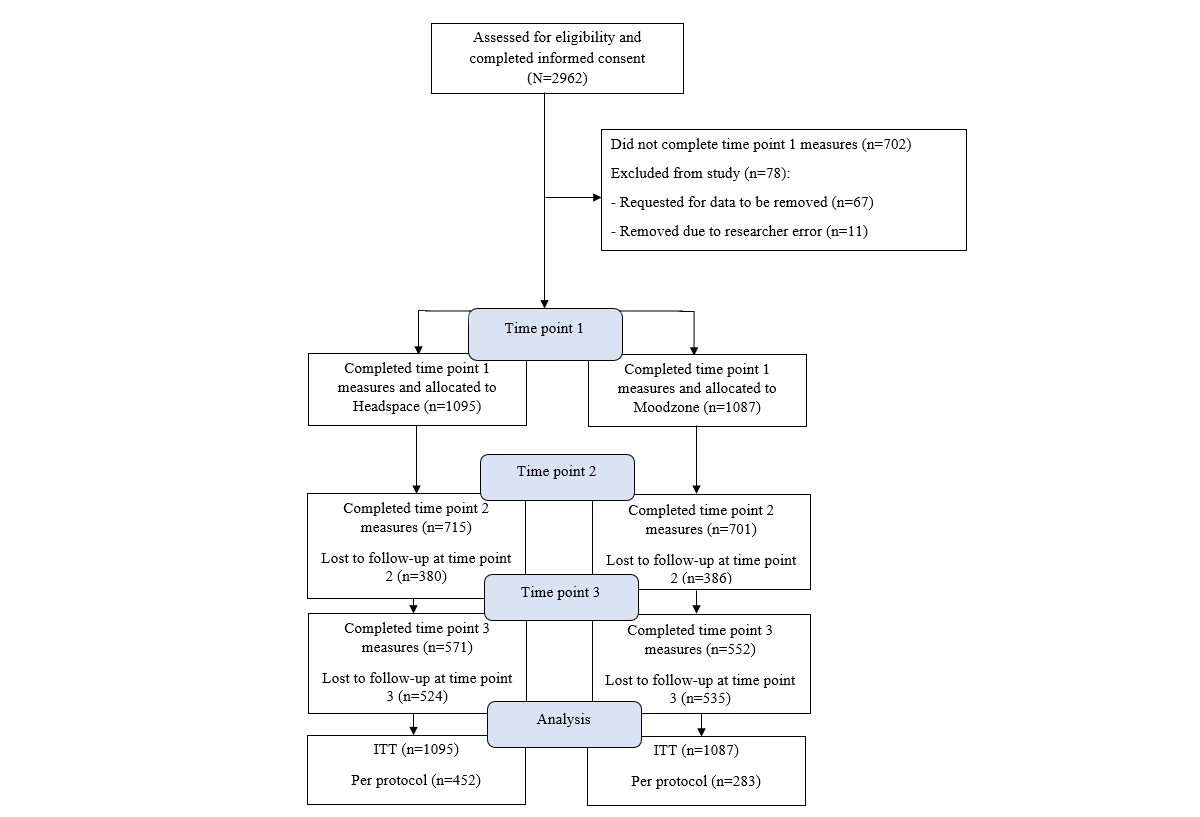
CONSORT (Consolidated Standards of Reporting Trials) diagram showing participant flow. ITT: intention-to-treat.

A total of 2182 participants were enrolled in the study (completed baseline measures and were randomized); 1095 (50.18%) were randomized into the Headspace arm, and 1087 (49.82%) were randomized into the Moodzone arm. The participant flow is shown in the CONSORT (Consolidated Standards of Reporting Trials) diagram (Figure 1), and further participant details are reported in the *Results* section.

### Interventions

#### Headspace

The Headspace MBSH digital program [37] offers a range of brief mindfulness-based practices alongside psychoeducational materials. The Headspace MBSH digital program can be accessed via a website [43] or an app available on the Apple app store or Android Play store. Headspace offers a range of mindfulness-based practices and psychoeducational animations, including an introductory series that comprises daily sessions designed to teach foundational mindfulness principles and practices, as well as packs designed for more specific emotional difficulties (eg, stress and anxiety) and brief *SOS* mindfulness practices designed to be used in times of acute stress. Headspace also offers guidance on informal mindfulness practices that can be undertaken while performing everyday activities, such as running and cycling, and there is written information, including research evidence, related to mindfulness and a frequently asked questions section. At the time of the study, mindfulness practices were verbally guided by Andy Puddicombe, a founder of Headspace with many years of experience in mindfulness practice. For the introductory sessions, users were verbally guided to bring nonjudgmental awareness to the body, breath, thoughts, and feelings, with later sessions also inviting users to bring awareness to difficulties arising during practice (eg, boredom and restlessness) and behavioral choices. At the time of recruitment, users were invited to start the Headspace program by completing the *Take Ten* introductory pack, which involved undertaking guided 10-minute mindfulness practices daily for 10 consecutive days. Upon completion of the *Take Ten*, participants were provided with unlimited access to the full range of Headspace content. Although participants were free to choose the content they engaged with, they were invited to perform at least one 10-minute mindfulness practice daily for the duration of the study. Although practices range in length from 3 to 20 minutes, users can select the duration of most sessions. As Headspace is a *live* product, the program structure was nonstatic, and participants were able to access newly released and changing content as it became available.

#### Moodzone

The NHS Moodzone psychoeducational digital platform [40] was used as an active control. At the time of recruitment, the website offered a range of evidence-based psychosocial recommendations, advice, and guidance on how to manage work-related stress and mental health difficulties effectively. The initial web page was divided into the following sections: “What causes work stress?” “How to manage work stress,” “Learn to speak out,” “Spot the signs of work stress,” and “Who else can help with work stress?”; each provided information and recommendations or guidance relevant to the respective questions. Moodzone also included information, videos, audio tracks, podcasts, and links to other related resources. Participants were invited to engage with the Moodzone website for 10 minutes per day for the duration of the study. It should be noted that although very similar content is still available [44], the Moodzone website used in this study is no longer active. As with Headspace, a *live* nonstatic version of Moodzone was used in the study, meaning that participants could access new and changing content as it became available. Before this study, adequately powered Moodzone trials were not undertaken. However, related evidence from a meta-analysis of RCTs identified a significantly small effect (Cohen *d*=0.20; *P*=.04) of passive psychoeducational interventions compared with control conditions in reducing depression and psychological distress at the postintervention time point [45].

### Measures

Full details of the measures are shown in Multimedia Appendix 1 [4,46-55].

Participants completed the measures described in Textbox 1 at T1, T2, and T3 unless stated otherwise.

**Participant measures.**

**Participant measures**

- Short version of the 21-item Depression, Anxiety, and Stress Scale [46]; the Stress subscale was the primary outcome, with time point 3 (T3) being the primary end point
- Short Warwick Edinburgh Mental Well-being Scale [47]
- Maslach Burnout Inventory [4]
- 15-item version (minus “observe”) of the Five Facets of Mindfulness Questionnaire [48]
- Self-Compassion Scale–Short Form [49]
- Compassionate Love Scale [50]
- Penn State Worry Questionnaire [51]
- Brooding subscale of the Ruminative Response Scale [52]
- Sickness absence measured at time point 1 [T1] and T3 was assessed using 1 item that asked participants to report how many days they had been absent from work because of sickness during the past 3 months
- Demographic information assessed at T1 included participants’ age; gender; marital status; number of children aged <18 years; number of children aged ≥18 years; National Health Service job role; trust and team; number of hours worked per week in the National Health Service job role; highest level of education; individual and household annual incomes; ethnicity; and perceived relative socioeconomic status, with response options from 1 (lowest) to 10 (highest) perceived socioeconomic status [53]
- Intervention expectancy at T1 (Credibility and Expectancy Questionnaire [54])
- Self-reported intervention engagement at time point 2 [T2] and T3:
- Formal engagement: self-reported average number of days per week spent following guided mindfulness meditation on Headspace or following a recommended stress management or well-being strategy on the Moodzone web page
- Informal engagement: self-reported average number of days per week participants brought mindfulness to daily activities or recommended stress management and well-being strategies from Moodzone into their daily lives; at T2, these questions were asked in relation to the past month, and at T3, they were asked in relation to the past 3 months
- Intervention evaluations at T2 and T3: participants asked how likely they were to recommend the intervention to friends and family, how much they really felt that their allocated intervention had helped their well-being, and how likely they were to continue practicing mindfulness (Headspace participants) or stress management and well-being strategies (Moodzone participants) over the following 6 months
- Hypothesis guess at T3: participants asked to state what they thought the purpose of the study was
- Intervention deviations at T3: participants asked to indicate whether they had engaged in the alternative study intervention during the course of the study
- Prior mindfulness experience at T3: participants asked to indicate their experiences of mindfulness before the study, including mindfulness-based cognitive therapy, stress reduction, mindfulness-based self-help, and Headspace, and how often they practiced mindfulness
- Serious adverse events were recorded in accordance with the National Institute for Health Research Good Clinical Practice guidelines [55]
- Participants were also asked to indicate the extent to which they agreed or disagreed that they had experienced “lasting bad effects” from using their allocated intervention (based on Crawford et al [56]); if the participants agreed or strongly agreed, they were asked to provide further details

### Procedure

NHS staff were recruited via posters and leaflets in NHS settings, invitation emails sent through NHS organizations, and study advertisements on staff web pages or newsletters. Potential participants were directed to the study website hosted by Qualtrics XM [57], where they could read the participant information and confirm their eligibility and informed consent (Multimedia Appendix 2). After consenting, the participants were emailed a weblink along with a unique ID code and asked to self-complete the T1 measures on Qualtrics. Participants completed T1 measures, which were allocated automatically to Headspace or Moodzone using a 1:1 block randomization with a block size of 4 by Qualtrics. To ensure allocation concealment, the members of the research team responsible for collecting data and communicating with participants were blinded to the block size. Participants were informed of their random allocation and subsequently asked to indicate their views on the credibility and expectations of their assigned intervention.

Following the completion of the T1 assessment, participants were emailed information on how to access their allocated intervention. Intervention participants were given 12 months of free access to Headspace, and Moodzone was available free of charge. Allowing 5 days for participants to receive this information or download their intervention, participants were invited to engage with their allocated intervention for 10 minutes per day, every day during the initial 30-day study period. At 35 days after randomization, participants were emailed a link to complete the T2 assessments on Qualtrics and invited to continue engaging with their allocated intervention for 10 minutes per day during the remaining 90-day study period. On average, T2 was completed at 1.5 months (SD 0.57) after randomization. At 125 days after randomization, participants were emailed a link to complete the T3 assessment on Qualtrics, with T3 completed at an average of 4.5 months (SD 0.53) from randomization. At this point, the participants who completed the study were given access to the alternative intervention.

Participants who did not complete assessments within 1 week of them being sent were reminded to do so via email. One reminder email was sent for completion of the T1 assessments, and a maximum of 4 reminder emails at weekly intervals were sent for T2 and T3 assessments. The research team was available to answer technical questions or queries via email. No further support was provided.

To improve trial quality and blind participants to the study condition and direction of study hypotheses, advertisements about the study simply referred to both conditions as “online interventions to reduce NHS staff stress,” and details of the alternative or nonallocated intervention were not communicated to participants until T3 assessments (after outcome and engagement measures had been taken). As all assessments were completed on the web without researchers present, the potential for researcher bias to influence assessment outcomes was minimized. All but the mediation analysis was conducted blind to the study arm.

Participants were given the option to enter a prize draw to win 1 of 5 gift vouchers for £50 (US $60).

### Data Analysis Plan

Descriptive statistics are reported by trial arm and time as means and SDs (for continuous data), medians and IQRs (for ordinal data), and counts and percentages (for categorical data). Data analysis was conducted using SPSS (version 25; IBM Corp) [58] and R (version 4.0.2; R Foundation for Statistical Computing) [59] and the following packages: *emmeans* [60], *lme4* [61], *mice* [62], *papaja* [63], and *tidyverse* [64].

#### Handling Missing Data

A minimal number of items were missing at the item level, and missing values for missing items were imputed (using a single imputation) using predictive mean matching in *mice* [65]. At the scale level, multiple imputation was used to handle missing values. Further details are provided in Multimedia Appendix 3 [60-62].

#### Model Selection

As participants were nested within job roles (level 3), there are good reasons for model variations in intervention effects between job roles [66]. There is participant-level randomization to intervention arms in such a model, and job roles act as a crossed effect. We can think of time (*i*) as being nested within participants (*j*), which is nested within job roles (*k*); however, the effect of the treatment arm occurs at level 2 (the participant level), not level 3 (the job role level), of the hierarchy. This situation is described by the model given in Textbox 2.

This saturated model includes random effects for time, trial arm, and their interaction at level 3. However, this model resulted in convergence problems that yielded erratic estimates of random effects involving the trial arm in the raw sample and nearly all imputed samples. On the basis of this preanalysis, a simpler model seemed more appropriate, in which only time was treated as a random effect and only at level 2. However, to model level 3 variability in outcomes, a random intercept (at level 3) was included. This simpler model converged in all the imputed samples. The resulting model is described in Textbox 3 (notice that at level 3, a total of 2 random effects have been knocked out).

To sum up, the hypotheses were tested using a growth model fit as a general linear mixed model, with observations (level 1) nested within participants (level 2) nested within job roles (level 3). Time (time from baseline at which responses were recorded) and trial arm were predictors. The effect of the intervention was quantified and tested with the interaction between time and trial arm, which shows the degree to which the change in the outcome over time is different between the 2 trial arms. Between-group effects were reported separately at T2 and T3 in the event of significant (*P*<.05) trial arm × time interactions. The primary analysis was conducted on the intention-to-treat (ITT) sample with multiple imputed data sets. Secondary analysis was conducted on the per-protocol sample (formal engagement T1-T2 on at least 3 days per week [67]) with the multiple imputed data sets.

Details of the plan for reliable change analysis, mediation analysis, and randomization check can be found in Multimedia Appendix 4 [13,18,25,29,30,36,56,67-72].

**The saturated model showing the data structure.**

**Level 1**

- Depression, Anxiety, and Stress Scale–Stress_ijk_= π_0jk_+ π_1jk_Time_ijk_+ 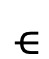
_ijk_

**Level 2**

- π_0jk_= γ_00k_+ γ_01k_Trial arm_jk_+ ζ_0jk_
- π_1jk_= γ_10k_+γ_11k_Trial arm_jk_+ ζ_1jk_

**Level 3**

- γ_00k_= δ_000_+υ_0k_
- γ_10k_= δ_100_+υ_1k_
- γ_01k_= δ_010_+υ_2k_
- γ_11k_= δ_110_+υ_3k_

**The fitted model.**

**Level 1**

- Depression, Anxiety, and Stress Scale–Stress_ijk_= π_0jk_+ π_1j_Time_ijk_+ 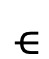
_ijk_

**Level 2**

- π_0jk_= γ_00k_+ γ_01_Trial arm_jk_+ ζ_0jk_
- π_1j_=γ_11_Trial arm_jk_+ ζ_1jk_

**Level 3**

- γ_00k_= δ_000_ + υ_0k_

## Results

### Overview

Table 1 presents the demographic characteristics of the participants by study arm, and Table 2 presents descriptive statistics on all outcome measures at all time points by study arm. Table 1 shows that participants represented a broad range of NHS Trust types and health care professions. As would be expected of a health care workforce, most participants were educated to at least an undergraduate degree level and were earning, on average, the median UK annual salary; most participants were working full-time. Participants covered the full working age spectrum, although they were disproportionately White and female. For the randomization check, all Bayes factors were very close to 0, suggesting very strong evidence for the null hypothesis: randomization was successful in balancing demographic and baseline measurements across the 2 trial arms (Multimedia Appendix 5). There were also no differences in dropout rates between the trial arms. A formal analysis using a multilevel generalized linear model, with a random intercept, predicting dropout (1=in the study and 0=dropped out) from the trial arm, study wave (as a categorical variable), and their interaction showed no significant effects.

**Table 1:** Demographic characteristics of participants (N=2182).

Characteristics		Moodzone	Headspace
**Highest educational achieved, n (%)**			
	GCSE^a^ or NVQ 2^b^ or below (equivalent to not completing high school)	62 (2.84)	69 (3.16)
	A-level or equivalent (equivalent to completing high school)	132 (6.05)	124 (5.68)
	Undergraduate degree	430 (19.71)	474 (21.72)
	Postgraduate degree	462 (21.17)	429 (19.66)
	Other	2 (0.09)	2 (0.09)
**Ethnicity, n (%)**			
	Black	13 (0.6)	12 (0.55)
	White	998 (45.74)	1021 (46.79)
	Asian	50 (2.29)	37 (1.7)
	Mixed or multiple	21 (0.96)	19 (0.87)
	Other	2 (0.09)	4 (0.18)
**Gender, n (%)**			
	Female	906 (41.52)	909 (41.66)
	Male	175 (8.02)	181 (8.3)
	Transgender female	0 (0)	0 (0)
	Transgender male	0 (0)	1 (0.05)
	Nonbinary	0 (0)	0 (0)
	Other	1 (0.05)	1 (0.05)
	Prefer not to say	3 (0.14)	4 (0.18)
Age (years), mean (SD; range)		40.42 (10.92; 19-67)	40.64 (11.02; 18-80)
Perceived socioeconomic status (1-10), mean (SD; range)		5.66 (1.50; 1-10)	5.66 (1.49; 1-10)
**Hours worked per week, n (%)**			
	≤30 hours	261 (11.96)	277 (12.69)
	>30 hours per week	825 (37.81)	819 (37.53)
Individual income (£), median (IQR; average exchange rate at the time of the study was £1=US $1.33)		25,000-30,000 (20,000-25,000 to 35,000-40,000)	25,000-30,000 (20,000-25,000 to 35,000-40,000)
**Marital status, n (%)**			
	Living with partner, married, or civil partnership	800 (36.66)	788 (36.11)
	Single	286 (13.11)	307 (14.07)
**Role, n (%)**			
	Allied Health Professional (eg, speech therapist and occupational therapist)	180 (8.25)	208 (9.53)
	Physician	89 (4.08)	78 (3.57)
	Manager	51 (2.34)	51 (2.34)
	Nurse	284 (13.02)	301 (13.79)
	Psychologist, psychological therapist, or practitioner	93 (4.26)	112 (5.13)
	Wider health care team	216 (9.9)	193 (8.85)
	Other	187 (8.57)	175 (8.02)
**NHS^c^ Trust type, n (%)**			
	Acute (hospital)	334 (15.31)	319 (14.62)
	Ambulance	81 (3.71)	71 (3.25)
	Combined (multiple Trust types within one Trust)	293 (13.43)	288 (13.2)
	Community	66 (3.02)	65 (2.98)
	GP^d^	54 (2.47)	77 (3.53)
	Mental health	245 (11.23)	264 (12.1)

^a^GCSE: General Certificate of Secondary Education.

^b^NVQ 2: National Vocational Qualification level 2.

^c^NHS: National Health Service.

^d^GP: general practitioner.

**Table 2:** Descriptive statistics on all outcome measures at all time points (raw complete case data; N=2182).

Measure and arm		Time point 1 (baseline)				Time point 2 (1.5 months)				Time point 3 (4.5 months)		
		Values, n (%)	Values, mean (SD)	95% CI	Values, n (%)		Values, mean (SD)	95% CI	Values, n (%)		Values, mean (SD)	95% CI
**DASS-21^a^ Stress (primary outcome)**												
	Moodzone	1087 (49.82)	16.24 (7.80)	15.78 to 16.71	701 (32.13)		13.92 (7.65)	13.36 to 14.49	552 (25.29)		14.47 (8.11)	13.79 to 15.15
	Headspace	1095 (50.18)	15.67 (7.40)	15.23 to 16.11	715 (32.77)		12.86 (7.06)	12.34 to 13.38	571 (26.17)		12.39 (7.85)	11.74 to 13.03
**DASS-21 Depression**												
	Moodzone	1087 (49.82)	10.72 (8.26)	10.23 to 11.21	701 (32.13)		9.61 (8.37)	8.99 to 10.23	552 (25.29)		9.58 (8.66)	8.86 to 10.31
	Headspace	1092 (50.05)	10.29 (7.76)	9.83 to 10.75	715 (32.77)		8.34 (7.41)	7.79 to 8.88	571 (26.17)		7.87 (8.03)	7.21 to 8.53
**DASS-21 Anxiety**												
	Moodzone	1087 (49.82)	9.06 (7.43)	8.62 to 9.51	701 (32.13)		7.42 (7.1)	6.90 to 7.95	552 (25.29)		7.45 (7.19)	6.85 to 8.05
	Headspace	1095 (50.18)	8.58 (6.99)	8.16 to 8.99	716 (32.81)		6.47 (6.26)	6.02 to 6.93	571 (26.17)		5.97 (6.49)	5.43 to 6.50
**SWEMWBS^b^ Well-being**												
	Moodzone	1087 (49.82)	21.43 (3.61)	21.22 to 21.65	678 (31.07)		22.43 (4.16)	22.12 to 22.75	525 (24.06)		22.27 (4.44)	21.89 to 22.65
	Headspace	1095 (50.18)	21.57 (3.68)	21.35 to 21.79	704 (32.26)		22.7 (3.99)	22.41 to 23.00	550 (25.21)		23.12 (4.41)	22.76 to 23.49
**Maslach^c^ Emotional Exhaustion**												
	Moodzone	1068 (48.95)	26.2 (11.81)	25.49 to 26.91	678 (31.07)		24.31 (12.06)	23.40 to 25.22	531 (24.34)		24.33 (12.47)	23.26 to 25.39
	Headspace	1080 (49.5)	25.65 (12.08)	24.93 to 26.37	703 (32.22)		23.71 (12.15)	22.81 to 24.61	552 (25.29)		23.27 (12.69)	22.21 to 24.33
**Maslach Depersonalization**												
	Moodzone	1067 (48.9)	5.82 (5.72)	5.47 to 6.16	677 (31.03)		5.64 (5.63)	5.21 to 6.06	530 (24.29)		5.68 (5.84)	5.18 to 6.18
	Headspace	1077 (49.36)	5.75 (5.75)	5.40 to 6.09	701 (32.13)		5.38 (5.48)	4.97 to 5.79	552 (25.29)		5.51 (5.67)	5.03 to 5.98
**Maslach Personal Accomplishment**												
	Moodzone	1065 (48.81)	36.5 (7.02)	36.08 to 36.92	677 (31.03)		37.17 (6.98)	36.64 to 37.70	529 (24.24)		36.4 (7.98)	35.72 to 37.09
	Headspace	1074 (49.22)	36.42 (6.74)	36.01 to 36.82	702 (32.17)		37.2 (7.19)	36.67 to 47.73	551 (25.25)		37.39 (7.4)	36.77 to 38.01
**FFMQ-15^d^ (minus Observe subscale)**												
	Moodzone	1085 (49.73)	38.33 (7.04)	37.91 to 38.74	709 (32.49)		39.8 (7.24)	39.27 to 40.33	551 (25.25)		39.89 (7.48)	39. 27 to 40.52
	Headspace	1092 (50.05)	38.22 (6.7)	37.82 to 38.62	717 (32.86)		40.17 (6.59)	39.69 to40.65	574		40.93 (6.68)	40.38 to 41.47
**SCS-SF^e^ Self-Compassion**												
	Moodzone	1085 (49.73)	34.11 (9.03)	33.58 to 34.65	688 (31.53)		36.28 (9.43)	35.57 to 36.99	544 (26.31)		36.29 (9.29)	35.51 to 37.07
	Headspace	1093 (50.09)	33.86 (8.88)	33.33 to 34.38	710 (32.54)		37.3 (9.3)	36.62 to 37.99	560 (25.66)		38.22 (9.34)	37.44 to 38.99
**PSWQ^f^ Worry**												
	Moodzone	1086 (49.77)	54.2 (14.43)	53.34 to 55.06	677 (31.03)		51.33 (14.65)	50.22 to 52.44	526 (24.11)		51.65 (15.18)	50.35 to 52.95
	Headspace	1095 (50.18)	53.53 (14.44)	52.67 to 54.38	704 (32.26)		50.28 (14.33)	49.22 to 51.34	549 (25.16)		49.37 (14.45)	48.15 to 50.58
**RRS^g^ Rumination (Brooding)**												
	Moodzone	1087 (49.82)	10.69 (3.43)	10.49 to 10.89	677 (31.03)		9.97 (3.51)	9.71 to 10.24	519 (23.79)		9.91 (3.45)	9.61 to 10.20
	Headspace	1096 (50.23)	10.39 (3.35)	10.19 to 10.58	703 (32.22)		9.74 (3.19)	9.50 to 9.98	548 (25.11)		9.45 (3.35)	9.17 to 9.73
**CLS^h^ Compassion for Others**												
	Moodzone	1085 (49.73)	4.77 (1.1)	4.71 to 4.84	675 (30.93)		4.64 (1.15)	4.55 to 4.73	518 (23.74)		4.5 (1.24)	4.29 to 4.61
	Headspace	1094 (50.14)	4.78 (1.09)	4.71 to 4.84	702 (32.17)		4.75 (1.12)	4.67 to 4.84	540 (24.75)		4.69 1.17 ()	4.59 to 4.79
**Sickness absence (days in past month)**												
	Moodzone	1086 (49.77)	2.44 (7.45)	1.99 to 2.88	—^i^		—	—	573 (26.26)		2.04 (6.86)	1.48 to 2.60
	Headspace	1095 (50.18)	2.35 (7.08)	1.93 to 2.77	—		—	—	593 (27.18)		2.23 (7.99)	1.58 to 2.87
**Formal engagement (days/week)**												
	Moodzone	N/A^j^	N/A	N/A	653 (29.93)		2.33 (2.01)	2.17 to 2.48	522 (23.92)		1.35 (1.65)	1.21 to 1.49
	Headspace	N/A	N/A	N/A	679 (31.12)		3.56 (2.26)	3.39 to 3.73	544 (26.31)		2.16 (1.91)	2.00 to 2.32
**Informal engagement** **(days/week)**												
	Moodzone	N/A	N/A	N/A	654 (29.97)		2.2 (2.08)	2.04 to 2.36	520 (23.83)		1.4 (1.77)	1.25 to 1.55
	Headspace	N/A	N/A	N/A	679 (31.12)		2.92 (2.22)	2.75 to 3.09	544 (26.31)		3 (2.18)	2.81 to 3.18
**CEQ^k^ credibility**												
	Moodzone	1080 (49.5)	−0.58^l^ (2.41)	−0.72 to −0.44	—		—	—	—		—	—
	Headspace	1082 (49.59)	0.58^l^ (2.55)	0.43 to 0.73	—		—	—	—		—	—
**Expectancy**												
	Moodzone	1081 (49.54)	−0.40^l^ (2.70)	−0.56 to −0.24	—		—	—	—		—	—
	Headspace	1091 (50)	0.39^l^ (2.80)	0.23 to 0.56	—		—	—	—		—	—

^a^DASS-21: 21-item Depression, Anxiety, and Stress Scale.

^b^SWEMWBS: Short Warwick Edinburgh Mental Well-being Scale.

^c^Maslach Burnout Inventory.

^d^FFMQ15: 15-item Five Facets of Mindfulness Questionnaire.

^e^SCS-SF: Self-Compassion Scale–Short Form.

^f^PSWQ: Penn State Worry Questionnaire.

^g^RRS: Ruminative Response Scale.

^h^CLS: Compassionate Love Scale.

^i^Not available.

^j^N/A: not applicable.

^k^CEQ: Credibility and Expectancy Questionnaire.

^l^Means created from subscale totals of *z* scores [54].

### Primary Outcome (Stress)

#### ITT Analysis

**Figure 2:**
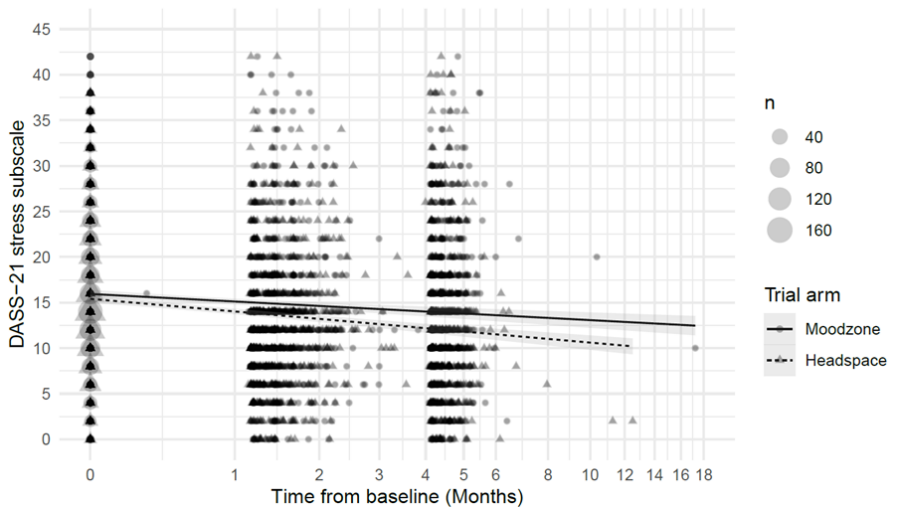
DASS-21 Stress scores over time (intention-to-treat complete case sample). Each triangle represents a Headspace participant, and each circle represents a Moodzone participant. DASS-21: 21-item Depression, Anxiety, and Stress Scale.

Table 3 shows that the main effects of trial arm (Headspace or Moodzone) and time (months) were significant, as was the crucial trial arm × month interaction, which indicates that the trajectories of the 21-item Depression, Anxiety, and Stress Scale (DASS-21) Stress scores over time differed significantly between the 2 trial arms for the ITT sample (Figure 2). The parameter value (*b*=−0.31) tells us that the rate of change (gradient) over time was −0.31 points greater on the DASS-21 Stress subscale per month in the Headspace arm than in the Moodzone arm. Specifically, for every month that passed, DASS-21 stress scores changed by −0.23 units on the scale in the Moodzone group compared with a corresponding change of −0.54 units in the Headspace group (ie, a difference between arms of −0.31 units per month).

To break down this effect, comparisons were made between the estimated marginal means of the outcome from the model at 1.5 (T2) and 4.5 (T3) months in the 2 arms. In the Moodzone arm, stress was significantly higher at baseline than at both 1.5 months (*b*=0.34, SE 0.09; *P*<.001) and 4.5 months (*b=*1.03, SE 0.26; *P*<.001). Stress was also significantly higher at 1.5 months than at 4.5 months (*b=*0.69, SE 0.18; *P*<.001). Similarly, in the Headspace arm, stress was significantly higher at baseline than at both 1.5 months (*b*=0.81; SE 0.08; *P*<.001) and 4.5 months (*b*=2.42, SE 0.25; *P*<.001), and significantly higher at 1.5 months than at 4.5 months (*b*=1.61, SE 0.17; *P*<.001). The *b* values represent the difference in the estimated marginal means; they show that, for example, at 4.5 months, the decrease in DASS-21 Stress compared with baseline was 1.03 points in the Moodzone arm and 2.42 points in the Headspace arm. In other words, at 4.5 months after randomization, Moodzone reduced DASS-21 Stress scores by approximately 1 point along the 42-point scale, and the equivalent change for Headspace was a reduction of approximately 2.5 points along the scale. In addition, the difference in estimated marginal means between the 2 arms was *b*=0.62 (SE 0.31; *P*=.045) at baseline, *b*=1.08 (SE 0.30; *P*<.001) at 1.5 months, and *b*=2.00 (SE 0.42; *P*<.001) at 4.5 months (the preregistered primary end point).

**Table 3:** Model for the 21-item Depression, Anxiety, and Stress Scale Stress (intention-to-treat sample with multiple imputation).

Effect	Unstandardized *b* (SE; 95% CI)	*t* test (*df*)	*P* value
Intercept	15.33 (0.40; 14.55 to 16.11)	38.41 (6054.49)	<.001
Trial arm	−0.62 (0.31; –1.23 to –0.01)	−2.01 (5129.90)	.045
Months	−0.23 (0.06; –0.35 to –0.11)	−3.92 (165.07)	<.001
Trial arm × month	−0.31 (0.08; –0.47 to –0.14)	−3.64 (151.13)	<.001

#### Per-Protocol Analysis

The per-protocol sample included only participants who formally engaged with their allocated intervention at least 3 days per week during the initial intervention period (T1-T2). Multimedia Appendix 6 shows a significant trial arm × month interaction, indicating that the trajectories of stress over time differed significantly between the 2 trial arms. The rate of change over time was −0.28 DASS-21 Stress units greater per month in the Headspace arm than in the Moodzone arm. Specifically, in the Moodzone arm, the rate of change over time was −0.42, which means that for every month that passed, DASS-21 Stress scores decreased by 0.42 points; however, in the Headspace, arm the rate of change over time was −0.70 (a difference of −0.28 between arms), which means that for every month that passed, DASS-21 Stress decreased by 0.70 points.

In the per-protocol sample in the Moodzone arm, stress was significantly higher at baseline than at both 1.5 months (T2; *b*=0.63, SE 0.16; *P*<.001) and 4.5 months (T3; *b*=1.88, SE 0.47; *P*<.001). Stress was also significantly higher at 1.5 months than at 4.5 months (*b*=1.26, SE 0.31; *P*<.001). Similarly, in the Headspace arm, stress was significantly higher at baseline than at both 1.5 months (*b*=1.05, SE 0.12; *P*<.001) and 4.5 months (*b*=3.14, SE 0.35; *P*<.001) and significantly higher at 1.5 months than at 4.5 months (*b*=2.09, SE 0.24; *P*<.001). The *b* values represent the difference in the estimated marginal means; they show that, for example, at 4.5 months the decrease in stress compared with baseline was 1.88 points in the Moodzone arm and 3.14 points in the Headspace arm. In addition, the difference in estimated marginal means between the 2 arms was not significant at baseline (*b*=0.24, SE 0.52; *P*=.65) or at 1.5 months (*b*=0.66, SE 0.48; *P*=.17) but was significant at 4.5 months (*b*=1.50, SE 0.62; *P*=.02).

#### Reliable Change

Multimedia Appendix 7 provides tables for the reliable change analysis. Overall, 20.5% (71/347) of Moodzone and 29.7% (102/343) of Headspace participants who scored at least in the mild stress range at T1 showed reliable improvement from T1 to T2 in stress, with 2.9% (10/347) and 2% (7/343) showing reliable deterioration, respectively. From T1 toT3, approximately 24.1% (66/247) of Moodzone and 36.8% (100/272) of Headspace participants scored at least in the mild stress range at T1 and showed reliable improvement in stress, with 2.9% (8/274) and 4% (11/272) showing reliable deterioration. The trial arm significantly predicted reliable improvement (compared with no change) at both T2 and T3. At T2, the odds of being classified as having reliable improvement were 1.45 higher in the Headspace than in the Moodzone arm, and 95% CIs did not cross 1 (95% CI 1.05-2.01). At T3, the odds of being classified as having reliable improvement were 1.48 higher in the Headspace than in the Moodzone arm, with 95% CIs not crossing 1 (95% CI1.09-2.02). The odds of being classified as showing reliable deterioration in stress were not different between arms at either T2 (odds ratio 0.71, 95% CI 0.29-1.73) or T3 (odds ratio 1.26, 95% CI 0.55- 2.92) as 95% CIs crossed 1.

### Secondary Outcomes and Additional Analyses

The findings from the ITT analysis of secondary outcomes are shown in Table 4. Further details on the secondary outcomes and additional analyses are provided in Multimedia Appendix 4 [13,18,25,29,30,36,56,67-72] and details of the analysis of lasting negative effects are provided in Multimedia Appendix 8.

**Table 4:** Overall trial arm × time effects of the intervention on all outcomes for the intention-to-treat sample with multiple imputation (Moodzone N=1087 and Headspace N=1095 on the primary outcome)^a^.

Measure	Difference between arms per month, unstandardized *b* (SE; 95% CI)	*t* test (*df*)	*P* value	Differences between arms at 1.5 months			Differences between arms at 4.5 months		
				Unstandardized^b^ *b* (SE)	*P* value	Hedges *g*^c^	Unstandardized^b^ *b* (SE)	*P* value	Hedges *g*^c^
DASS-21^d^ Stress	−0.31 (0.08; −0.47 to −0.14)	−3.64 (151.13)	<.001	1.08 (0.30)	<.001	0.14	2.00 (0.42)	<.001	0.26
DASS-21 Anxiety	−0.19 (0.07; −0.32 to −0.06)	−2.94 (218.51)	.004	0.78 (0.27)	.04	0.14	1.36 (0.34)	<.001	0.22
DASS-21 Depression	−0.24 (0.08; −0.40 to −0.08)	−3.02 (211.23)	.003	0.92 (0.32)	.005	0.16	1.65 (0.43)	.001	0.20
SWEMWBS^e^ Well-being	0.14 (0.04; 0.05 to 0.23)	3.16 (289.19)	.002	−0.35 (0.15)	.02	0.07	−0.77 (0.21)	<.001	0.19
Maslach^f^ Emotional Exhaustion	−0.19 (0.10; −0.39 to 0.01)	−1.85 (372.00)	.07	N/A^g^	N/A	0.05	N/A	N/A	0.08
Maslach Depersonalization	−0.04 (0.05; −0.14 to 0.05)	−0.94 (321.54)	.35	N/A	N/A	0.05	N/A	N/A	0.03
Maslach Personal Accomplishment	0.13 (0.07; −0.01 to 0.27)	1.84 (251.87)	.07	N/A	N/A	0.00	N/A	N/A	0.13
FFMQ-15^h^ (minus Observe)	0.22 (0.06; 0.09 to 0.34)	3.38 (298.64)	.001	−0.31 (0.28)	.26	0.05	−0.96 (0.35)	.006	0.15
SCS-SF^i^ Self-Compassion	0.48 (0.08; 0.33 to 0.64)	6.05 (201.36)	<.001	−0.76 (0.37)	.04	0.11	−2.21 (0.46)	<.001	0.21
CLS^j^ Compassion for Others	0.02 (0.01; 0.00 to 0.04)	2.07 (144.19)	.04	−0.03 (0.05)	.48	0.10	−0.09 (0.06)	.12	0.16
PSWQ^k^ Worry	−0.30 (0.11; −0.51 to −0.09)	−2.83 (278.67)	.005	1.15 (0.59)	.05	0.07	2.06 (0.69)	.003	0.15
RRS^l^ Rumination (Brooding)	−0.06 (0.03; −0.12 to 0.00)	−1.91 (349.8)	.06	N/A	N/A	0.07	N/A	N/A	0.14

^a^A negative value for *b* is in favor of Headspace for the DASS-21 subscales, RRS Brooding, and PSWQ Worry; a positive value for *b* is in favor of Headspace for the SWEMWBS, FFMQ-15 (minus Observe), SCS-SF Self-Compassion, and CLS Compassion for Others.

^b^Unstandardized effects at 1.5 and 4.5 months were only reported in the event of a significant trial arm × time interaction.

^c^Hedges g is the difference between trial arms at time point 2 and time point 3 based on raw data.

^d^DASS-21: 21-item Depression, Anxiety, and Stress Scale.

^e^SWEMWBS: Short Warwick Edinburgh Mental Well-being Scale.

^f^Maslach Burnout Inventory.

^g^N/A: not applicable.

^h^FFMQ15: 15-item Five Facets of Mindfulness Questionnaire.

^i^SCS-SF: Self-Compassion Scale–Short Form.

^j^CLS: Compassionate Love Scale.

^k^PSWQ: Penn State Worry Questionnaire.

^l^RRS: Ruminative Response Scale.

#### Intervention Engagement

Multimedia Appendix 9 shows the self-reported engagement with each intervention. Time was treated categorically (1.5 vs 4.5 months). The model was fitted is as follows:

Level 1: DASS-21*_ij_*=π_0_*_j_* + π1 × Time*_ij_* + 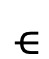
*_ij_*

Level 2: π_0_*_j_*=γ00 + γ01 × Trial arm*_j_* + ζ_0_*_j_*

In the ITT sample, Headspace participants engaged with their allocated intervention formally and informally on more days per week than Moodzone participants, both between T1 and T2 (*b*=−1.32, SE 0.11; *P*<.001 and *b*=−0.79, SE 0.11; *P*<.001, respectively) and between T2 and T3 (*b*=−0.70, SE 0.10; *P*<.001 and *b*=−1.55, SE 0.12; *P*<.001).

#### Mediation Analysis

Formal engagement (practice days per week) from T1 to T2 mediated the effect of trial arm on T1 to T3 improvements in stress using complete case data within the per-protocol sample (582/2182, 26.67%) as 95% CIs did not cross 0 (95% CI −0.097 to −0.006). Similarly, improvement in self-compassion at T1 and T2 significantly mediated T1 to T3 improvement in stress for per-protocol participants (95% CI −0.144 to −0.022). However, improvements in mindfulness, worry, and rumination (brooding) at T1 and T2 did not significantly mediate improvement in stress from T1 to T3 for per-protocol participants, as all 95% CIs crossed 0 (mindfulness: 95% CI −0.107 to 0.029; worry: 95% CI −0.069 to 0.025; brooding: 95% CI −0.046 to 0.037). Overall, the mediation analysis findings suggest that the greater improvement in stress in the Headspace arm in comparison with the Moodzone arm was driven, at least in part, by engagement on more days per week in formal practices and exercises and greater improvement in self-compassion (but not in mindfulness, worry, or rumination) in the Headspace arm during the initial intervention period.

### Intervention Credibility and Expectancy

At T1, between-group differences in intervention credibility and expectancy were assessed via standardized totals of the first and last 3 items of the Credibility and Expectancy Questionnaire, respectively. Headspace was rated as significantly more credible than Moodzone (t_2164.81_=−10.88; *P*<.001; Cohen *d*=0.47). Significantly more positive expectancy ratings were also observed for Headspace compared with Moodzone (t_2170_=−6.70; *P*<.001; Cohen *d*=0.29).

### Awareness of Study Purpose

At T3, only 0.68% (8/1171) of the participants indicated a clear awareness of the study hypothesis. Most of these participants (7/1171, 0.59%) were allocated to Moodzone. The analysis was not conducted between the arms, given the small numbers involved.

## Discussion

### Principal Findings

In this study, we examined whether an unguided digital MBSH intervention (Headspace) was effective in reducing health care worker stress when compared with an active control condition (Moodzone) that was matched for duration and medium (ie, digitally delivered). In contrast to previous studies, this was a fully powered, multisite definitive RCT with patient-facing NHS staff working in a broad range of health care roles and across a broad range of health care organization types, allowing definitive conclusions to be drawn and findings to be generalized.

### Primary Outcome

The stress in both arms improved over time. In comparison with Moodzone, Headspace participants showed a significantly greater reduction in stress (the preregistered primary outcome) over the 4.5-month course of the study, with significant but small differences between trial arms at 1.5 and 4.5 months (the primary endpoint). Headspace participants showed an average reduction in stress over the study period of almost 2.5 points on the 42-point scale, which was over twice the improvement in stress experienced by Moodzone participants. Compared with Moodzone participants, Headspace participants were significantly more likely to experience reliable improvements in stress, both from T1 to T2 and T1 to T3.

The between-group effect on stress at the preregistered primary end point was small (Hedges *g*=0.26), consistent with relevant evidence from 2 recent meta-analyses. For example, Spijkerman et al [36] identified significantly lower levels of stress for unsupported web-based mindfulness and acceptance-based self-help interventions than for control conditions at the postintervention time point among nonclinical samples, with a small effect (Hedges *g*=0.19), whereas a more recent systematic review and meta-analysis conducted by the study team [73] observed a similarly small and statistically significant between-group postintervention effect on stress when unguided MBSH was compared with active control conditions among nonclinical samples (mirroring the design of this study; Hedges *g*=0.20). As such, the modest reductions in stress observed in this study appear to be in keeping with the effects observed for unguided MBSH in the broader literature, and taken together, these observed effects suggest that a small and specific benefit may be associated with such interventions.

Medium to large between-group effects on stress have been reported for the well-established MBSR course in comparison with active and inactive control conditions (Hedges *g*=0.77) [29] and for a newly developed version of MBCT for the workplace, MBCT for Life (MBCT-L), in comparison with wait-list (Cohen *d*=0.72) [30]. Although it is not possible to directly compare with this study because of differences in control conditions, it is likely that these in-person, guided, and more intensive courses are more effective than unguided MBSH. However, there are several barriers to extending the reach of these courses. First, there are not enough mindfulness teachers working in the NHS to offer MBIs to patients in line with the National Institute of Health and Clinical Excellence guidelines [74], let alone to offer MBSR or MBCT-L courses to NHS staff. Second, stigma-related concerns among health care workers about accessing mental health support [34] may hinder uptake, even if in-person MBIs are available. Third, many health care workers struggle to commit to the highly structured and time-intensive nature of traditional MBIs [32,33].

Our study also extends the findings of meta-analyses of RCTs exploring the effects of digital interventions for stress management in the workplace more broadly. When considering smartphone apps specifically, a recent RCT of an unguided non-MBI workplace stress management app based on the Job Demands-Resources Model [75] in comparison with a wait-list found a similarly small effect on stress 6 weeks after randomization (Cohen *d*=0.14) [76]. When considering digital resources more broadly, Heber et al [77] examined the effects of web- and computer-based interventions based on cognitive behavioral therapy (CBT), third-wave CBT (eg, mindfulness and acceptance and commitment therapy), and non–CBT-based interventions (eg, present control interventions and career identity training for stress management) compared with control conditions among nonclinical populations experiencing stress and found a significant between-group postintervention reduction in stress when looking at unguided interventions, with a small effect (Cohen *d*=0.33). In addition, Carolan et al [78] identified significant between-group postintervention improvements in psychological well-being (which included measures of stress), with a small effect (Hedges *g*=0.37), when comparing mainly CBT-based web-delivered interventions with control conditions in the workplace. However, many of the studies considered in these reviews used wait-list control conditions and included guided interventions, which is likely to have contributed to the magnitude of the observed effects.

Unguided digital MBSH interventions, such as Headspace, offer the potential to provide mindfulness training to NHS workers at a scale without the need for a trained mindfulness teacher on site, thus enabling workers to engage with an MBI at a time, place, and pace that suits them. However, to optimize the benefits available from such interventions, it is important that they are offered in a supportive workplace context; are aligned with organizational values, goals, and practices; and protected time and space are available for such self-care [79].

We do not contend that MBSH could or should replace in-person MBIs for NHS workers, given the likely larger effect of in-person courses; however, unguided MBSH interventions could be part of a solution to widening access to mindfulness training while simultaneously endeavoring to find ways of increasing the availability of in-person MBIs. Additional costs associated with providing trained practitioners also put unguided MBSH at an advantage over guided MBSH interventions, as they have the potential to be made more widely available. However, a disadvantage is that effectiveness similarly appears to be reduced, with Spijkerman et al [36] finding significantly smaller between-group effects for mindfulness- and acceptance-based self-help interventions that were unguided (Hedges *g*=0.19) compared with guided interventions (Hedges *g*=0.89). Therefore, what is gained in the widening reach may be lost in reducing the benefits. However, there is emerging evidence that book-based unguided MBSH may produce larger effect sizes than digital MBSH and a direct head-to-head comparison of MBSH formats (especially book vs digital) is warranted [73].

### Intervention Engagement

In comparison with Moodzone, Headspace participants reported a significantly greater number of days spent formally engaging with mindfulness practice. Self-reported practice engagement in the Headspace arm averaged 3.5 days per week during the initial intervention period and 2 days per week during the follow-up period. As such, our findings suggest that sustained commitment to even brief mindfulness practice is challenging for many health care workers; therefore, the reduced practice times afforded by MBSH may provide a more viable alternative to mindfulness training. Interestingly, although daily practice at home is encouraged in MBCT or MBSR, it appears that greater benefits for mental health are seen when people practice at least 3 days a week during the initial intervention period, as compared with people who practice <3 days a week [67]. In this study, 66.6% (452/679) and 37.9% (206/544) of Headspace participants said that they practiced at least 3 days a week at T2 and T3, respectively.

Per-protocol analyses were also conducted to examine the effects of Headspace compared with Moodzone for only those participants who reported formally engaging with their allocated intervention ≥3 days per week during the initial intervention period (based on Crane et al [67]). This shows the overall beneficial effects of Headspace over time in comparison with Moodzone. However, although there were significant between-group effects at T3 in favor of Headspace, between-group effects at T2 were no longer significant. Moreover, most effects of secondary outcomes over time were nonsignificant in the per-protocol analysis. If Headspace engagement is the active ingredient of change, per-protocol effects might be expected to be larger than ITT effects and remain statistically significant, despite the relatively smaller sample contributing to the per-protocol analysis. Therefore, further research is needed to explore the relationship between engagement with Headspace and the magnitude of outcomes.

Given that formal engagement with Headspace (days per week) was greater than that with Moodzone, it could be that once the formal engagement is accounted for in the per-protocol sample (ie, all included participants formally engaged for at least 3 days per week during the initial intervention period), the relative benefits of Headspace over Moodzone are somewhat diminished. However, finding ways of encouraging engagement in unguided digital well-being interventions is a well-recognized challenge [80], and greater engagement with Headspace in comparison with an NHS-developed digital well-being offer is important in itself, as, in the real world, it is the ITT benefits that are realized rather than the per-protocol effects.

Multimedia Appendix 4 [13,18,25,29,30,36,56,67-72] provides a discussion of the findings on secondary outcomes and additional analyses.

### Strengths and Limitations

Although the adequately powered sample size and rigorous study design represent the key strengths of our study, the findings should be considered within the context of several limitations. In this trial, the NHS’s digital workplace stress resource, Moodzone, was selected as the active control condition, inviting study participants to engage with a range of evidence-based recommendations for a minimum of 10 minutes each day as a time match to the Headspace intervention.

However, as previously discussed, intervention engagement was significantly greater for Headspace than for Moodzone; therefore, it is plausible that the active ingredient was intervention engagement rather than intervention content. However, even if Headspace is more effective than Moodzone simply because it is more engaging, this will have implications for real-world effectiveness. To determine the effectiveness of intervention content specifically, future research should compare Headspace with an equally engaging active control. In addition, after providing participants with postrandomization information about their allocated intervention, Headspace received significantly higher credibility and expectancy ratings than Moodzone. Expectancy effects can affect psychotherapeutic outcomes [81], and it is plausible that the greater credibility and expectancy of Headspace than that of Moodzone could explain the study findings. However, the beneficial effects of Headspace on stress outcomes in comparison with Moodzone were retained in a post hoc analysis where credibility and expectancy ratings were entered as covariates, suggesting that the intervention effects cannot be purely explained by the greater credibility and expectancy of Headspace. Future studies should consider the role of credibility and expectancy in more depth and compare Headspace with an intervention matched for credibility and expectancy.

Models were fitted for 11 secondary outcomes, each with 3 predictors (trial arm, time, and their interaction), yielding 33 *P* values. To control for the type I error rate across these models, the reported *P* values for the interaction effects for secondary outcomes in Table 4 were evaluated against a critical *P* value of .002 (ie, .05/33). When evaluating against this stricter criterion, all the interaction effects for secondary outcomes were nonsignificant, except for self-compassion. However, the main goal of *P* value correction is to mitigate fishing expeditions, and all models were preplanned; in addition, the trade-off in controlling type I errors is losing control of type II errors, and there is no inherent reason why controlling type I errors is more desirable. In addition, where the interaction is significant, we tried to carefully evaluate the raw effect size, which adds to the important context of the real-world importance of the effect irrespective of the *P* value.

Recent attention has been paid to the concept of a “digital placebo effect,” whereby nontherapeutic elements of digital interventions are thought to engender either real or imagined improvements in mental health outcomes [82]. As such, it is perhaps also of note that although Headspace was delivered via a sophisticated smartphone app that offered structured daily guidance, Moodzone was delivered via a series of web pages that participants were expected to navigate independently. Therefore, it is possible that the observed effects are, at least in part, because of participants’ more favorable expectations of Headspace relative to Moodzone because of differences in content delivery. Future research should compare Headspace with an active control matched for delivery format and style.

For reasons beyond our control, Headspace was temporarily advertised on the Moodzone web page (notwithstanding the widespread advertising of Headspace on social media and other platforms), which may explain why, despite apparently successful blinding of the study hypotheses, a proportion of Moodzone participants completing measures at T3 reported using Headspace during the study period. However, this is only likely to have diluted between-group differences, and, at worst, our findings can be considered to reflect a conservative estimate of the difference between groups. Moreover, although minor design, platform, and content changes are unlikely to have affected our results [83], it is also worth noting that both Headspace and Moodzone were examined as *live* resources, and as such, both were subject to changes during the study period.

Our study suggests the benefits of an invitation for brief mindfulness-based practices using unguided digital MBSH; however, a *class effect* (ie, the translation of these benefits to any unguided digital MBSH resource) cannot be assumed. Further research is required to identify and optimize the active ingredients of unguided MBSH.

Further limitations of this study are that all outcomes and measures of engagement were self-reported and that dropout at T3 was relatively high, although not atypical for RCTs of digital interventions. Finally, although we recruited a large sample of health care staff working in a variety of job roles and across a variety of NHS organization types across England, our sample was not entirely representative of the NHS workforce. For example, 83.22% (1815/2181) of participants identified as female compared with 77% of NHS staff more broadly [84], and our sample underrepresented Black, Asian, and minority ethnic staff, with 92.74% (2019/2177) White participants in comparison with 77.9% in the NHS workforce [85]. Future studies could monitor demographic characteristics as recruitment progresses and adjust recruitment strategies accordingly to target underrepresented groups.

### Future Research

Future research should match unguided digital MBSH to equally credible active control conditions with equal expectations of benefits. Doing so would help enable greater confidence in conclusions about the relative benefits of mindfulness-based content. Moreover, dismantling trials would also be beneficial to unpick the active ingredients of digital resources such as Headspace.

Another important avenue for future research involves identifying the moderators of engagement. Identifying moderators of engagement with unguided digital MBSH interventions may facilitate the targeted intervention of barriers to and facilitators of regular mindfulness practice to promote engagement and, in turn, potentially boost the effects.

Guided mindfulness- and acceptance-based self-help has larger effects on stress outcomes than unguided approaches [36]. There is a balance to be struck between providing MBSH at scale to more health care workers (without guidance and its associated costs) and providing maximally effective MBSH to potentially fewer health care workers (with guidance). Few head-to-head trials exist, and a well-designed study comparing the clinical effectiveness and cost-effectiveness of guided digital MBSH with unguided digital MBSH for health care workers is warranted to explore the relative advantages and disadvantages of each approach. Future research could also explore the clinical effectiveness and cost-effectiveness of different methods of providing MBSH support and guidance at different levels of intensity (eg, automated but personalized, regular email or text guidance; an MBSH support helpline; asynchronous email support from a trained practitioner; and weekly support sessions with a mindfulness teacher). For interventions that incur a cost to the individual or organization, it is particularly important to have a good understanding of the balance between economic costs (eg, funding a subscription for health care staff in an organization) and economic benefits (eg, sickness absence). Future research should include a full health economic evaluation to examine not only the clinical effectiveness of different MBSH interventions but also their cost-effectiveness. In addition, future research should also examine naturalistic, real-world outcomes of Headspace in specific populations to complement RCT findings.

### Implementation

Overall, the findings suggest that an unguided digital MBSH program appears to be a safe intervention for health care workers, which can yield small but significant improvements in stress and other mental health outcomes with minimal time investment from users. However, it is important to consider that a wide range of non-MBI digital interventions is effective in improving stress and mental health both within [78] and outside the workplace [77] and may be preferred by some health care workers. Furthermore, our findings should be considered within the context of significantly larger effects on stress (in various populations) in guided versus unguided mindfulness- and acceptance-based self-help interventions [36] and larger effects on health care worker stress with MBSR [29] and MBCT-L [30], although this does not directly compare like for like. Although unguided digital MBSH interventions can offer a potential solution to some of the barriers associated with accessing guided MBSH and MBSR or MBCT-L, the smaller effects indicate that a careful balance needs to be struck between effectiveness and accessibility.

It is also worth considering that Headspace was not beneficial for the workplace outcomes of burnout and sickness absence, and as such, alternative strategies will be needed to identify appropriate solutions to these problems. Given the greater effects of MBSR and MBCT-L on health care workers, unguided digital MBSH could also be considered as the first MBI step, with some users moving on to more intensive, as well as more effective, in-person courses. However, this does not dismiss the potential of unguided MBSH, given its scalability. We found that 36.8% (100/272) of Headspace participants showed a reliable improvement in stress over the course of the study compared with 24.1% (66/274) in the Moodzone arm (the NHS digital well-being offer at the time of recruitment). If this difference in reliable improvement were replicated across, for example, 10% of the 1.2 million NHS workforce, this would translate into >15,000 NHS workers showing a reliable improvement in stress if offered Headspace rather than Moodzone.

### Conclusions

Unguided use of a digital MBSH intervention appears safe and is effective in reducing stress in health care workers compared with an active control condition, with improvements in self-compassion and formal intervention engagement explaining, at least in part, its beneficial effects. Effect sizes were small in comparison with in-person MBIs; however, unguided digital MBSH has the potential to be offered as part of a package of approaches to support health care workers’ stress, mental health, and well-being. The findings support offering unguided MBSH as an addition to the ecosystem of evidence-based approaches to support health care workers’ well-being, which offers choices and solutions at different levels of intensity and with different levels of guidance. Unguided MBSH must be contextualized within a supportive environment that promotes self-care at work [79]. Prioritizing the well-being and mental health of health care workers is critical, now more than ever, as we seek to find ways of supporting health care workers to live with the projected aftereffects of the COVID-19 pandemic.

## Appendix

Multimedia Appendix 1 Measures.

Multimedia Appendix 2 Participant information, consent, and debriefing.

Multimedia Appendix 3 Handling missing data.

Multimedia Appendix 4 Additional data analysis plan, results, and discussion.

Multimedia Appendix 5 Bayes factors for assessing randomization success.

Multimedia Appendix 6 Per-protocol findings.

Multimedia Appendix 7 Reliable change tables.

Multimedia Appendix 8 Lasting negative effects.

Multimedia Appendix 9 Self-reported formal and informal intervention engagement.

Multimedia Appendix 10 CONSORT-eHEALTH checklist (V 1.6.1).

## Abbreviations

CBT: cognitive behavioral therapy
CONSORT: Consolidated Standards of Reporting Trials
DASS-21: 21-item Depression, Anxiety, and Stress Scale
ITT: intention-to-treat
MBCT: mindfulness-based cognitive therapy
MBCT-L: mindfulness-based cognitive therapy for Life
MBI: mindfulness-based intervention
MBSH: mindfulness-based self-help
MBSR: mindfulness-based stress reduction
NHS: National Health Service
RCT: randomized controlled trial
T1: time point 1
T2: time point 2
T3: time point 3
