# Supplementary material for: Health Care Workers’ Need for Headspace: Findings From a Multisite Definitive Randomized Controlled Trial of an Unguided Digital Mindfulness-Based Self-help App to Reduce Healthcare Worker Stress
Source: JMIR Mhealth Uhealth. 2022 Aug 25;10(8):e31744. doi: 10.2196/31744 (PMC9459942; doi:10.2196/31744)
Supplement: Multimedia Appendix 1 [file mhealth_v10i8e31744_app1.docx]

# Appendix 1: Measures

Participants completed the measures below at Time 1 (T1), Time 2 (T2) and Time 3 (T3) unless stated otherwise. The Checklist for Reporting Results of Internet E-Surveys (CHERRIES) was adhered to, and the majority of measures (including the primary outcome) were validated for online delivery.

The primary outcome was stress measured using the stress subscale of the 21-item Depression, Anxiety and Stress Scale (DASS-21; [1]. The DASS-21 measures depression, anxiety, and stress via three 7-item subscales. Respondents are asked to indicate how much each of the negatively phrased statements applied to them over the previous week on a 4-point Likert-type scale, with response options of: 0 (“Never”) to 3 (“Almost always”). Example items for stress include “I found it difficult to wind down” and “I tended to over-react to situations”. Totals for each subscale are calculated by multiplying summed scores by two, with each subscale therefore yielding a potential total range of 0 to 42-points, with higher scores indicating higher levels of depression, anxiety, or stress. Henry and Crawford [2] found the DASS-21 subscales to validly measure each construct, while Antony et al [3] judged the internal consistency and concurrent validity of the measure to be in the acceptable to excellent ranges. The stress subscale demonstrated good reliability (α = .84) in our sample at T1.

Secondary outcomes and mechanism of action variables were:

- Anxiety (DASS-21 Anxiety subscale): Anxiety was measured using the anxiety subscale of the DASS-21. Example items include “I was aware of dryness of my mouth” and “I was worried about situations in which I might panic and make a fool of myself”. The anxiety subscale demonstrated good reliability (α = .81) in our sample at T1.
- Depression (DASS-21 Depression subscale): Depression was measured using the depression subscale of the DASS-21. Example items include “I couldn’t seem to experience any positive feeling at all” and “I found it difficult to work up the initiative to do things”. The depression subscale demonstrated good reliability (α = .89) in our sample at T1.
- Wellbeing (Short Warwick Edinburgh Mental Wellbeing Scale; SWEMWBS; [4]): The SWEMWBS measures mental wellbeing via seven positively phrased statements, concerning specific thoughts and feelings. Respondents are asked to indicate how often they experienced each thought/ feeling over the previous two weeks on a 5-point Likert-type scale, ranging from with 1 (“None of the time”) to 5 (“All of the time”). Example items include “I’ve been feeling optimistic about the future” and “I’ve been dealing with problems well”. Raw scores are transformed into interval scale scores (see [4]) and yield a potential total range of 7 to 35-points. The SWEMWB has been validated for use among the general population [5] and demonstrated good reliability (α = .89) in our sample at T1.
- Burnout (Maslach Burnout Inventory;[6]): The 22-item inventory measures job-related burnout via three separate dimensions, including the nine-item emotional exhaustion subscale, the five-item depersonalisation subscale, and the eight-item personal accomplishment subscale. Participants are asked to indicate how often each statement describes the way they feel about working as a clinician, on a 7-point Likert-type scale, ranging from 0 (“Never”) to 6 (“Every day”). Items on the emotional exhaustion (e.g., “I feel emotionally drained from my work”) and depersonalisation (e.g., I feel I treat some clients as impersonal objects”) subscales are negatively phrased, while statements on the personal accomplishment subscale (e.g., “I can easily understand how clients feel about things”) are positively phrased. The three subscales should be calculated separately, yielding potential total ranges of 0 to 54 for emotional exhaustion, 0 to 30 for depersonalisation and 0 to 48 for personal accomplishment. The psychometric properties of the measure are well established (see [6]) and within our sample, the emotional exhaustion subscale demonstrated excellent reliability (α = .91) and the depersonalization (α = .76) and personal accomplishment (α = .76) subscales demonstrated acceptable reliability at T1.
- Mindfulness (15-item version [minus ‘observe’] of the Five Facets of Mindfulness Questionnaire, FFMQ-15; [7]): Mindfulness was measured across a four-factor structure of describing, acting with awareness, non-judgement and non-reactivity. Based on recommendations made by Gu and colleagues [7], a fifth factor (observing) was excluded and thus a total of 12-items was used. Each item is presented as either a positively or negatively phrased statement, and respondents are asked to indicate how true each statement generally is of them on a 5-point Likert-type scale, ranging from 1 (“Never or rarely true”) to 5 (“Very often or always true”). Example items include “I’m good at finding words to describe my feelings” (describing item), “I don’t pay attention to what I’m doing because I’m daydreaming, worrying, or otherwise distracted” (acting with awareness item), “I believe some of my thoughts are abnormal or bad and I shouldn’t think that way” (non-judgement item) and “When I have distressing thoughts or images I am able just to notice them without reacting” (non-reactivity item). Before computing a total score, negatively phrased items are reverse scored, yielding a potential total range of 12 to 60-points, with higher scores indicating increased mindfulness. Gu and colleagues [7] evaluation of the measure supports its use and it demonstrated good reliability (α = .83) in our sample at T1.
- Self-compassion (Self-Compassion Scale-Short-Form, SCS-SF; [8]): The SCS-SF consists of twelve items and respondents are asked to indicate how often they behave in the stated manner for each item on a 5-point Likert-type scale ranging from 1 (“Almost never”) to 5 (“Almost always”). The SCS-SF yields a total self-compassion score that is based on six-factors of self-kindness (e.g., “When I’m going through a very hard time, I give myself the caring and tenderness I need”), self-judgement (e.g., “I’m disapproving and judgmental about my own flaws and inadequacies”), common humanity (e.g., “I try to see my failings as part of the human condition”), isolation (e.g., “When I’m feeling down, I tend to feel like most other people are probably happier than I am”), mindfulness (e.g., “When something upsets me I try to keep my emotions in balance”) and over-identification (e.g., “When I’m feeling down I tend to obsess and fixate on everything that’s wrong.”). Negatively phrased items are reverse scored, yielding a potential total range of 12 to 60-points, with higher scores indicating increased self-compassion. The SCS-SF is reported to have good psychometric properties [8] and demonstrated good reliability (α = .88) in our sample at T1.
- Compassion for others (Compassionate Love Scale, CLS; [9]): The 21-item CLS can be used to measure either compassion for close others or strangers/ humankind more broadly, with the latter version being used in the present study. Respondents are asked to indicate the extent to which they feel each of the positively phrased statements is true of them, on a 7-point Likert-type scale ranging from 1 (“Not at all true of me”) to 7 (“Very true of me”). Example items include “When I see people I do not know feeling sad, I feel a need to reach out to them” and “I spend a lot of time concerned about the well-being of humankind”. Means are computed to yield a potential total score ranging from 1 to 7. The CLS has been shown to have good psychometric properties [9] and demonstrated excellent reliability (α = .95) in our sample at T1.
- Worry (Penn State Worry Questionnaire, PSWQ; [10]): The 16-item PSWQ assesses one’s relationship with and tendency to engage in generalised, excessive and uncontrollable worry. Respondents are asked to indicate how typical each of the statements is of them on a 5-point Likert-type scale ranging from 1 (“Not at all typical of me”) to 5 (“Very typical of me”). Some items are positively phrased (e.g., “I do not tend to worry about things”) while others are negatively phrased (e.g., “My worries overwhelm me”). Positively phrased items are reverse scored before computing a total score with a potential range of 16 to 80-points, with higher scores indicating increased worry. The PSWQ has been found to have good psychometric properties (see [10]) and demonstrated excellent reliability (α = .94) in our sample at T1.
- Rumination (Brooding subscale of the Ruminative Response Scale, RRS; [11]): The 22-item RRS assesses ruminative responses to depressed mood via three dimensions of depression, reflection, and brooding. It has been suggested that investigators should analyse the subscales separately [12] and in the present study, we only utilised the brooding subscale. Brooding has been defined as “a passive comparison of one’s current situation with some unachieved standard” ([12] p. 256) and is considered maladaptive and associated with present and future increases in depression. The Brooding subscale consists of five negatively phrased ways of thinking and respondents are asked to indicate how often they generally think in the stated ways on a 4-point Likert-type scale ranging from 1 (“almost never”) to 4 (“almost always”). Example items include “What am I doing to deserve this?” and “Why do I always react this way?”. Treynor and colleagues [12] suggest that the subscale is moderately reliable, and it demonstrated good reliability (α = .81) in our sample at T1.
- Sickness absence measured at T1 and T3 was assessed using one-item that asked participants to report how many days they had been absent from work due to sickness during the previous three months (i.e., during the three-months prior to participation and during the three-month study period).

Demographic information

Demographic information assessed at T1 included participants’ age, gender, marital status, number of children under 18 years, number of children aged 18 years or over, NHS job role, trust and team, number of hours worked per week in said NHS job role, highest level of education, individual and household annual incomes, ethnicity, and perceived relative socio-economic status (SES) [13], with response options from 1 (lowest) and 10 (highest) perceived SES.

## Intervention expectations and experience

- Intervention expectancy at T1 (Credibility/Expectancy Questionnaire, CEQ; [14]): The CEQ is a six-item questionnaire designed to assess participants views on the credibility of a treatment and their expectations of its effects. Items 1, 2 and 3 assess credibility by asking participants to indicate how logical the therapy seems; how successful they think the treatment will be in reducing their symptoms; and how confident they would be in recommending said treatment to a friend experiencing similar problems. Participants are asked to respond to each of these items on a 9-point Likert-type scale ranging from 1 (“not at all logical/ successful/ confident”) to 9 (“very logical/ successful/ confident”). Items 4, 5 and 6 assess expectancy, asking respondents to indicate how much symptom improvement they expect, how much they really feel that therapy will improve their symptoms and how much improvement in symptoms they really feel will occur. Participants are asked to respond to items 4 and 6 from 11 percentage options, increasing in units of ten from 0% to 100%. Question 5 alternatively asks participants to respond on a 9-point Likert-type scale ranging from 1 (“Not at all”) to 9 (“Very much”). Raw scores are converted into standardised residuals (z-scores) before computing totals for the separate sub-scales. In the present study, the words “treatment” and “therapy” were exchanged for “intervention”. The CEQ has been found to demonstrate high internal consistency and good test-retest reliability (see [14]) and the credibility (α = .81) and expectancy (α = .92) subscales demonstrated good-to-excellent reliability in our sample.
- Self-reported intervention engagement at T2 and T3. Three questions were used to assess intervention engagement: (1) formal engagement was assessed by asking participants to self-report the average number of days per week they had spent following a guided mindfulness meditation on Headspace/a recommended stress-management or wellbeing strategy accessed via the Moodzone webpage; (2) on these days, participants were asked to report on average, how many minutes per day they spent formally engaging; and (3) informal engagement was assessed by asking participants to self-report the average number of days per week they had brought mindfulness to a daily activity or brought the recommended stress-management or wellbeing strategies accessed via Moodzone into their daily life. At T2, these questions were asked in relation to the previous month and at T3 they were asked in relation to the previous three months.
- Intervention evaluations at T2 and T3: Participants were asked ‘how likely they were to recommend the intervention to friends and family’, on a five-point scale ranging from 1 “extremely unlikely” to 5 “extremely likely”; how much they really felt that their allocated intervention had helped their wellbeing on a scale from 1 (“not at all”) to 9 (“very much”); and how likely they were to continue practicing mindfulness (Headspace participants) or stress management/wellbeing strategies (Moodzone participants) over the following six-months, on a scale from 1 (“not at all”) to 9 (“definitely”).

## Protocol checks

- Hypothesis guess at T3: Participants were asked to state, in their own words, what they thought the purpose of the study was and this was coded as ‘correct guess’ if they were aware of the direction of effect between the arms, otherwise this was coded as ‘incorrect guess; or ‘no response’.
- Intervention deviations at T3: Participants were asked to indicate whether or not they had engaged with the alternative study intervention (i.e. Headspace/Moodzone) during the course of the study. If participants answered ‘yes’ to this question, they were asked to approximate the number of days spent doing so during the study period.
- Prior mindfulness experience at T3: Participants were asked to indicate their experiences of mindfulness prior to taking part in the study, including; whether or not they had ever taken part in at least 4 sessions of an MBCT or MBSR course, whether they had ever taken part in an MBSH course (and if so to provide details); if they had ever engaged with Headspace (and if so to provide details); and how often they had practiced mindfulness on a scale from 1 (“never”) to 8 (“daily”).

## Serious adverse events and lasting negative effects

Serious adverse events were recorded in line with NIHR Good Clinical Practice guidelines [15]. Participants were also asked to indicate the extent to which they agreed/disagreed that they had experienced “lasting bad effects” from using their allocated intervention, on a scale from 1 (“strongly agree”) to 5 (“strongly disagree”) at T3. If participants agreed or strongly agreed, they were asked to provide further details, including the aspects of the intervention they felt contributed to these effects, via pre-specified and an ‘other’ free-text response option, and any further details they would like to provide.

## References

1. Lovibond PF, Lovibond SH. The structure of negative emotional states: Comparison of the Depression Anxiety Stress Scales (DASS) with the Beck Depression and Anxiety Inventories. Behaviour Research and Therapy 1995 Mar;33(3). [doi: 10.1016/0005-7967(94)00075-U]

2. Henry JD, Crawford JR. The short-form version of the Depression Anxiety Stress Scales (DASS-21): Construct validity and normative data in a large non-clinical sample. British Journal of Clinical Psychology 2005 Jun;44(2). [doi: 10.1348/014466505X29657]

3. Antony MM, Bieling PJ, Cox BJ, Enns MW, Swinson RP. Psychometric properties of the 42-item and 21-item versions of the Depression Anxiety Stress Scales in clinical groups and a community sample. Psychological Assessment 1998 Jun;10(2). [doi: 10.1037/1040-3590.10.2.176]

4. Stewart-Brown S, Tennant A, Tennant R, Platt S, Parkinson J, Weich S. Internal construct validity of the Warwick-Edinburgh Mental Well-Being Scale (WEMWBS): A Rasch analysis using data from the Scottish Health Education Population Survey. Health and Quality of Life Outcomes 2009 Feb 19;7. PMID:19228398

5. Ng Fat L, Scholes S, Boniface S, Mindell J, Stewart-Brown S. Evaluating and establishing national norms for mental wellbeing using the short Warwick–Edinburgh Mental Well-being Scale (SWEMWBS): findings from the Health Survey for England. Quality of Life Research 2017 May 16;26(5). [doi: 10.1007/s11136-016-1454-8]

6. Maslach C, Jackson SE, Leiter M. The Maslach Burnout Inventory [Internet]. 1986. Available from: https://www.researchgate.net/publication/277816643

7. Gu J, Strauss C, Crane C, Barnhofer T, Karl A, Cavanagh K, Kuyken W. Examining the factor structure of the 39-item and 15-item versions of the Five Facet Mindfulness Questionnaire before and after mindfulness-based cognitive therapy for people with recurrent depression. Psychological Assessment 2016 Jul;28(7). [doi: 10.1037/pas0000263]

8. Raes F, Pommier E, Neff KD, van Gucht D. Construction and factorial validation of a short form of the Self-Compassion Scale. Clinical Psychology and Psychotherapy 2011 May;18(3):250–255. PMID:21584907

9. Sprecher S, Fehr B. Compassionate love for close others and humanity. Journal of Social and Personal Relationships 2005 Oct;22(5):629–651. [doi: 10.1177/0265407505056439]

10. Meyer TJ, Miller,’ ML, Metzger~ RL, Borkovec’ TD. DEVELOPMENT AND VALIDATION OF THE PENN STATE WORRY QUESTIONNAIRE. Behae Res Ther. 1990.

11. Nolen-Hoeksema S, Morrow J. A prospective study of depression and posttraumatic stress symptoms after a natural disaster: The 1989 Loma Prieta earthquake. Journal of Personality and Social Psychology 1991;61(1). [doi: 10.1037/0022-3514.61.1.115]

12. Treynor W. Rumination Reconsidered: A Psychometric Analysis. Cognitive Therapy and Research 2003;27(3). [doi: 10.1023/A:1023910315561]

13. Adler N, Stewart J. The MacArthur Scale of Subjective Social Status. MacAurthur Research Network on SES and Health. 2007.

14. Devilly GJ, Borkovec TD. Psychometric properties of the credibility/ expectancy questionnaire. Journal of Behavior Therapy and Experimental Psychiatry. 2000.

15. National Institute for Health Research. Clinical Trials Guide. Clinical Trials Guide. 2019.
