# Supplementary material for: Health Care Workers’ Need for Headspace: Findings From a Multisite Definitive Randomized Controlled Trial of an Unguided Digital Mindfulness-Based Self-help App to Reduce Healthcare Worker Stress
Source: JMIR Mhealth Uhealth. 2022 Aug 25;10(8):e31744. doi: 10.2196/31744 (PMC9459942; doi:10.2196/31744)
Supplement: Multimedia Appendix 2 [file mhealth_v10i8e31744_app2.pdf]

Study Name: MindSHINE 3: A definitive randomised controlled trial investigating two online wellbeing interventions to reduce NHS staff stress
Date: 03/05/2018
Version: 8
IRAS ID: 210175

Participant Information Sheet

MindSHINE 3: A definitive randomised controlled trial investigating two online wellbeing interventions to reduce NHS staff stress

Would you like to take part in this research study? Before you decide, it is important for you to understand why the research is being done and what it would involve. Please take time to read the following information and to decide whether or not you wish to take part. Please feel free to ask us if there is anything that is not clear or if you would like more information (please see our contact details at the end of this document).

Brief summary

We are investigating the effectiveness of two online interventions in improving wellbeing and reducing stress among NHS staff. We are recruiting around 2000 NHS employees to take part, across a range of NHS trusts and GP practices in England. Half of these participants will be randomly allocated to an intervention that will require the downloading of a smartphone app and internet access and the other half will be randomly allocated to an intervention that will require the personal use of a computer/ tablet with internet access.

Participants in both groups will be asked to spend a minimum of 10 minutes per day accessing their respective interventions. This will involve reading information, watching videos/ listening to audio- files and carrying out exercises. Initially the intervention will last for a period of 30 days, but participants will be invited to continue to use the intervention materials for a further 90 days following this. Participants will also be asked to complete online questionnaires before being randomised to one of the two interventions, at 35-days post-randomisation, and then again at 125- days post-randomisation. Participants will be emailed with links to these online surveys, and depending on group allocation, may also be sent emails and smartphone messages to encourage them to engage with the interventions. It should take around 20 minutes to complete the questionnaires at each time point.

What is the purpose of the study?

Stress experienced by NHS staff can have many negative consequences to them as individuals, to their patients and to the healthcare sector more broadly. At an individual level, healthcare staff may experience reduced self-esteem, fatigue, poor job-satisfaction and burnout. In turn, this can negatively impact patients via a reduced quality of patient care and prolonged recovery for patients and stress, anxiety and depression are significant causes of sickness absence among NHS staff. As such, it is considered very important to provide evidence-based interventions to support the wellbeing of NHS staff. The purpose of the present study is therefore to investigate the effectiveness of two existing interventions in improving wellbeing and reducing psychological distress among NHS staff. We are also interested in investigating how, and for whom such interventions exert benefits. The present study is specifically investigating self-help based interventions, in order to provide flexible access to NHS staff who, due to work commitments, may not otherwise be able to attend face-to-face interventions. If this study is successful, the research team will support the roll-out of a self-help based intervention designed to support well-being and reduce psychological distress across the NHS.

Am I eligible to take part in this study?

The only requirements for participation are that you:

1. are aged 18 years or over;
2. are currently employed in an NHS trust or GP practice in England;
3. are working in a role that involves direct contact with patients for at least one day per week. If you are not a clinician this could apply to you if you have direct contact with patients at least one day a week on average (e.g. if you are an administrator working on a reception desk in a clinic);
4. are currently in work (i.e. not currently on sick leave);
5. have sufficient English Language reading and listening ability to undertake a course that is taught through materials written and spoken in English;
6. are not currently undertaking any other psychological interventions and will refrain from undertaking any interventions during the course of the study;
7. own an Apple or Android smartphone/ tablet, or a computer with internet access.

You do not need to be experiencing distress or mental health difficulties to take part in this study. This study is for any NHS member of staff who works directly with patients.

Please note that self-help interventions have been found to be helpful for people experiencing mild to moderate symptoms of psychological distress, and may not be helpful for people who are currently experiencing more severe forms of psychological distress. If you are currently experiencing significant mental health problems and are unsure whether the course would be useful for you, you may wish to discuss the study with your GP or a mental health professional. Alternatively, you can contact the research team to discuss any concerns you might have.

Do I have to take part?

It is up to you to decide whether or not to take part. If you decide not to take part this will not affect terms and conditions of your employment. If you decide to take part, you will be asked to indicate your consent online. You will be free to withhold any personal information or to withdraw at any time, without giving a reason and without this affecting the terms and conditions of your employment. If you wish to remove your data from the study, you can do so up until 1st March 2019, by contacting the research team. If you do decide to take part, we will ask you to refrain from using any other psychological interventions or psychological self-help materials during the course of the study, in order that we may evaluate the interventions offered in the present study.

Do I need to inform my manager if I decide to take part?

You will only need to inform your manager that you are taking part if you choose to use the intervention materials during working hours.

What would taking part involve?

If you decide that you want to take part in this study, you will need to provide informed consent online. The consent form will also ask you for optional permission for the research team to ask your HR department for the number of days you have been off sick in the three months prior to your taking part in the study and in the three months following the intervention. This is because we are interested in whether taking part in the interventions has an impact on staff sickness absence. Sickness absence information will be coded and your name and contact details will be removed and stored on a separate password protected file. In the event that a participant wish to remove their data from the study, unique identity codes can be linked back to participants' names and contact details to enable this process. However, if you do not agree to the release of this sickness absence data to the research team, you can still take part in the study.

When we have received your consent, you will be emailed with a unique identifier code and a link to complete some pre-intervention questionnaires. You will be asked to input this code into all subsequent questionnaires in order to protect your privacy. The first set of online questionnaires will ask you:

1. About your recent experiences of stress, anxiety and depression

2. About your compassion towards yourself and others

3. How mindful you are in everyday life

4. About your wellbeing

5. About your levels of work-related burnout

6. About your experiences of rumination and worry

7. Your expectations of the intervention

You will also be asked some questions about your age, gender, ethnicity, marital status, NHS job role, name of the NHS trust or GP practice you work, name of the NHS team you work in, your NHS working hours, number of children, household income, individual income, highest level of education, socioeconomic status and sickness absence.

These questionnaires should take around 20 minutes to complete on each occasion.

Once you have completed these initial measures, you will be randomly allocated to one of the two conditions.

If you are one of the first 50 participants to be allocated to one of the groups, you will also be asked to inform us about your beliefs about engaging with the intervention. This should take around 5 minutes to complete and will also be carried out online.

The first 25 participants will be asked these questions before the intervention begins and the second 25 participants will be asked these questions at 35-days post-randomisation.

Once all baseline measures are completed, you will be sent an email informing you of your random allocation and instructions on how to proceed with your given intervention.

We will send you most of the above questionnaires again after 35-days and then again 90 days later. The only difference is that we will not need to ask you about demographics and expectations of the intervention again. Instead we will ask you to report back on your engagement with the interventions and in the final set of questionnaires we will also ask you to provide us with information about your prior experiences with psychotherapeutic interventions and self-help materials. These questionnaires should again take about 20 minutes to complete at each time point.

What will happen once I have been allocated to an intervention?

Once you have been allocated to an intervention, you will be sent an email with instructions on how to access the intervention. Both interventions will require you to own an Apple or Android smartphone/tablet, or a computer with internet access. Both interventions will offer a variety of written information, videos, audio-tracks and exercises for you to complete. You will be asked to engage with these intervention materials for a minimum of 10 minutes per day for an initial period of 30-days, but will be encouraged to continue doing so until the end of the study at 125-days following randomisation. If you are allocated to the group requiring smartphone access you may also be sent some emails and smartphone messages, encouraging you to engage with the intervention. For participants in both groups, emails containing links to online questionnaire sites will be sent at 35-days and 125-days post-randomisation.

You will be given access to the alternative (non-allocated) intervention at the end of the study to thank you for your participation.

How much time is involved in taking part in the study?

We ask participants to engage with intervention materials for a minimum of 10 minutes per day across an initial period of 30-days, and encourage participants to

continue doing this until the end of the study at 125-days post-randomisation. Reading the information sheet, asking questions and providing informed consent may take up to 30 minutes to complete and following instructions to get started with your given intervention may take a further 10 minutes. The questionnaires should take approximately 20 minutes each to complete, on three separate occasions. If you are one of those 50 participants selected to complete the additional 3 items concerning beliefs about engaging with the intervention, this may take up to an additional 5 minutes on one occasion only.

Where will I have to go?

The interventions are designed to be completed at times and locations that are convenient for you.

What are the advantages and disadvantages of taking part?

Stress-reduction interventions use a variety of techniques to improve psychological wellbeing and reduce psychological distress. Such interventions can lead to increased self-acceptance, self- compassion, and can enable participants to foster a non-judgmental attitude. Stress reduction interventions may also increase attention to the present moment, reducing focus on past worries and future concerns and helping people to let go of unpleasant experiences. It may also decrease symptoms of stress, anxiety and depression. By taking part in this research you will also be given the opportunity to be entered into a prize draw to win one of five £50 Amazon vouchers.

Because the interventions used in the present study have not previously been evidenced with healthcare workers, by participating in this study you will be helping us to identify whether or not the interventions under investigation can be of benefit to NHS staff, so that the NHS may be better informed to support their employees.

Some of the intervention techniques may encourage you to reflect on your thoughts, feelings and experiences. While this process can be helpful, it can also sometimes feel difficult. In doing so you may become aware of some unpleasant thoughts, feelings and/or experiences, but this is completely normal. The self-help guides provide advice on ways of coping with stress. However, if you are feeling distressed and that you need additional advice or support, contact details of organisations that you may find useful are provided on the last page of this information sheet. You may also wish to contact your GP for further guidance.

Please note that the interventions used in this study are unguided self-help, and the research team are not able to offer individual support.

This study has received approval from the University of Sussex ethics committee and the Health Research Authority.

Confidentiality

All information collected will be kept strictly confidential and stored securely. Anonymity will be ensured in the publication and presentation of findings. Only members of the research team and regulatory authorities will have access to information gathered through the study. This information will be coded and your name and contact details will be stored separately from all other data on a password protected file. In the event that a participant wish to remove their data from the data file, unique identifying codes can be linked back to a participant’s name and contact details to enable this process. The study complies with data protection laws. Anonymised data will be kept for 10 years as researchers may wish to return to the data in the future.

What will happen to the results of the study?

The results of this study will be written up for publication and presentation and will form part of a doctoral thesis. You can choose to receive feedback on the results of this study. No-one will be identified in any publication or presentation.

Who has reviewed the study?

The research has been approved by the Sciences and Technology Cross-Schools Research Ethics Committee (Sci-Tec C-REC) at the University of Sussex and the Health Research Authority.

Next Steps

If you are interested in taking part in the study, please take your time to consider your decision before completing the online consent form presented on the next page. This is to ensure that you have had time to consider your decision.

If you would like more information about this study, please contact the research team at mindshine@sussex.ac.uk

If you choose to email any questions, we will aim to respond within 48 hours.

The research lead for this study is: Heather Taylor, School of Psychology, Pevensey 1 University of Sussex, Falmer, East Sussex Email: ht207@sussex.ac.uk

Please also feel free to contact Dr Clara Strauss (c.y.strauss@sussex.ac.uk) or Dr Kate Cavanagh (kate.cavanagh@sussex.ac.uk) who are the academic supervisors for this study.

Should you have concerns in relation to your psychological wellbeing during this study you may wish to let us know. If you do have concerns about your psychological wellbeing we would encourage you to contact your GP for advice and/or support. You may also wish to discuss your concerns with your manager.

Alternatively, Mind (08457 90 90 90; http://www.mind.org.uk/) provides information, advice, and support for people experiencing psychological distress.

The University of Sussex has arrangements in place for payment of compensation in the event of harm to the research participants where no legal liability arises. If you decide to participate in the study you will be given an email receipt of your informed consent and a copy of this information sheet.

For further information about this research please contact Heather Taylor (ht207@sussex.ac.uk) or the project supervisor's Dr Kate Cavanagh (kate.cavanagh@sussex.ac.uk) or Dr Clara Strauss (clara.strauss@sussex.ac.uk) This research has been approved (ER/HT207/8) by the Sciences & Technology Cross-Schools Research Ethics Committee (Sci-Tec C-REC). If you have any ethical concerns, please contact the ethics chair (crecscitecchair@sussex.ac.uk)OR project supervisors (kate.cavanagh@sussex.ac.uk or clara.strauss@sussex.ac.uk). University of Sussex has insurance in place to cover its legal liabilities in respect of this study.

Study Name: MindSHINE 3: A definitive randomised controlled trial investigating two online wellbeing interventions to reduce NHS staff stress
Date: 03/05/2018
Version: 8
IRAS ID: 210175

CONSENT FORM

Study Title: MindSHINE 3: A definitive randomised controlled trial investigating two online wellbeing interventions to reduce NHS staff stress

Chief Investigator: Heather Taylor

Please insert your full name here:

[ ]

Please insert the name of your NHS trust or GP practice here:

[ ]

Please insert the name of your NHS team here:

[ ]

Please insert the email address you would like to use for correspondence and intervention participation during the study. All communications with regard to the study must be made using the same email address and as such it should be an email address that you will regularly access. Due to potential technical issues, please avoid using Hotmail, Outlook, MSN and/ or Live email addresses wherever possible:

[ ]

The following are the terms of agreement you will need to undertake in participating in this study. Please click on the 'agree' button for all that apply. Please note that it is not essential that you agree to item 15 or item 16, and that you will still be eligible to take part if you choose not to consent to these items. All other items must be agreed to in order for you to participate in this study.

1) I confirm that I have read and understood the participant information sheet dated 03.05.2018 for the above study and have had the opportunity to ask questions of the research team

- ○ Agree

2) I confirm that I have had sufficient time to consider whether or not I want to be included in this study.

- ○ Agree

3) I understand that my participation is voluntary and that I am free to withhold personal information or to withdraw at any time, without giving any reason, and without my legal rights being affected.

- ○ Agree

4) I understand that if I choose to terminate my participation before the end of the study period (125-days) that any questionnaires I have already completed will be kept by the research team, unless I contact them wishing for my data to be removed from the dataset. The deadline for data withdrawal is 1st March 2019.

- ○ Agree

5) I understand that both identifiable and de-identified data collected during the study may be looked at by individuals from regulatory authorities, the NHS Trust or GP practice and/ or the University of Sussex, where it is relevant to my taking part in this research. These include members of the Research and Development Governance team who are hosting the study (Sussex Partnership), the Health Research Authority and the study sponsor (University of Sussex). I give permission for these individuals to have access to my records.

- ○ Agree

6) I give permission for findings from the study to be written up for publication and presentation at conference. Any publication or presentation would not identify me.

- ○ Agree

7) I give permission for non-identifiable data to be shared with other research teams for research purposes.

- ○ Agree

8) I confirm that I am aged 18 years or older.

- ○ Agree

9) I confirm that I am not currently undertaking any other psycho-therapeutic intervention and will refrain from doing so during my time participating in the study (125-days).

- ○ Agree

10) I confirm that I have sufficient English language ability to read and listen to intervention materials and provide responses to questionnaires.

- Agree

11) I confirm that I am currently employed within an NHS trust or GP practice in England in a role that consists of direct patient-contact for a minimum of one day per week

- Agree

12) I confirm that I am currently in work (i.e. not on long-term sickness absence)

- Agree

13) I confirm that I have regular personal access to an Apple or Android smartphone/tablet or to a computer with internet access

- Agree

14) I agree to take part in the above study

- Agree

OPTIONAL CONSENT ITEMS

The following two items are optional and if you choose not to tick one or both of these items you will still be able to participate in the study

15) I understand that, as part of this study and with my consent, the study research team will ask Human Resources (HR) to release my sickness absence records for the three months preceding my participation in the study intervention period and for the three months following my participation in the study intervention period. I give permission for HR to release my sickness absence records to the study research team. I understand that these records will be identified using my personal details (i.e. name) but that HR/ the research team will assign a unique identifying code to this information to be used during data analysis. My personal information will be stored on a separate password protected file, but can be linked to my assigned unique identifying code in the event that I wish to remove my data from the study

- Agree

16) Please tick this box if you would like to be entered into a prize draw to win one of five £50 Amazon vouchers

- ○ Agree

For further information about this research please contact Heather Taylor (ht207@sussex.ac.uk) or the project supervisor’s Dr Kate Cavanagh (kate.cavanagh@sussex.ac.uk) or Dr Clara Strauss (clara.strauss@sussex.ac.uk) This research has been approved (ER/HT207/8) by the Sciences & Technology Cross-Schools Research Ethics Committee (Sci-Tec C-REC). If you have any ethical concerns, please contact the ethics chair (crecscitecchair@sussex.ac.uk)OR project supervisors (kate.cavanagh@sussex.ac.uk or clara.strauss@sussex.ac.uk). University of Sussex has insurance in place to cover its legal liabilities in respect of this study.

Participant Debrief Information

Thank you for taking part in the study. Before participating in this research you were informed that the purpose of the study was to compare the effects of two interventions targeted at improving psychological well-being and decreasing levels of psychological distress. More specifically, we were investigating the effects of a mindfulness-based self-help smartphone application called Headspace compared to an NHS accredited 'standard care' website, Moodzone.

Mindfulness-based interventions have been shown to be effective in improving a wide array of mental health and well-being outcomes in both clinical and non-clinical populations. More specifically, a face-to-face group mindfulness programme has been found to be effective in decreasing stress and improving self-compassion among NHS staff, while a mindfulness-based self- help intervention has been found to be effective in decreasing levels of stress and anxiety among medical students. As such, the primary purpose of this study was to see whether participation in Headspace would lead to significant improvements on a variety of measures of psychological health and wellbeing among NHS staff compared to standard care.

We also wanted to investigate how and for whom a mindfulness-based self-help intervention such as Headspace may be effective. Research concerning standard face-to-face mindfulness-based interventions has shown that the relationship between mindfulness-based intervention participation and positive post-intervention outcomes is mediated by increases in mindfulness and self- compassion, and decreases in rumination and worry. Because mindfulness-based self-help differs in structure, content and format to face-to-face mindfulness-based interventions, we wanted to see if the same mechanisms were evident in Headspace. Moreover, it has been proposed that greater levels of engagement in mindfulness-based interventions leads to greater post-intervention outcomes, and so we wanted to test this theory and investigate whom does and does not engage well with this type of intervention.

Once all participants have completed the study, a summary of study findings will be emailed to each participant and winners of the prize draw will be notified. Findings from the study will also be presented at Trust research conferences and seminars to which staff are invited. The results of the study will be written up and submitted for publication in a peer reviewed journal. Participants will not be identifiable from any summary of findings or papers written for publication.

Thank you again for taking part.
