# Supplementary material for: Health Care Workers’ Need for Headspace: Findings From a Multisite Definitive Randomized Controlled Trial of an Unguided Digital Mindfulness-Based Self-help App to Reduce Healthcare Worker Stress
Source: JMIR Mhealth Uhealth. 2022 Aug 25;10(8):e31744. doi: 10.2196/31744 (PMC9459942; doi:10.2196/31744)
Supplement: Multimedia Appendix 3 [file mhealth_v10i8e31744_app3.docx]

# Appendix 3: Handling Missing Data

At the item level, there were very few missing values within questionnaires. Missing values for missing items were imputed (using a single imputation) using predictive mean matching in mice [62]. Questionnaires were scored only if fewer than 1/3 of items were missing. For the DASS-21 subscales, a single score was imputed for 3 participants at baseline and 2 at post. Thus 5 of 21 $\times$ 4722 = 99162 scores were imputed. In short, a negligible amount.

At the scale level multiple imputation was used to handle missing values. Specifically, the model described below was fit using an intention to treat (ITT) analysis using multiple imputation using chained equations. A dataframe was created that included all time-variant measures and the following time-invariant variables: trust type, trial arm, ethnicity, job role, hours worked, education, marital status, perceived socioeconomic status (SES), age, sex, income (individual), and income (household). The time variant measures were completion time (in months) and the scale totals including both the primary (DASS-21 Stress) and secondary (formal and informal engagement, sickness absence, DASS-21 Depression, DASS-21 Anxiety, FFMQ-15 Mindfulness minus Observe, SCS-SF Self-Compassion, all Maslach Burnout Inventory subscales, PSWQ Worry, SWEMWBS Wellbeing [scaled], all RRS subscales and CLS Compassion for Others) outcomes. Thirty imputed datasets were created, and imputations were performed using the mice package [62]. The imputation model was based on logistic regression for binary variables (gender and hours worked), polytomous logistic regression for categorical variables (marital status, job role) and a proportional odds model for ordered categorical variables (education, individual and household income). Having imputed 30 data sets, the model described in the model selection section was fitted to each using the lme4 package [61] and then pooled using mice [62]. Interaction terms were followed up by estimating the marginal means from the multiply imputed model with months set to the average at the initial intervention and post-intervention phases (1.5 and 4.5 months respectively) and then setting contrasts that compared each time point to baseline separately in the two arms. This analysis of estimated marginal means was conducted using the emmeans package [1] with degrees of freedom computed with Satterthwaite’s method.

## References

1. Lenth R. Emmeans: Estimated marginal means, aka least-squares means. 2020.
