# Supplementary material for: Health Care Workers’ Need for Headspace: Findings From a Multisite Definitive Randomized Controlled Trial of an Unguided Digital Mindfulness-Based Self-help App to Reduce Healthcare Worker Stress
Source: JMIR Mhealth Uhealth. 2022 Aug 25;10(8):e31744. doi: 10.2196/31744 (PMC9459942; doi:10.2196/31744)
Supplement: Multimedia Appendix 4 [file mhealth_v10i8e31744_app4.docx]

# Appendix 4: Additional Data Analysis Plan, Results and Discussion

This document contains further details of the data analysis plan and details of the analysis and interpretation of secondary outcomes.

# Additional Data Analysis Plan

**Reliable Change**

Reliable change in DASS-21 Stress was calculated for participants scoring in at least the mild range at baseline (>14) as the difference in scores between two time points relative to the spread of scores (adjusted for scale reliability) at baseline (i.e. in the absence of change) [1][68]:

$$\text{RC}=\frac{X_{\text{post}}-X_{\text{baseline}}}{s_{\text{baseline}}\sqrt{2}\sqrt{1-\alpha}}$$

Reliable change at T2 and T3 was predicted using two separate polytomous logistic regression models with no reliable change as the reference category and trial arm as the predictor. Each model was fitted to the 30 imputation samples and model parameter estimates and their standard errors were pooled across these.

Exploratory mediation analyses were conducted which were not pre-registered. For these we used the PROCESS add-in for SPSS [2]. Five thousand bias corrected bootstrapped samples were used to estimate indirect effects of the independent variable (trial arm) on the dependent variable (DASS-21 Stress standardised residual change scores T1-T3, the primary outcome) via proposed mediators (formal engagement measured as days/week with intervention practices/tasks undertaken between T1-T2 and T1-T2 standardised residual change scores on the FFMQ-15 Mindfulness, SCS-SF Self-Compassion, PSWQ Worry and RRS Brooding). The mediation analyses included intervention completers only (defined as formal engagement with the intervention on at least 3 days/week during the T1-T2 initial intervention period [2]) in line with Kazdin’s [3] recommendations. The analysis also satisfied the requirement that the mediator is measured prior to the outcome [3]

Randomization check

Although not included in the pre-registration, the success of randomisation in balancing arms was assessed using Bayes factors. Bayes factors were estimated for each demographic variable and baseline outcome measure that compared the two trial arms. The BayesFactor package was used [4]. For continuous outcomes the scale factor for the prior distribution was set at 1, which represents a 50% probability that the effect size (Cohen’s d) lies between ±1 [5]. This prior is relatively uninformative (because randomisation means we have no specific prediction about effects). For categorical variables, an independent multinomial model was fit that assumes that the totals within trial arm are fixed (which is consistent with randomisation) using the Gunel and Dickey method [6,7]. All Bayes factors were computed such that values below 1 suggest shifting beliefs towards the null, and values above 1 suggest shifting beliefs towards the alternative hypothesis.

# Additional Results

## Secondary Outcomes

Trial arm x time effects on all outcomes except for sickness absence for the ITT are shown in Table 3 in the main manuscript and for the per protocol analysis in Supplementary Material 7.

Mental health outcomes: Depression, Anxiety, Wellbeing In the ITT sample, the trial arm x months interactions show that Headspace led to significantly greater improvement in depression (*b* = 0.24), anxiety (*b* = 0.19) and wellbeing (*b* = 0.14) over time than Moodzone. This means that there was a between-groups difference in the rate of improvement over time of 0.24 points per month (DASS-21 Depression), 0.19 points per month (DASS-21 Anxiety) and 0.14 points per month (SWEMWS Wellbeing) for Headspace participants compared to participants in the Moodzone arm. For depression, anxiety and wellbeing between-group effects were significant at 1.5 months and 4.5 months (see Table 3 in the main manuscript for details). For the per protocol sample, effects of trial arm over time on all of these outcomes were non-significant.

Proposed mechanisms of action: Mindfulness, Self-compassion, Rumination, and Worry The trial arm × months interaction, shows that Headspace led to a significantly greater improvement in mindfulness (*b*=0.22), self-compassion (*b*=0.48) and worry (*b*=0.30) over time than Moodzone in the ITT sample. For mindfulness and worry, between-group effects were non-significant at 1.5 months but significant at 4.5 months. For self-compassion, between-group effects were significant at 1.5 and 4.5 months. There were non-significant trial arm x time effects on rumination (brooding).

For the per protocol sample, trial arm × time effects on mindfulness, worry and rumination (brooding) were all non-significant. Trial arm x time effects were significant in the per protocol sample for self-compassion, between-group effects were non-significant at 1.5 months, although effects at 4.5 months were significant.

Work-related outcomes: Burnout, Compassion-for-Others, and sickness absence There were non-significant differences between trial arms in the rate of change over time on the three Maslach Burnout Inventory subscales (Emotional Exhaustion, Depersonalisation and Personal Accomplishment) in both the ITT and per protocol samples.

In the ITT sample, there were small reductions over time in both arms in compassion-for-others, with a smaller reduction in the Headspace than in Moodzone arm (*b* = 0.02). Trial arm x time effects were significant in the per protocol sample, however between-group effects at 1.5 and 4.5 months were non-significant.

The sickness absence data were highly skewed with a large number of zeros (i.e., no sickness absence). The shape of the distribution was the same after the initial intervention period and post-intervention and in the two arms. The excessive numbers of zeros in the outcome required a different model. Two-part or hurdle models have been proposed where there are large numbers of zeros [4,5]. We initially adapted the model from Magnusson and colleagues [4] which is a marginalized two-part model comprised of a logistic model for whether a report will be zero or not zero, and a model of the number of absences marginalized over the zero and non-zero values. The two models were linked through correlated random effects. However, there were many convergence problems with the models, and it was not possible to interpret them. A simpler version of the model was then used removing the time component so that absenteeism was only examined at T3. This simpler model takes this form in which participants (*j*) were nested within roles (*k*):

$$\begin{matrix} \text{logit}(\pi_{jk}) & =\gamma_{00k}+\gamma_{01}\text{Trial arm}_{jk}+\zeta_{0k}+\epsilon_{0jk} \text{[zeros]} \\ \text{log}(\nu_{jk}) & =\gamma_{10k}+\gamma_{11}\text{Trial arm}_{jk}+\zeta_{1k}+\epsilon_{1jk} \text{[absences]} \end{matrix}$$

This is a Bayesian analysis and *P*-values are not reported. Instead, we have a 95% HPD interval, which is an interval containing the population parameter with 95% probability. In the ITT sample, findings were that the effect of trial arm in predicting absenteeism was close to zero, $\gamma=0.09$ [$-0.18$, $0.34$]. Importantly the 95% HPD interval contains zero suggesting that zero is a plausible effect size, zero was also fairly central in the interval suggesting that trial arm is similarly likely to predict greater absenteeism and lower absenteeism. In other words, use of Headspace does not affect absenteeism in a meaningful way in comparison to Moodzone. The effect of trial arm in predicting zero absenteeism was also very small, $\gamma=-0.12$ [$-0.34$, $0.10$] and the 95% HPD interval contained zero suggesting that zero is a plausible effect size. As such, both parts of the model suggest that Headspace did not affect absenteeism in a meaningful way in comparison to Moodzone. This pattern of findings was replicated in the per protocol sample.

## Intervention Acceptability and Satisfaction

Based on unimputed data, at both T2 and T3, Headspace participants (*M* = 5.07, *SD* = 2.40 and *M* = 5.23, *SD* = 2.52) gave significantly higher ratings than Moodzone participants (*M* = 3.22, *SD* = 2.13 and *M* = 2.93, *SD* = 2.18) in terms of how much they thought their allocated intervention had “really helped their wellbeing”, *t* (1310.67) = -14.82, *P* < .001, *d* = 0.82 and *t* (1046.87) = -15.96, *P* < .001, *d* = 0.98, respectively. Headspace participants at T2 and T3 (*M* = 3.95, *SD* = 1.01 and *M* = 4.00, *SD* = 1.03) were also significantly more likely than Moodzone participants (*M* = 2.84, *SD* = 1.09 and *M* = 2.67, *SD* = 1.18) to say that they would recommend their allocated intervention to friends and family, *t* [1298.95] = -19.01, *P* < .001, *d* = 1.05 and *t* (1028.76) = -19.44, *P* < .001, *d* = 1.19, respectively. Headspace participants at T2 and T3 (*M* = 6.27, *SD* = 2.58 and *M* = 5.96, *SD* = 2.69) were significantly more likely than Moodzone participants (*M* = 3.91, *SD* = 2.64 and *M* = 3.34, *SD* = 2.61) to expect to continue using their intervention over the following six-months, *t* (1322) =-16.47, *P* < .001, *d* = 0.91 *t* (1059) = -16.06, *P* <.001, *d* = 0.99 respectively.

## Use of intervention in other trial arm

At Time 3, significantly more Moodzone participants (*n* = 96) than Headspace participants (*n* = 5) reported having used the non-allocated intervention during the study period, *X^2^* (1) = 94.63, *P* <.001.

## Prior mindfulness experience

There were no significant differences between the number of Headspace (*n* = 63) and Moodzone (*n* = 59) participants who had attended four or more sessions of MBCT or MBSR prior to the study (*X^2^* [1] = 0.17, *P* = .90) and no significant differences in the number of Headspace (*n* = 118) and Moodzone (*n* = 109) participants who had taken part in an MBSH intervention prior to the study, *X^2^* (1) = 0.75, *P* = .79. There were also no significant differences in the number of Headspace (*n* = 153) and Moodzone (*n* = 134) participants who had used Headspace prior to the study (*X^2^* [1] = 0.78, *P* = .38), and no differences between Headspace (*M Rank* = 528.92 ) and Moodzone (*M Rank* = 533.18) participants in the frequency of mindfulness meditation practice undertaken prior to taking part in the study, *U* (*N*_Headspace_ = 543, *N*_Moodzone_ = 518) = 139505.50, *z* = -.234, *P* = .82

## Serious Adverse events and lasting negative effects

No serious adverse events were reported to the study team. Supplementary Material 9 shows the number of participants in each arm slightly or strongly agreeing that they had experienced lasting negative effects of their allocated intervention. After removing participants who appear to have misunderstood the question (as they only reported positive lasting effects in T3 questions), one of the seven Headspace and two of the 13 Moodzone participants showed T1-T3 reliable deterioration of at least nine points on the DASS-21 Stress subscale.

# Additional Discussion

## Mechanisms of action

In terms of proposed mechanisms of action of MBIs [8], there were significant small effects over time between groups on mindfulness, self-compassion and worry, but not on rumination. Improvements in self-compassion over the initial intervention period mediated baseline to post-intervention improvements in stress between trial arms. This shows that Headspace had a beneficial effect on stress outcomes at least in part through improving self-compassion. However, improvements in mindfulness, worry and rumination during the initial intervention period were all found not to mediate the relationship between trial arm and baseline to post-intervention stress outcomes. This is unexpected as Headspace is designed to improve mindfulness; and worry and rumination are key mechanisms of action of MBCT and MBSR [8]. It could be that Moodzone also has beneficial effects on mindfulness, worry and rumination. This is in keeping with findings from a recent meta-analysis of in-person MBIs that found RCTs of MBIs compared to active-control conditions showed only small effects on mindfulness outcomes, and no effects on mindfulness outcomes when compared to CBT-based interventions [9]. This could be a measurement problem, or it could be because mindfulness and non-mindfulness based mental health and wellbeing interventions target similar mechanisms of action [9,10]. If this is the case, it is interesting to note that self-compassion may be a specific mechanism of action in MBIs that may differentiate them from other non-MBI interventions, and this possibility requires further exploration.

For participants engaging in practice at least three times a week (T1-T2), mediation analyses also found that formal engagement (at and above 3 days per week) mediated baseline to T3 improvements in stress between trial arms. This means that the greater improvements in stress for per protocol participants who received Headspace as compared to Moodzone can be explained by per protocol Headspace participants formally engaging more with their allocated intervention than per protocol Moodzone participants. This is indicative of a dose-response relationship between engagement with mindfulness practice and outcome, a relationship that requires exploring in future research.

## Secondary outcomes

Mental Health and Wellbeing Outcomes Headspace led to significant improvements with small effect sizes in depression, anxiety and wellbeing compared to Moodzone over the entire study period and specifically at initial intervention (*g* = 0.16, 0.14, 0.07, respectively) and post-intervention (*g* = 0.20, 0.22, 0.19, respectively). These findings largely align with observations from a meta-analysis, including small but significant between-groups post-intervention effects on depression (*g* = 0.29) and wellbeing (*g* = 0.31) when unsupported mindfulness and acceptance-based self-help interventions were compared to control conditions in a range of populations [11]. However, when only examining studies comparing unguided MBSH with active control conditions in non-clinical populations (mirroring the design of the current study), a recent meta-analysis showed non-significant effects on depression (*g* = 0.05), anxiety (*g* = 0.09, in favour of control conditions) and wellbeing/quality of life (*g* = 0.33) [12], unpublished data, June 2021). This suggests that Headspace may be a good option amongst unguided MBSH resources available when aiming to address mental health outcomes in non-clinical populations, although direct head-to-head comparisons are needed.

In relation to in-person MBIs, Spinelli and colleagues’ [13] meta-analysis identified a medium post-intervention effect on depression (*g* = 0.62) and small effects on anxiety (*g* = 0.39) and wellbeing (*g* = 0.25) when MBSR was compared to active and inactive control conditions among healthcare staff and trainees. Likewise, the in-person MBCT-L course has produced a medium effect on depression (*d* = 0.55), a small effect on anxiety (*d* = 0.33), and a large effect on wellbeing (*d* = 0.92) when compared to wait-list [14]. As with stress outcomes, this suggests that unguided MBSH may produce smaller effects on depression, anxiety, and wellbeing outcomes than in-person, teacher-led MBIs, although a head-to-head comparison is needed to test this possibility directly.

Per protocol analyses on mental health and wellbeing outcomes demonstrated non-significant differences in improvements over time between trial arms. Considering that Headspace participants reported significantly more formal engagement days than Moodzone participants, these findings again raise questions about if and how much of the intention-to-treat effects were driven by Headspace mindfulness content specifically, rather than greater engagement (with a wellbeing intervention) more generally.

Work-Related Outcomes In the present study, both groups showed reductions in compassion-for-others over time. However, Headspace participants showed smaller reductions in compassion-for-others over the study period compared to Moodzone, in both intention-to-treat and per protocol analyses, although between-groups effects after the initial intervention period and at post-intervention were not found. These findings therefore provide preliminary evidence that unguided MBSH may be protective against deterioration in compassion-for-others, however, findings are difficult to interpret given that both groups showed some deterioration and reasons for this are not understood. As such, this finding requires replication and further exploration.

No differences were found in changes over time between trial arms on measures of Burnout (Emotional Exhaustion, Depersonalisation and Personal Accomplishment) or sickness absence, suggesting that a MBSH program was not more effective than an active control at targeting these work-related outcomes. The non-significant effect on sickness absence aligns with findings from a recent RCT with police staff, where both Headspace and an alternative MBSH intervention demonstrated non-significant effects on sickness absence at post-intervention, compared to an inactive control condition [15]. As such, while Headspace can improve stress and mental health-related outcomes associated with sickness absence, this does not translate into a significant reduction in sickness absence days. However, while poor mental health has been shown to be the most common cause of long-term sickness absence [16], and a substantial source of sickness absence within the NHS workforce [17], short-term sickness absence is most commonly attributed to minor physical illnesses [18]. Within our study, those currently on sick leave were not eligible to participate. Moreover, the reported number of sickness-related absences in the three months prior to the start and end of the study period averaged just two days. In retrospect, it is therefore unlikely that Headspace would be able to elicit effects on sickness absences (or more specifically, their causes), which may help to explain the non-significant findings.

In respect of burnout, Spinelli and colleagues’ [13] meta-analysis found that MBSR demonstrated non-significant effects at post-intervention on burnout. A more recent systematic review by Klein and colleagues [19] identified just four RCTs of MBSR compared to inactive control conditions on healthcare workers’ burnout and observed mixed and sometimes contradictory findings; with studies demonstrating significant between-groups post-intervention effects on some, but not all dimensions of burnout. Also, the Strauss et al [14] RCT of MBCT-L found non-significant effects on all three burnout dimensions. As such, the finding that Headspace demonstrated non-significant effects on burnout compared to an active-control condition is not unexpected.

The non-significant effects of Headspace on these work-related outcomes suggest that alternative approaches are needed that are specifically designed to target burnout and sickness absence. Identifying effective strategies is especially important considering that sickness absence is estimated to cost the NHS over one billion pounds per year [20] and recent studies have identified a high prevalence of burnout among healthcare workers (e.g., [21–23]). A review by West et al [24]found that a range of individual, structural and organisational level interventions can have positive effects on burnout in doctors, suggesting that any one solution (e.g., MBIs) may be unrealistically limited in scope to address the systemic problem of burnout in the healthcare workplace.

Deterioration, Serious Adverse Responses and Lasting Negative Effects No serious adverse effects were reported. Overall, 11 out of 272 (4.04%) Headspace and 8 out of 274 (2.92%) of Moodzone participants who provided data at T2 and/or T3 showed reliable deterioration in stress over the course of the study. Whilst we would hope that no participants would show deterioration, although not directly comparable with a help-seeking psychological therapy population, these figures are in line with deterioration found in psychological interventions more broadly of 5.2% [25] and in digital interventions for mental health [26], which may be intervention effects, the result of non-intervention related life events, or both. A small minority of participants reported lasting negative effects from using Headspace (*n* = 7, once seemingly incorrect responses were removed). Reasons given for lasting negative effects of Headspace included a preference to talk to someone in person and frustration at not being able to find the time or space to engage with the intervention due to family and work commitments. Although a small minority, these comments highlight that any one intervention is unlikely to be the solution to healthcare worker stress.

References

1. Jacobson NS, Truax P. Clinical significance: A statistical approach to defining meaningful change in psychotherapy research. J Consult Clin Psychol 1991;59(1). [doi: 10.1037/0022-006X.59.1.12]

2. Crane C, Crane RS, Eames C, Fennell MJV, Silverton S, Williams JMG, Barnhofer T. The effects of amount of home meditation practice in Mindfulness Based Cognitive Therapy on hazard of relapse to depression in the Staying Well after Depression Trial. Behaviour Research and Therapy 2014 Dec;63. [doi: 10.1016/j.brat.2014.08.015]

3. Kazdin AE. Mediators and mechanisms of change in psychotherapy research. Annu Rev Clin Psychol. 2007. p. 1–27. PMID:17716046

4. Morey R, Rouder J. BayesFactor: Computation of Bayes Factors for common designs. R package version 0.9. 12-4.2. 2018.

5. Rouder JN, Speckman PL, Sun D, Morey RD, Iverson G. Bayesian t tests for accepting and rejecting the null hypothesis. Psychon Bull Rev 2009 Apr;16(2):225–237. [doi: 10.3758/PBR.16.2.225]

6. Gunel E, Dickey J. Bayes factors for independence in contingency tables. Biometrika 1974;61(3):545–557. [doi: 10.1093/biomet/61.3.545]

7. Jamil T, Ly A, Morey RD, Love J, Marsman M, Wagenmakers E-J. Default “Gunel and Dickey” Bayes factors for contingency tables. Behav Res Methods 2017 Apr 20;49(2):638–652. [doi: 10.3758/s13428-016-0739-8]

8. Gu J, Strauss C, Bond R, Cavanagh K. How do mindfulness-based cognitive therapy and mindfulness-based stress reduction improve mental health and wellbeing? A systematic review and meta-analysis of mediation studies. Clin Psychol Rev. Elsevier Inc.; 2015. p. 1–12. PMID:25689576

9. Baer R, Gu J, Cavanagh K, Strauss C. Differential sensitivity of mindfulness questionnaires to change with treatment: A systematic review and meta-analysis. Psychol Assess 2019 Oct;31(10). [doi: 10.1037/pas0000744]

10. Walsh KM, Saab BJ, Farb NA. Effects of a mindfulness meditation app on subjective well-being: Active randomized controlled trial and experience sampling study. JMIR Ment Health 2019 Jan 8;6(1). [doi: 10.2196/10844]

11. Spijkerman MPJ, Pots WTM, Bohlmeijer ET. Effectiveness of online mindfulness-based interventions in improving mental health: A review and meta-analysis of randomised controlled trials. Clin Psychol Rev. Elsevier Inc.; 2016. p. 102–114. PMID:27111302

12. Taylor H, Strauss C, Cavanagh K. Can a little bit of mindfulness do you good? A systematic review and meta-analyses of unguided mindfulness-based self-help interventions. Clin Psychol Rev 2021 Nov;89:102078. [doi: 10.1016/j.cpr.2021.102078]

13. Spinelli C, Wisener M, Khoury B. Mindfulness training for healthcare professionals and trainees: A meta-analysis of randomized controlled trials. J Psychosom Res. Elsevier Inc.; 2019. p. 29–38. PMID:30929705

14. Strauss C, Gu J, Montero-Marin J, Whittington A, Chapman C, Kuyken W. Reducing stress and promoting well-being in healthcare workers using mindfulness-based cognitive therapy for life. International Journal of Clinical and Health Psychology Elsevier Doyma; 2021 May 1;21(2). [doi: 10.1016/j.ijchp.2021.100227]

15. Fitzhugh H, Michaelides G, Daniels K, Connolly S. Mindfulness in policing Problem-solving View project Teleworking View project [Internet]. Available from: https://www.researchgate.net/publication/337414836

16. Stewart C. Causes of long-term absences from work in the United Kingdom in 2019. Statista.com. 2020.

17. The King’s Fund. NHS sickness absence: Let’s talk about mental health. 2019 Oct 1 [cited 2020 Aug 28]; Available from: https://www.kingsfund.org.uk/blog/2019/10/nhs-sickness-absence

18. Stewart C. Causes of short-term absences from work in the United Kingdom in 2019. Statista.com. 2020.

19. Klein A, Taieb O, Xavier S, Baubet T, Reyre A. The benefits of mindfulness-based interventions on burnout among health professionals: A systematic review. Explore. Elsevier Inc.; 2020. p. 35–43. PMID:31727578

20. Community Practitioner. NHS sickness absence “costs £1.1bn per year.” [cited 2020 Aug 28];2017. Available from: https://www.communitypractitioner.co.uk/news/2017/04/nhs-sickness-absence-costs-%C2%A311bn-year

21. López‐López IM, Gómez‐Urquiza JL, Cañadas GR, de la Fuente EI, Albendín‐García L, Cañadas‐De la Fuente GA. Prevalence of burnout in mental health nurses and related factors: A systematic review and meta‐analysis. Int J Ment Health Nurs 2019 Oct 27;28(5). [doi: 10.1111/inm.12606]

22. Monsalve-Reyes CS, San Luis-Costas C, Gómez-Urquiza JL, Albendín-García L, Aguayo R, Cañadas-De la Fuente GA. Burnout syndrome and its prevalence in primary care nursing: A systematic review and meta-analysis. BMC Fam Pract 2018 Dec 10;19(1). [doi: 10.1186/s12875-018-0748-z]

23. Woo T, Ho R, Tang A, Tam W. Global prevalence of burnout symptoms among nurses: A systematic review and meta-analysis. J Psychiatr Res 2020 Apr;123. [doi: 10.1016/j.jpsychires.2019.12.015]

24. West CP, Dyrbye LN, Erwin PJ, Shanafelt TD. Interventions to prevent and reduce physician burnout: A systematic review and meta-analysis. The Lancet 2016 Nov;388(10057). [doi: 10.1016/S0140-6736(16)31279-X]

25. Crawford MJ, Thana L, Farquharson L, Palmer L, Hancock E, Bassett P, Clarke J, Parry GD. Patient experience of negative effects of psychological treatment: Results of a national survey. British Journal of Psychiatry 2016 Mar 2;208(3). [doi: 10.1192/bjp.bp.114.162628]

26. Rozental A, Magnusson K, Boettcher J, Andersson G, Carlbring P. For better or worse: An individual patient data meta-analysis of deterioration among participants receiving internet-based Cognitive Behavior Therapy. J Consult Clin Psychol 2017 Feb;85(2). [doi: 10.1037/ccp0000158]
