# Supplementary material for: Health Care Workers’ Need for Headspace: Findings From a Multisite Definitive Randomized Controlled Trial of an Unguided Digital Mindfulness-Based Self-help App to Reduce Healthcare Worker Stress
Source: JMIR Mhealth Uhealth. 2022 Aug 25;10(8):e31744. doi: 10.2196/31744 (PMC9459942; doi:10.2196/31744)
Supplement: Multimedia Appendix 5 [file mhealth_v10i8e31744_app5.docx]

# Appendix 5: Bayes factors for assessing randomisation success

| *Table A5.1: Bayes factors comparing outcome measures at baseline across trial arms* | | |
| --- | --- | --- |
| **Measure** | **BF_10_** | **Error** |
| DASS Stress | 0.162 | 0 |
| DASS Depression | 0.074 | 0 |
| DASS Anxiety | 0.117 | 0 |
| SWEMWBS | 0.052 | 0 |
| MBI EE | 0.061 | 0 |
| MBI Depersonalization | 0.036 | 0 |
| MBI PA | 0.036 | 0 |
| FFMQ | 0.036 | 0 |
| SCS | 0.043 | 0 |
| PSWQ | 0.061 | 0 |
| RSS Brooding | 0.308 | 0 |
| CLS | 0.034 | 0 |
| Sickness absence | 0.035 | 0 |

| *Table A5.2: Bayes factors comparing demographic variables across trial arms* | | |
| --- | --- | --- |
| **Measure** | **BF_10_** | **Error** |
| Highest educational achieved | 0.002 | 0 |
| Ethnicity | 0.001 | 0 |
| Gender | 0.041 | 0 |
| Age | 0.038 | 0 |
| Perceived SES | 0.035 | 0 |
| Hours worked | 0.058 | 0 |
| Income | 0.000 | 0 |
| Marital status | 0.071 | 0 |
| Role | 0.000 | 0 |
| Trust type | 0.000 | 0 |
