# Supplementary material for: Health Care Workers’ Need for Headspace: Findings From a Multisite Definitive Randomized Controlled Trial of an Unguided Digital Mindfulness-Based Self-help App to Reduce Healthcare Worker Stress
Source: JMIR Mhealth Uhealth. 2022 Aug 25;10(8):e31744. doi: 10.2196/31744 (PMC9459942; doi:10.2196/31744)
Supplement: Multimedia Appendix 6 [file mhealth_v10i8e31744_app6.docx]

# Appendix 6: Overall trial arm x time effects of intervention on all outcomes for per protocol sample with multiple imputation (Headspace n=452 and Moodzone n=283 on primary outcome)

| Measure | Unstandardised *b* (SE)  (difference between arms per month) | 95% CI | *t* (df)  [*P*] | ^a^Unstandardised *b* for differences between arms at 1.5 months (SE) [*P*] | ^a^Unstandardised *b* for differences between arms at 4.5 months (SE) [*P*] |
| --- | --- | --- | --- | --- | --- |
| DASS-21  Stress | -0.28 (0.13) | -0.54, -0.02 | -2.13 (507.63)  [.03] | 0.66 (0.48) [.17] | 1.50 (0.62) [.02] |
| DASS-21  Anxiety | -0.14 (0.10) | -0.34, 0.06 | -1.42 (846.77)  [.16] | na | na |
| DASS-21  Depression | -0.23  (0.12) | -0.48, 0.01 | -1.89 (759.99)  [.06] | na | na |
| SWEMWBS  Wellbeing | 0.13  (0.07) | -0.02, 0.27 | 1.75  (805.41)  [.08] | na | na |
| Maslach  Emotional Exhaustion | -0.11  (0.17) | -0.45, 0.23 | -0.64  (695.96)  [.53] | na | na |
| Maslach Depersonal  -isation | -0.10  (0.08) ] | -0.25, 0.06 | -1.25  (846.07)  [.21] | na | na |
| Maslach  Personal Accomp  -lishment | 0.19  (0.11) | -0.03, 0.42 | 1.72  (384.16)  [.09] | na | na |
| FFMQ-15  (minus Observe) | 0.16  (0.10) | -0.04, 0.36 | 1.56 (1,091.58)  [.12] | na | na |
| SCS-SF  Self-Compassion | 0.46  (0.13) | 0.20, 0.72 | 3.52 (636.25)  [< .001] | -0.43 (0.62) [.49] | -1.81 (0.75) [.02] |
| CLS  Compassion for Others | 0.03  (0.01) | 0.00, 0.05 | 2.06 (513.94)  [.04] | -0.09 (0.08) [.25] | -0.17 (0.09) [.06] |
| PSWQ  Worry | -0.29  (0.17) | -0.63, 0.05 | -1.66 (730.68)  [.10] | na | na |
| RRS  Rumination (Brooding) | -0.01  (0.05) | -0.11, 0.09 | -0.23 (432.58)  [.82] | na | na |

^a^ Unstandardised effects at 1.5 and 4.5 months only reported in the event of a significant trial arm x time interaction

Note: A negative value for *b* is in favour of Headspace for DASS-21 subscales, RRS Brooding and PSWQ Worry; a positive value for b is in favour of Headspace for SWEMWS, FFMQ-15 (minus Observe), SCS-SF Self-Compassion and CLS Compassion for Others

*CLS = Compassionate Love Scale; DASS-21 = 21-item Depression, Anxiety and Stress Scale; FFMQ15 = 15-item Five Facets of Mindfulness Questionnaire; Maslach = Maslach Burnout Inventory; PSWQ = Penn State Worry Questionnaire; RRS = Ruminative Response Scale; SCS-SF = Self-Compassion Scale Short-Form; SWEMWBS = Short Warwick Edinburgh Mental Well-Being Scale*
