# Supplementary material for: Health Care Workers’ Need for Headspace: Findings From a Multisite Definitive Randomized Controlled Trial of an Unguided Digital Mindfulness-Based Self-help App to Reduce Healthcare Worker Stress
Source: JMIR Mhealth Uhealth. 2022 Aug 25;10(8):e31744. doi: 10.2196/31744 (PMC9459942; doi:10.2196/31744)
Supplement: Multimedia Appendix 7 [file mhealth_v10i8e31744_app7.docx]

# Appendix 7: Reliable change analysis

Table A7.1: Frequencies for reliable change by trial arm and job role from T1-T2 and T1-T3 for participants scoring in at least the mild range on DASS-21 Stress at T1

| Comparison | Trial arm | RC | Total |
| --- | --- | --- | --- |
| T1-T2 | MZ | No reliable change | 266.00 |
| T1-T2 | MZ | Reliable decrease | 71.00 |
| T1-T2 | MZ | Reliable increase | 10.00 |
| T1-T2 | HS | No reliable change | 234.00 |
| T1-T2 | HS | Reliable decrease | 102.00 |
| T1-T2 | HS | Reliable increase | 7.00 |
| T1-T3 | MZ | No reliable change | 200.00 |
| T1-T3 | MZ | Reliable decrease | 66.00 |
| T1-T3 | MZ | Reliable increase | 8.00 |
| T1-T3 | HS | No reliable change | 161.00 |
| T1-T3 | HS | Reliable decrease | 100.00 |
| T1-T3 | HS | Reliable increase | 11.00 |

Table A7.2: Predicting reliable change from trial Arm for T1-T2 for participants scoring in at least the mild range on DASS-21 Stress at T1

| Comparison | Effect | Estimate | $SE$ | 95% CI | $t$ | DF | $P$ | OR | OR 95% CI |
| --- | --- | --- | --- | --- | --- | --- | --- | --- | --- |
| Reliable decrease | (Intercept) | -1.24 | 0.12 | [$-1.48$, $-1.00$] | -10.31 | 313.69 | < .001 | 0.29 | [$0.23$, $0.37$] |
| Reliable decrease | MZ HS | 0.37 | 0.17 | [$0.05$, $0.70$] | 2.25 | 278.95 | .03 | 1.45 | [$1.05$, $2.01$] |
| Reliable increase | (Intercept) | -3.21 | 0.30 | [$-3.80$, $-2.61$] | -10.60 | 228.03 | < .001 | 0.04 | [$0.02$, $0.07$] |
| Reliable increase | MZ HS | -0.34 | 0.45 | [$-1.22$, $0.55$] | -0.75 | 391.37 | .45 | 0.71 | [$0.29$, $1.73$] |

Table A7.3: Predicting reliable change from trial Arm for T1-T3 for participants scoring in at least the mild range on DASS-21 Stress at T1

| Comparison | Effect | Estimate | $SE$ | 95% CI | $t$ | DF | $P$ | OR | OR 95% CI |
| --- | --- | --- | --- | --- | --- | --- | --- | --- | --- |
| Reliable decrease | (Intercept) | -0.93 | 0.11 | [$-1.16$, $-0.71$] | -8.25 | 280.16 | < .001 | 0.39 | [$0.31$, $0.49$] |
| Reliable decrease | MZ HS | 0.39 | 0.16 | [$0.09$, $0.70$] | 2.52 | 265.95 | .01 | 1.48 | [$1.09$, $2.02$] |
| Reliable increase | (Intercept) | -3.02 | 0.29 | [$-3.59$, $-2.46$] | -10.56 | 240.92 | < .001 | 0.05 | [$0.03$, $0.09$] |
| Reliable increase | MZ HS | 0.23 | 0.42 | [$-0.60$, $1.07$] | 0.55 | 159.35 | .58 | 1.26 | [$0.55$, $2.92$] |
