# Supplementary material for: Health Care Workers’ Need for Headspace: Findings From a Multisite Definitive Randomized Controlled Trial of an Unguided Digital Mindfulness-Based Self-help App to Reduce Healthcare Worker Stress
Source: JMIR Mhealth Uhealth. 2022 Aug 25;10(8):e31744. doi: 10.2196/31744 (PMC9459942; doi:10.2196/31744)
Supplement: Multimedia Appendix 8 [file mhealth_v10i8e31744_app8.docx]

# Appendix 8: Lasting negative effects reported at T3 by study arm with reasons (with some participants giving multiple reasons)

|  | Headspace, *N* after removing participants who appear to have misunderstood the question  (Total N, including participants who seem to have misunderstood the question) | Moodzone, *N* after removing participants who appear to have misunderstood the question  (Total N, including participants who seem to have misunderstood the question) |
| --- | --- | --- |
| Slightly or strongly agreed there were lasting negative effects of the intervention | 7  (9) | 13  (17) |
| Lasting negative effects caused by bringing mindfulness to daily life (Headspace only) | 3  (5) | na |
| Lasting negative effects caused by guided mindfulness practices (Headspace only) | 1  (2) | na |
| Lasting negative effects caused by recommended behavioral tasks (Moodzone only) | na | 4  (5) |
| Lasting negative effects caused by written/audio-visual content | 2  (4) | 4  (5) |
| Reasons given for lasting negative effects | Difficulties accessing the website  Unsure if the correct program had been accessed and finding the verbal guidance distracting and stressful  Frustration and waiting for replies  A preference to speak with someone  No time or space to engage with the intervention due to family and work commitments which left the person feeling resentful of their employer  Feeling beyond help. | Too simple and a waste of time  Hard to use and unhelpful  Stress and frustration at trying to use the website  Issues with links  Needed something different/ things they were already aware of but unable to implement  Being forced to address matters that could have been avoided |
| Reasons given suggesting no lasting negative effects (i.e. the initial question may have not been answered correctly as only positive lasting effects were mentioned in follow-up questions) | Two participants stated in their own words that there were no lasting negative effects and one participant said they were able to share their feelings more easily | Two participants stated in their own words that there were no lasting negative effects, one said the intervention was good and one said the intervention made them somewhat kinder to themselves and their feelings. |
