# Supplementary material for: Health Care Workers’ Need for Headspace: Findings From a Multisite Definitive Randomized Controlled Trial of an Unguided Digital Mindfulness-Based Self-help App to Reduce Healthcare Worker Stress
Source: JMIR Mhealth Uhealth. 2022 Aug 25;10(8):e31744. doi: 10.2196/31744 (PMC9459942; doi:10.2196/31744)
Supplement: Multimedia Appendix 9 [file mhealth_v10i8e31744_app9.docx]

# Appendix 9: Self-reported formal and informal engagement with allocated intervention by trial arm and time

|  |  | Moodzone  M (sd) | Headspace  M (sd) |
| --- | --- | --- | --- |
| Formal engagement (days per week) in past month (T2) and in the past three months (T3) | T2 | 2.33 (2.01) | 3.56 (2.26) |
|  | T3 | 1.35 (1.65) | 2.16 (1.91) |
| Formal engagement (minutes per day on engagement days) in past month (T2) and in the past three months (T3) | T2 | 18.17 (40.09) | 11.51 (20.54) |
|  | T3 | 11.24 (13.68) | 9.89 (8.15) |
| Informal engagement (applying intervention skills in daily life, days per week) in past month (T2) and in the past three months (T3) | T2 | 2.20 (2.08) | 2.92 (2.22) |
|  | T3 | 1.40 (1.77) | 3.00 (2.18) |
