# Supplementary material for: Health Care Workers’ Need for Headspace: Findings From a Multisite Definitive Randomized Controlled Trial of an Unguided Digital Mindfulness-Based Self-help App to Reduce Healthcare Worker Stress
Source: JMIR Mhealth Uhealth. 2022 Aug 25;10(8):e31744. doi: 10.2196/31744 (PMC9459942; doi:10.2196/31744)
Supplement: Multimedia Appendix 10 [file mhealth_v10i8e31744_app10.pdf]

CONSORT-EHEALTH (V 1.6.1) - Submission/Publication Form

The CONSORT-EHEALTH checklist is intended for authors of randomized trials evaluating web-based and Internet-based applications/interventions, including mobile interventions, electronic games (incl multiplayer games), social media, certain telehealth applications, and other interactive and/or networked electronic applications. Some of the items (e.g. all subitems under item 5 - description of the intervention) may also be applicable for other study designs.

The goal of the CONSORT EHEALTH checklist and guideline is to be
a) a guide for reporting for authors of RCTs,
b) to form a basis for appraisal of an ehealth trial (in terms of validity)

CONSORT-EHEALTH items/subitems are MANDATORY reporting items for studies published in the Journal of Medical Internet Research and other journals / scientific societies endorsing the checklist.

Items numbered 1., 2., 3., 4a., 4b etc are original CONSORT or CONSORT-NPT (non-pharmacologic treatment) items.
Items with Roman numerals (i., ii, iii, iv etc.) are CONSORT-EHEALTH extensions/clarifications.

As the CONSORT-EHEALTH checklist is still considered in a formative stage, we would ask that you also RATE ON A SCALE OF 1-5 how important/useful you feel each item is FOR THE PURPOSE OF THE CHECKLIST and reporting guideline (optional).

Mandatory reporting items are marked with a red *.
In the textboxes, either copy & paste the relevant sections from your manuscript into this form - please include any quotes from your manuscript in QUOTATION MARKS,
or answer directly by providing additional information not in the manuscript, or elaborating on why the item was not relevant for this study.

YOUR ANSWERS WILL BE PUBLISHED AS A SUPPLEMENTARY FILE TO YOUR PUBLICATION IN JMIR AND ARE CONSIDERED PART OF YOUR PUBLICATION (IF ACCEPTED).
Please fill in these questions diligently. Information will not be copyedited, so please use proper spelling and grammar, use correct capitalization, and avoid abbreviations.

DO NOT FORGET TO SAVE AS PDF _AND_ CLICK THE SUBMIT BUTTON SO YOUR ANSWERS ARE IN OUR DATABASE !!!

Citation Suggestion (if you append the pdf as Appendix we suggest to cite this paper in the caption):
Eysenbach G, CONSORT-EHEALTH Group
CONSORT-EHEALTH: Improving and Standardizing Evaluation Reports of Web-based and Mobile Health Interventions
J Med Internet Res 2011;13(4):e126
[illegible]

Your response is too large. Try shortening some answers.

PMID: 22209829

claraystrauss@gmail.com (not shared) Switch accounts

Draft not saved

*Required

Your name *

First Last

Clara Strauss

Primary Affiliation (short), City, Country *

University of Toronto, Toronto, Canada

University of Sussex

Your e-mail address *

abc@gmail.com

c.y.strauss@sussex.ac.uk

Title of your manuscript *

Provide the (draft) title of your manuscript.

Do healthcare workers need a little Headspace? Findings from a multi-site definitive randomised controlled trial of an unguided digital mindfulness-based self-help intervention to reduce healthcare worker stress in comparison to an active control

Your response is too large. Try shortening some answers.

Name of your App/Software/Intervention *

If there is a short and a long/alternate name, write the short name first and add the long name in brackets.

Headspace

Evaluated Version (if any)

e.g. "V1", "Release 2017-03-01", "Version 2.0.27913"

Release Feb 2017 to Mar 2019

Language(s) *

What language is the intervention/app in? If multiple languages are available, separate by comma (e.g. "English, French")

English

URL of your Intervention Website or App

e.g. a direct link to the mobile app on app in appstore (itunes, Google Play), or URL of the website. If the intervention is a DVD or hardware, you can also link to an Amazon page.

www.headspace.com

URL of an image/screenshot (optional)

Your answer

Your response is too large. Try shortening some answers.

Accessibility *

Can an enduser access the intervention presently?

- ○ access is free and open
- ○ access only for special usergroups, not open
- ◉ access is open to everyone, but requires payment/subscription/in-app purchases
- ○ app/intervention no longer accessible
- ○ Other:

Primary Medical Indication/Disease/Condition *

e.g. "Stress", "Diabetes", or define the target group in brackets after the condition, e.g. "Autism (Parents of children with)", "Alzheimers (Informal Caregivers of)"

Stress

Primary Outcomes measured in trial *

comma-separated list of primary outcomes reported in the trial

DASS-21 Stress Subscale

Secondary/other outcomes

Are there any other outcomes the intervention is expected to affect?

Depression, Anxiety, Wellbeing, Mindfulness, Self-Compassion, Compassion for others, Rumination, Worry, Burnout

Your response is too large. Try shortening some answers.

Recommended "Dose" *

What do the instructions for users say on how often the app should be used?

- (selected) Approximately Daily
- Approximately Weekly
- Approximately Monthly
- Approximately Yearly
- "as needed"
- Other:

Approx. Percentage of Users (starters) still using the app as recommended after 3 months *

- (selected) unknown / not evaluated
- 0-10%
- 11-20%
- 21-30%
- 31-40%
- 41-50%
- 51-60%
- 61-70%
- 71%-80%
- 81-90%
- 91-100%
- Other:

Your response is too large. Try shortening some answers.

Overall, was the app/intervention effective? *

- [x] yes: all primary outcomes were significantly better in intervention group vs control
- [ ] partly: SOME primary outcomes were significantly better in intervention group vs control
- [ ] no statistically significant difference between control and intervention
- [ ] potentially harmful: control was significantly better than intervention in one or more outcomes
- [ ] inconclusive: more research is needed
- [ ] Other:

Article Preparation Status/Stage *

At which stage in your article preparation are you currently (at the time you fill in this form)

- [ ] not submitted yet - in early draft status
- [ ] not submitted yet - in late draft status, just before submission
- [ ] submitted to a journal but not reviewed yet
- [x] submitted to a journal and after receiving initial reviewer comments
- [ ] submitted to a journal and accepted, but not published yet
- [ ] published
- [ ] Other:

Your response is too large. Try shortening some answers.

Journal *

If you already know where you will submit this paper (or if it is already submitted), please provide the journal name (if it is not JMIR, provide the journal name under "other")

- ○ not submitted yet / unclear where I will submit this
- ○ Journal of Medical Internet Research (JMIR)
- ◉ JMIR mHealth and UHealth
- ○ JMIR Serious Games
- ○ JMIR Mental Health
- ○ JMIR Public Health
- ○ JMIR Formative Research
- ○ Other JMIR sister journal
- ○ Other:

Is this a full powered effectiveness trial or a pilot/feasibility trial? *

- ○ Pilot/feasibility
- ◉ Fully powered

Manuscript tracking number *

If this is a JMIR submission, please provide the manuscript tracking number under "other" (The ms tracking number can be found in the submission acknowledgement email, or when you login as author in JMIR. If the paper is already published in JMIR, then the ms tracking number is the four-digit number at the end of the DOI, to be found at the bottom of each published article in JMIR)

- ○ no ms number (yet) / not (yet) submitted to / published in JMIR
- ◉ Other: 31744

TITLE AND ABSTRACT

Your response is too large. Try shortening some answers.

1a) TITLE: Identification as a randomized trial in the title

1a) Does your paper address CONSORT item 1a? *

I.e does the title contain the phrase "Randomized Controlled Trial"? (if not, explain the reason under "other")

- (x) yes
- ( ) Other:

1a-i) Identify the mode of delivery in the title

Identify the mode of delivery. Preferably use “web-based” and/or “mobile” and/or “electronic game” in the title. Avoid ambiguous terms like “online”, “virtual”, “interactive”. Use “Internet-based” only if Intervention includes non-web-based Internet components (e.g. email), use “computer-based” or “electronic” only if offline products are used. Use “virtual” only in the context of “virtual reality” (3-D worlds). Use “online” only in the context of “online support groups”. Complement or substitute product names with broader terms for the class of products (such as “mobile” or “smart phone” instead of “iphone”), especially if the application runs on different platforms.

	1	2	3	4	5	
subitem not at all important	( )	( )	( )	( )	(x)	essential

Clear selection

Does your paper address subitem 1a-i? *

Copy and paste relevant sections from manuscript title (include quotes in quotation marks "like this" to indicate direct quotes from your manuscript), or elaborate on this item by providing additional information not in the ms, or briefly explain why the item is not applicable/relevant for your study

"unguided digital mindfulness-based self-help intervention"

Your response is too large. Try shortening some answers.

1a-ii) Non-web-based components or important co-interventions in title

Mention non-web-based components or important co-interventions in title, if any (e.g., “with telephone support”).

	1	2	3	4	5	
subitem not at all important	○	○	○	○	◉	essential

Clear selection

Does your paper address subitem 1a-ii?

Copy and paste relevant sections from manuscript title (include quotes in quotation marks "like this" to indicate direct quotes from your manuscript), or elaborate on this item by providing additional information not in the ms, or briefly explain why the item is not applicable/relevant for your study

The title specifis that the interveniton is "unguided"

1a-iii) Primary condition or target group in the title

Mention primary condition or target group in the title, if any (e.g., “for children with Type I Diabetes”) Example: A Web-based and Mobile Intervention with Telephone Support for Children with Type I Diabetes: Randomized Controlled Trial

	1	2	3	4	5	
subitem not at all important	○	○	○	○	◉	essential

Clear selection

Does your paper address subitem 1a-iii? *

Copy and paste relevant sections from manuscript title (include quotes in quotation marks "like this" to indicate direct quotes from your manuscript), or elaborate on this item by providing additional information not in the ms, or briefly explain why the item is not applicable/relevant for your study

"Do healthcare workers need a little Headspace?" and "intervention to reduce healthcare worker stress"

Your response is too large. Try shortening some answers.

1b) ABSTRACT: Structured summary of trial design, methods, results, and conclusions

NPT extension: Description of experimental treatment, comparator, care providers, centers, and blinding status.

1b-i) Key features/functionalities/components of the intervention and comparator in the METHODS section of the ABSTRACT

Mention key features/functionalities/components of the intervention and comparator in the abstract. If possible, also mention theories and principles used for designing the site. Keep in mind the needs of systematic reviewers and indexers by including important synonyms. (Note: Only report in the abstract what the main paper is reporting. If this information is missing from the main body of text, consider adding it)

	1	2	3	4	5	
subitem not at all important	○	○	○	○	◉	essential

Clear selection

Does your paper address subitem 1b-i? *

Copy and paste relevant sections from the manuscript abstract (include quotes in quotation marks "like this" to indicate direct quotes from your manuscript), or elaborate on this item by providing additional information not in the ms, or briefly explain why the item is not applicable/relevant for your study

"In-person mindfulness-based interventions (MBIs) can reduce healthcare worker stress but are not widely available or accessible for busy healthcare workers." and "We sought to investigate the effectiveness and mechanisms of action of an unguided digital MBSH application (Headspace) in reducing healthcare worker stress" and "allocated 1:1 to Headspace or an active-control (Moodzone)" snd "fully-automated"

Your response is too large. Try shortening some answers.

1b-ii) Level of human involvement in the METHODS section of the ABSTRACT

Clarify the level of human involvement in the abstract, e.g., use phrases like “fully automated” vs. “therapist/nurse/care provider/physician-assisted” (mention number and expertise of providers involved, if any). (Note: Only report in the abstract what the main paper is reporting. If this information is missing from the main body of text, consider adding it)

	1	2	3	4	5	
subitem not at all important	○	○	○	○	◉	essential

Clear selection

Does your paper address subitem 1b-ii?

Copy and paste relevant sections from the manuscript abstract (include quotes in quotation marks "like this" to indicate direct quotes from your manuscript), or elaborate on this item by providing additional information not in the ms, or briefly explain why the item is not applicable/relevant for your study

"an unguided digital MBSH application (Headspace)" and "fully automated"

1b-iii) Open vs. closed, web-based (self-assessment) vs. face-to-face assessments in the METHODS section of the ABSTRACT

Mention how participants were recruited (online vs. offline), e.g., from an open access website or from a clinic or a closed online user group (closed usergroup trial), and clarify if this was a purely web-based trial, or there were face-to-face components (as part of the intervention or for assessment). Clearly say if outcomes were self-assessed through questionnaires (as common in web-based trials). Note: In traditional offline trials, an open trial (open-label trial) is a type of clinical trial in which both the researchers and participants know which treatment is being administered. To avoid confusion, use “blinded” or “unblinded” to indicated the level of blinding instead of “open”, as “open” in web-based trials usually refers to “open access” (i.e. participants can self-enrol). (Note: Only report in the abstract what the main paper is reporting. If this information is missing from the main body of text, consider adding it)

	1	2	3	4	5	
subitem not at all important	○	○	○	○	◉	essential

Clear selection

Your response is too large. Try shortening some answers.

Does your paper address subitem 1b-iii?

Copy and paste relevant sections from the manuscript abstract (include quotes in quotation marks "like this" to indicate direct quotes from your manuscript), or elaborate on this item by providing additional information not in the ms, or briefly explain why the item is not applicable/relevant for your study

"with 2182 National Health Service (NHS) England staff recruited online, allocated 1:1"

1b-iv) RESULTS section in abstract must contain use data

Report number of participants enrolled/assessed in each group, the use/uptake of the intervention (e.g., attrition/adherence metrics, use over time, number of logins etc.), in addition to primary/secondary outcomes. (Note: Only report in the abstract what the main paper is reporting. If this information is missing from the main body of text, consider adding it)

	1	2	3	4	5	
subitem not at all important	○	○	○	○	◉	essential

Clear selection

Does your paper address subitem 1b-iv?

Copy and paste relevant sections from the manuscript abstract (include quotes in quotation marks "like this" to indicate direct quotes from your manuscript), or elaborate on this item by providing additional information not in the ms, or briefly explain why the item is not applicable/relevant for your study

"Headspace (n=1095) or an active-control (Moodzone, n=1087)" and "Per protocol effects of Headspace (n=454) versus Moodzone (n=283)"

1b-v) CONCLUSIONS/DISCUSSION in abstract for negative trials

Conclusions/Discussions in abstract for negative trials: Discuss the primary outcome - if the trial is negative (primary outcome not changed), and the intervention was not used, discuss whether negative results are attributable to lack of uptake and discuss reasons. (Note: Only report in the abstract what the main paper is reporting. If this information is missing from the main body of text, consider adding it)

	1	2	3	4	5	
subitem not at all important	◉	○	○	○	○	essential

Clear selection

Your response is too large. Try shortening some answers.

Does your paper address subitem 1b-v?

Copy and paste relevant sections from the manuscript abstract (include quotes in quotation marks "like this" to indicate direct quotes from your manuscript), or elaborate on this item by providing additional information not in the ms, or briefly explain why the item is not applicable/relevant for your study

this is not relevant to the current study

INTRODUCTION

2a) In INTRODUCTION: Scientific background and explanation of rationale

2a-i) Problem and the type of system/solution

Describe the problem and the type of system/solution that is object of the study: intended as stand-alone intervention vs. incorporated in broader health care program? Intended for a particular patient population? Goals of the intervention, e.g., being more cost-effective to other interventions, replace or complement other solutions? (Note: Details about the intervention are provided in “Methods” under 5)

	1	2	3	4	5	
subitem not at all important	○	○	○	○	◉	essential

Clear selection

Your response is too large. Try shortening some answers.

Does your paper address subitem 2a-i? *

Copy and paste relevant sections from the manuscript (include quotes in quotation marks "like this" to indicate direct quotes from your manuscript), or elaborate on this item by providing additional information not in the ms, or briefly explain why the item is not applicable/relevant for your study

Your response is too large. Try shortening some answers.

"Even before the COVID-19 pandemic, findings from meta-analyses demonstrated high prevalence of stress in healthcare workers worldwide [2–4]. Stress is a vulnerability factor for work-related burnout [5], anxiety and depression [6]; all of which are disproportionately prevalent among healthcare workers [7–9] and stress also increases the risk of a number of long-term physical health conditions [10–12]. In the National Health Service (NHS) in England, which employs over 1.2 million healthcare staff [13], 44% of staff report feeling unwell due to work-related stress [13]; a figure which has steadily risen since 2016. Almost a quarter of days lost to staff sickness in the NHS are due to stress, anxiety, depression, or other mental health problems [14] and similar concerns have been noted in healthcare systems internationally [15]. Moreover, stress amongst healthcare workers can compromise patient outcomes and safety [16]. The COVID-19 pandemic is further exacerbating stress and distress for healthcare workers [17,18] and there is therefore an urgent need to find effective, accessible, and affordable ways to reduce healthcare worker stress."

and

"Benefits of MBIs extend beyond clinical populations, with RCTs demonstrating beneficial effects on stress in non-clinical populations [27], including working adults [28] and specifically healthcare workers [29–31]. There are a number of barriers however to healthcare workers attending in-person MBIs including: lack of availability [32]; high workplace demands [33,34] making it difficult for healthcare workers to find the time to attend; stigma-related concerns regarding negative social judgements and disclosure/confidentiality that are more common among healthcare workers compared to those working in other settings [35] Fortunately, mindfulness-based self-help (MBSH) has the potential to increase opportunities for engagement with MBIs with a plethora of MBSH books, online courses and smartphone apps available."

and

"The present study sought to overcome some of the methodological limitations of previous related research and extend our understanding of the potential effects of unguided MBSH among healthcare workers. The aim of this large multi-site randomised controlled trial (RCT) is to explore the effectiveness of unguided digital MBSH in comparison to an active-control condition (note that comparisons to active controls are lacking in RCTs of MBIs; [30]) for healthcare workers in targeting stress (primary outcome), mental health outcomes (depression, anxiety, and wellbeing), work-related outcomes (work-related burnout, sickness absence and compassion-for-others) and proposed mechanisms of action (intervention engagement, rumination, worry, mindfulness and self-compassion). To explore its potential as a healthcare-wide intervention to reduce healthcare worker stress, the trial recruited across the full range of NHS organisation types (GP/primary care, hospital trusts, community trusts, mental health/learning disability trusts and ambulance trusts), across geographically and socio-demographically diverse regions of England and across a range of NHS job roles (medical, nursing, allied health professions, psychological and the wider healthcare support roles)."

Your response is too large. Try shortening some answers.

2a-ii) Scientific background, rationale: What is known about the (type of) system

Scientific background, rationale: What is known about the (type of) system that is the object of the study (be sure to discuss the use of similar systems for other conditions/diagnoses, if appropiate), motivation for the study, i.e. what are the reasons for and what is the context for this specific study, from which stakeholder viewpoint is the study performed, potential impact of findings [2]. Briefly justify the choice of the comparator.

	1	2	3	4	5	
subitem not at all important	○	○	○	○	●	essential

Clear selection

Does your paper address subitem 2a-ii? *

Copy and paste relevant sections from the manuscript (include quotes in quotation marks "like this" to indicate direct quotes from your manuscript), or elaborate on this item by providing additional information not in the ms, or briefly explain why the item is not applicable/relevant for your study

"meta-analyses of RCTs of MBSH have indicated promising effects on stress and mental health outcomes across a range of populations [36,37] Digital MBSH using smartphone apps have the potential to be particularly accessible as they do not rely on the user having a computer or book to hand to engage with the intervention when needed. Headspace [38] is a smartphone app, with over 70 million users and over 2 million subscribers to date worldwide [39]. There's an emerging empirical literature exploring the effectiveness of MBSH apps, including Headspace [40]. Preliminary findings show potential benefits in non-clinical samples, including healthcare workers, however study sample sizes are too small to draw definitive conclusions regarding this working population. Given the early stage of research in this area and studies with small sample sizes, the potential of unguided digital MBSH as a healthcare-wide solution to reduce healthcare worker stress is yet to be explored in an adequately powered trial. Although MBSH can effectively reduce stress in a range of non-clinical populations, it is possible that the particularly high demands of working in healthcare [33,34] will mean that when offered at scale healthcare staff may struggle to engage with the intervention leading to disappointing outcomes. The learning available from a definitive trial of unguided digital MBSH is particularly important at present in the context of rising healthcare worker stress during the COVID-19 pandemic."

2b) In INTRODUCTION: Specific objectives or hypotheses

Your response is too large. Try shortening some answers.

Does your paper address CONSORT subitem 2b? *

Copy and paste relevant sections from the manuscript (include quotes in quotation marks "like this" to indicate direct quotes from your manuscript), or elaborate on this item by providing additional information not in the ms, or briefly explain why the item is not applicable/relevant for your study

"The present study sought to overcome some of the methodological limitations of previous related research and extend our understanding of the potential effects of unguided MBSH among healthcare workers. The aim of this large multi-site randomised controlled trial (RCT) is to explore the effectiveness of unguided digital MBSH in comparison to an active-control condition (note that comparisons to active controls are lacking in RCTs of MBIs; [30]) for healthcare workers in targeting stress (primary outcome), mental health outcomes (depression, anxiety, and wellbeing), work-related outcomes (work-related burnout, sickness absence and compassion-for-others) and proposed mechanisms of action (intervention engagement, rumination, worry, mindfulness and self-compassion). To explore its potential as a healthcare-wide intervention to reduce healthcare worker stress, the trial recruited across the full range of NHS organisation types (GP/primary care, hospital trusts, community trusts, mental health/learning disability trusts and ambulance trusts), across geographically and socio-demographically diverse regions of England and across a range of NHS job roles (medical, nursing, allied health professions, psychological and the wider healthcare support roles)."

and

"The primary hypothesis is that participants allocated to unguided digital MBSH will show greater reductions in stress from baseline to post-intervention (4.5 months following randomisation) in comparison to participants in the active control trial arm. Secondary hypotheses are that unguided digital MBSH will be more effective than the active-control at improving mental health outcomes, work-related outcomes and potential mechanisms of action from baseline to after the initial intervention period (1.5 months post randomisation) and from baseline to post-intervention Analyses examining whether intervention engagement and improvements in mindfulness, self-compassion, worry and rumination mediate the effects of intervention on improvements in stress were planned to ascertain intervention-specific mechanisms of action."

METHODS

3a) Description of trial design (such as parallel, factorial) including allocation ratio

Your response is too large. Try shortening some answers.

Does your paper address CONSORT subitem 3a? *

Copy and paste relevant sections from the manuscript (include quotes in quotation marks "like this" to indicate direct quotes from your manuscript), or elaborate on this item by providing additional information not in the ms, or briefly explain why the item is not applicable/relevant for your study

"This study was a two-arm superiority definitive randomised controlled trial (RCT) with 1:1 allocation"

3b) Important changes to methods after trial commencement (such as eligibility criteria), with reasons

Does your paper address CONSORT subitem 3b? *

Copy and paste relevant sections from the manuscript (include quotes in quotation marks "like this" to indicate direct quotes from your manuscript), or elaborate on this item by providing additional information not in the ms, or briefly explain why the item is not applicable/relevant for your study

There were no important changes to the methods after trial commencement

3b-i) Bug fixes, Downtimes, Content Changes

Bug fixes, Downtimes, Content Changes: ehealth systems are often dynamic systems. A description of changes to methods therefore also includes important changes made on the intervention or comparator during the trial (e.g., major bug fixes or changes in the functionality or content) (5-iii) and other “unexpected events” that may have influenced study design such as staff changes, system failures/downtimes, etc. [2].

	1	2	3	4	5	
subitem not at all important	○	○	○	○	◉	essential

Clear selection

Your response is too large. Try shortening some answers.

Does your paper address subitem 3b-i?

Copy and paste relevant sections from the manuscript (include quotes in quotation marks "like this" to indicate direct quotes from your manuscript), or elaborate on this item by providing additional information not in the ms, or briefly explain why the item is not applicable/relevant for your study

"As Headspace is a 'live' product, the programme structure was non-static and participants were able to access newly released and/or changing content as it became available."

and

"As with Headspace, a 'live' non-static version of Moodzone was utilised in the study, meaning that participants could access new and/or changing content as it became available"

4a) Eligibility criteria for participants

Does your paper address CONSORT subitem 4a? *

Copy and paste relevant sections from the manuscript (include quotes in quotation marks "like this" to indicate direct quotes from your manuscript), or elaborate on this item by providing additional information not in the ms, or briefly explain why the item is not applicable/relevant for your study

"Participants had to: i) be employed within an NHS trust or GP practice in England; ii) be working in roles that involved direct contact with patients for a minimum of one-day per week; iii) be currently in work (i.e. not on long-term sickness absence); iv) be willing to refrain from engaging in other psychological interventions during the course of the study; v) have regular personal access to an Apple/Android smartphone/tablet or a computer with internet access; vi) be aged 18 years or over; and vii) have sufficient English language skills to read and understand the intervention materials. There were no additional exclusion criteria."

Your response is too large. Try shortening some answers.

4a-i) Computer / Internet literacy

Computer / Internet literacy is often an implicit “de facto” eligibility criterion - this should be explicitly clarified.

	1	2	3	4	5	
subitem not at all important	○	○	○	○	◉	essential

Clear selection

Does your paper address subitem 4a-i?

Copy and paste relevant sections from the manuscript (include quotes in quotation marks "like this" to indicate direct quotes from your manuscript), or elaborate on this item by providing additional information not in the ms, or briefly explain why the item is not applicable/relevant for your study

participants should "have regular personal access to an Apple/Android smartphone/tablet or a computer with internet access"

4a-ii) Open vs. closed, web-based vs. face-to-face assessments:

Open vs. closed, web-based vs. face-to-face assessments: Mention how participants were recruited (online vs. offline), e.g., from an open access website or from a clinic, and clarify if this was a purely web-based trial, or there were face-to-face components (as part of the intervention or for assessment), i.e., to what degree got the study team to know the participant. In online-only trials, clarify if participants were quasi-anonymous and whether having multiple identities was possible or whether technical or logistical measures (e.g., cookies, email confirmation, phone calls) were used to detect/prevent these.

	1	2	3	4	5	
subitem not at all important	○	○	○	○	◉	essential

Clear selection

Your response is too large. Try shortening some answers.

Does your paper address subitem 4a-ii? *

Copy and paste relevant sections from the manuscript (include quotes in quotation marks "like this" to indicate direct quotes from your manuscript), or elaborate on this item by providing additional information not in the ms, or briefly explain why the item is not applicable/relevant for your study

"NHS staff were recruited via posters and leaflets in NHS settings, invitation emails sent through NHS organisations and study advertisements on staff webpages and/or newsletters. Potential participants were directed to the study website hosted by Qualtrics XM [57] where they could read the participant information and confirm eligibility and informed consent. After consenting, participants were emailed a web-link along with a unique ID code and asked to complete the T1 measures on Qualtrics. Participants completed T1 measures and were allocated automatically to Headspace or Moodzone, using 1:1 block randomisation with a block size of 4 by Qualtrics. To ensure allocation concealment, members of the research team responsible for collecting data and communicating with participants were blind to block size."

and

"As all assessments were completed online without researchers present, the potential for researcher bias to influence assessment outcomes was minimised."

4a-iii) Information giving during recruitment

Information given during recruitment. Specify how participants were briefed for recruitment and in the informed consent procedures (e.g., publish the informed consent documentation as appendix, see also item X26), as this information may have an effect on user self-selection, user expectation and may also bias results.

	1	2	3	4	5	
subitem not at all important	○	○	○	○	◉	essential

Clear selection

Does your paper address subitem 4a-iii?

Copy and paste relevant sections from the manuscript (include quotes in quotation marks "like this" to indicate direct quotes from your manuscript), or elaborate on this item by providing additional information not in the ms, or briefly explain why the item is not applicable/relevant for your study

"where they could read the participant information and confirm eligibility and informed consent (see Appendix 2)."

Your response is too large. Try shortening some answers.

4b) Settings and locations where the data were collected

Does your paper address CONSORT subitem 4b? *

Copy and paste relevant sections from the manuscript (include quotes in quotation marks "like this" to indicate direct quotes from your manuscript), or elaborate on this item by providing additional information not in the ms, or briefly explain why the item is not applicable/relevant for your study

"As all assessments were completed online without
researchers present, the potential for researcher bias to influence assessment outcomes was minimised."

4b-i) Report if outcomes were (self-)assessed through online questionnaires

Clearly report if outcomes were (self-)assessed through online questionnaires (as common in web-based trials) or otherwise.

	1	2	3	4	5	
subitem not at all important	○	○	○	○	●	essential

Clear selection

Does your paper address subitem 4b-i? *

Copy and paste relevant sections from the manuscript (include quotes in quotation marks "like this" to indicate direct quotes from your manuscript), or elaborate on this item by providing additional information not in the ms, or briefly explain why the item is not applicable/relevant for your study

"participants were emailed a web-link along with a unique ID code and asked to self-complete the T1 measures on Qualtrics" and "At 35-days post-randomisation participants were emailed a link to complete T2 assessments on Qualtrics" and "At 125-days post-randomisation, participants were emailed a link to complete the T3 assessment on Qualtrics"

Your response is too large. Try shortening some answers.

4b-ii) Report how institutional affiliations are displayed

Report how institutional affiliations are displayed to potential participants [on ehealth media], as affiliations with prestigious hospitals or universities may affect volunteer rates, use, and reactions with regards to an intervention.(Not a required item – describe only if this may bias results)

	1	2	3	4	5	
subitem not at all important	○	○	○	○	◉	essential

Clear selection

Does your paper address subitem 4b-ii?

Copy and paste relevant sections from the manuscript (include quotes in quotation marks "like this" to indicate direct quotes from your manuscript), or elaborate on this item by providing additional information not in the ms, or briefly explain why the item is not applicable/relevant for your study

The participant information sheet informed participants that the study was sponsored by the University of Sussex.

5) The interventions for each group with sufficient details to allow replication, including how and when they were actually administered

5-i) Mention names, credential, affiliations of the developers, sponsors, and owners

Mention names, credential, affiliations of the developers, sponsors, and owners [6] (if authors/evaluators are owners or developer of the software, this needs to be declared in a “Conflict of interest” section or mentioned elsewhere in the manuscript).

	1	2	3	4	5	
subitem not at all important	○	○	○	○	◉	essential

Clear selection

Your response is too large. Try shortening some answers.

Does your paper address subitem 5-i?

Copy and paste relevant sections from the manuscript (include quotes in quotation marks "like this" to indicate direct quotes from your manuscript), or elaborate on this item by providing additional information not in the ms, or briefly explain why the item is not applicable/relevant for your study

Your response is too large. Try shortening some answers.

"Headspace

The Headspace MBSH digital programme [38] offers a range of brief mindfulness-based practices alongside psychoeducational material. The Headspace MBSH digital programme can be accessed via a website [43] or app available on the Apple app store or the Android Play store. Headspace offers a range of mindfulness-based practices and psychoeducational animations, including introductory series that consist of daily sessions designed to teach foundational mindfulness principles and practices, as well as packs designed for more specific emotional difficulties (e.g. stress, anxiety) and brief 'SOS' mindfulness practices, designed to be used in times of acute stress. Headspace also offers guidance on informal mindfulness practices that can be undertaken while carrying out everyday activities, such as running and cycling and there is written information, including research evidence relating to mindfulness and an FAQ section. At the time of the study mindfulness practices were verbally guided by Andy Puddicombe; a founder of Headspace with many years' experience of mindfulness practice. For the introductory sessions, users are verbally guided to bring non-judgemental awareness to the body, breath, thoughts and feelings, with later sessions also inviting users to bring awareness to difficulties arising during practice (e.g. boredom, restlessness) and behavioural choices. At the time of recruitment, users were invited to start the Headspace programme by completing the 'Take Ten' introductory pack, which involved undertaking guided ten-minute mindfulness practices daily for ten consecutive days. On completion of 'Take Ten', participants were provided with unlimited access to the full range of Headspace content. While participants were free to choose which content they engaged with, they were invited to carry out at least one ten-minute mindfulness practice daily for the duration of the study. While practices range in length from three to 20-minutes, users can select the duration of most sessions. As Headspace is a 'live' product, the programme structure was non-static and participants were able to access newly released and/or changing content as it became available.

Moodzone

The NHS Moodzone psychoeducational digital platform [41] was utilised as an active control. At the time of recruitment, the website offered a range of evidence-based psychosocial recommendations, advice and guidance on how to effectively manage work-related stress and mental health difficulties. The initial webpage was divided into the following sections: 'What causes work stress?', 'How to manage work stress', 'Learn to speak out', 'Spot the signs of work stress', and 'Who else can help with work stress?'; each providing information and/ or recommendations or guidance relevant to the respective question. Moodzone also included information, videos and audio-tracks/podcasts and links to other related resources. Participants were invited to engage with the Moodzone website for 10-minutes a day for the duration of the study. It should be noted that while very similar content is still available [44] , the Moodzone website utilised in the study is no longer active. As with Headspace, a 'live' non-static version of Moodzone was utilised in the study, meaning that participants could access new and/or changing content as it became available. Prior to the present study, adequately powered trials of Moodzone had not been undertaken. However, related evidence from a meta-analysis of RCTs identified a significant small effect (d = .20, Pp = .04) of passive psychoeducational interventions compared to control conditions in reducing depression and psychological distress at post-intervention [45]."

Your response is too large. Try shortening some answers.

5-ii) Describe the history/development process

Describe the history/development process of the application and previous formative evaluations (e.g., focus groups, usability testing), as these will have an impact on adoption/use rates and help with interpreting results.

	1	2	3	4	5	
subitem not at all important	◉	○	○	○	○	essential

Clear selection

Does your paper address subitem 5-ii?

Copy and paste relevant sections from the manuscript (include quotes in quotation marks "like this" to indicate direct quotes from your manuscript), or elaborate on this item by providing additional information not in the ms, or briefly explain why the item is not applicable/relevant for your study

Both interventions were developed prior to the study and independently of the research team.

5-iii) Revisions and updating

Revisions and updating. Clearly mention the date and/or version number of the application/intervention (and comparator, if applicable) evaluated, or describe whether the intervention underwent major changes during the evaluation process, or whether the development and/or content was “frozen” during the trial. Describe dynamic components such as news feeds or changing content which may have an impact on the replicability of the intervention (for unexpected events see item 3b).

	1	2	3	4	5	
subitem not at all important	○	○	○	○	◉	essential

Clear selection

Your response is too large. Try shortening some answers.

Does your paper address subitem 5-iii?

Copy and paste relevant sections from the manuscript (include quotes in quotation marks "like this" to indicate direct quotes from your manuscript), or elaborate on this item by providing additional information not in the ms, or briefly explain why the item is not applicable/relevant for your study

"As Headspace is a 'live' product, the programme structure was non-static and participants were able to access newly released and/or changing content as it became available."

"As with Headspace, a 'live' non-static version of Moodzone was utilised in the study, meaning that participants could access new and/or changing content as it became available."

"It should be noted that while very similar content is still available [44] , the Moodzone website utilised in the study is no longer active."

5-iv) Quality assurance methods

Provide information on quality assurance methods to ensure accuracy and quality of information provided [1], if applicable.

	1	2	3	4	5	
subitem not at all important	(●)	( )	( )	( )	( )	essential

Clear selection

Does your paper address subitem 5-iv?

Copy and paste relevant sections from the manuscript (include quotes in quotation marks "like this" to indicate direct quotes from your manuscript), or elaborate on this item by providing additional information not in the ms, or briefly explain why the item is not applicable/relevant for your study

This is not relevant to the current study

Your response is too large. Try shortening some answers.

5-v) Ensure replicability by publishing the source code, and/or providing screenshots/screen-capture video, and/or providing flowcharts of the algorithms used

Ensure replicability by publishing the source code, and/or providing screenshots/screen-capture video, and/or providing flowcharts of the algorithms used. Replicability (i.e., other researchers should in principle be able to replicate the study) is a hallmark of scientific reporting.

	1	2	3	4	5	
subitem not at all important	◉	○	○	○	○	essential

Clear selection

Does your paper address subitem 5-v?

Copy and paste relevant sections from the manuscript (include quotes in quotation marks "like this" to indicate direct quotes from your manuscript), or elaborate on this item by providing additional information not in the ms, or briefly explain why the item is not applicable/relevant for your study

Neither of the interventions were owned by the research team and no members of the research team had access to the intervention source code.

5-vi) Digital preservation

Digital preservation: Provide the URL of the application, but as the intervention is likely to change or disappear over the course of the years; also make sure the intervention is archived (Internet Archive, webcitation.org, and/or publishing the source code or screenshots/videos alongside the article). As pages behind login screens cannot be archived, consider creating demo pages which are accessible without login.

	1	2	3	4	5	
subitem not at all important	○	○	○	○	◉	essential

Clear selection

Your response is too large. Try shortening some answers.

Does your paper address subitem 5-vi?

Copy and paste relevant sections from the manuscript (include quotes in quotation marks "like this" to indicate direct quotes from your manuscript), or elaborate on this item by providing additional information not in the ms, or briefly explain why the item is not applicable/relevant for your study

Historical versions of Headspace can be access from Headspace at www.headspace.com.

5-vii) Access

Access: Describe how participants accessed the application, in what setting/context, if they had to pay (or were paid) or not, whether they had to be a member of specific group. If known, describe how participants obtained “access to the platform and Internet” [1]. To ensure access for editors/reviewers/readers, consider to provide a “backdoor” login account or demo mode for reviewers/readers to explore the application (also important for archiving purposes, see vi).

	1	2	3	4	5	
subitem not at all important	○	○	○	○	◉	essential

Clear selection

Does your paper address subitem 5-vii? *

Copy and paste relevant sections from the manuscript (include quotes in quotation marks "like this" to indicate direct quotes from your manuscript), or elaborate on this item by providing additional information not in the ms, or briefly explain why the item is not applicable/relevant for your study

""Following completion of the T1 assessment, participants were emailed information on how to access their allocated intervention." and "Intervention participants were given 12 months free access to Headspace and Moodzone was available free-of-charge."

Your response is too large. Try shortening some answers.

5-viii) Mode of delivery, features/functionalities/components of the intervention and comparator, and the theoretical framework

Describe mode of delivery, features/functionalities/components of the intervention and comparator, and the theoretical framework [6] used to design them (instructional strategy [1], behaviour change techniques, persuasive features, etc., see e.g., [7, 8] for terminology). This includes an in-depth description of the content (including where it is coming from and who developed it) [1]," whether [and how] it is tailored to individual circumstances and allows users to track their progress and receive feedback" [6]. This also includes a description of communication delivery channels and – if computer-mediated communication is a component – whether communication was synchronous or asynchronous [6]. It also includes information on presentation strategies [1], including page design principles, average amount of text on pages, presence of hyperlinks to other resources, etc. [1].

	1	2	3	4	5	
subitem not at all important	○	○	○	○	◉	essential

Clear selection

Your response is too large. Try shortening some answers.

Does your paper address subitem 5-viii? *

Copy and paste relevant sections from the manuscript (include quotes in quotation marks "like this" to indicate direct quotes from your manuscript), or elaborate on this item by providing additional information not in the ms, or briefly explain why the item is not applicable/relevant for your study

Your response is too large. Try shortening some answers.

"Headspace

The Headspace MBSH digital programme [38] offers a range of brief mindfulness-based practices alongside psychoeducational material. The Headspace MBSH digital programme can be accessed via a website [43] or app available on the Apple app store or the Android Play store. Headspace offers a range of mindfulness-based practices and psychoeducational animations, including introductory series that consist of daily sessions designed to teach foundational mindfulness principles and practices, as well as packs designed for more specific emotional difficulties (e.g. stress, anxiety) and brief 'SOS' mindfulness practices, designed to be used in times of acute stress. Headspace also offers guidance on informal mindfulness practices that can be undertaken while carrying out everyday activities, such as running and cycling and there is written information, including research evidence relating to mindfulness and an FAQ section. At the time of the study mindfulness practices were verbally guided by Andy Puddicombe; a founder of Headspace with many years' experience of mindfulness practice. For the introductory sessions, users are verbally guided to bring non-judgemental awareness to the body, breath, thoughts and feelings, with later sessions also inviting users to bring awareness to difficulties arising during practice (e.g. boredom, restlessness) and behavioural choices. At the time of recruitment, users were invited to start the Headspace programme by completing the 'Take Ten' introductory pack, which involved undertaking guided ten-minute mindfulness practices daily for ten consecutive days. On completion of 'Take Ten', participants were provided with unlimited access to the full range of Headspace content. While participants were free to choose which content they engaged with, they were invited to carry out at least one ten-minute mindfulness practice daily for the duration of the study. While practices range in length from three to 20-minutes, users can select the duration of most sessions. As Headspace is a 'live' product, the programme structure was non-static and participants were able to access newly released and/or changing content as it became available.

Moodzone

The NHS Moodzone psychoeducational digital platform [41] was utilised as an active control. At the time of recruitment, the website offered a range of evidence-based psychosocial recommendations, advice and guidance on how to effectively manage work-related stress and mental health difficulties. The initial webpage was divided into the following sections: 'What causes work stress?', 'How to manage work stress', 'Learn to speak out', 'Spot the signs of work stress', and 'Who else can help with work stress?'; each providing information and/ or recommendations or guidance relevant to the respective question. Moodzone also included information, videos and audio-tracks/podcasts and links to other related resources. Participants were invited to engage with the Moodzone website for 10-minutes a day for the duration of the study. It should be noted that while very similar content is still available [44] , the Moodzone website utilised in the study is no longer active. As with Headspace, a 'live' non-static version of Moodzone was utilised in the study, meaning that participants could access new and/or changing content as it became available. Prior to the present study, adequately powered trials of Moodzone had not been undertaken. However, related evidence from a meta-analysis of RCTs identified a significant small effect ($d = .20$, $Pp = .04$) of passive psychoeducational interventions compared to control conditions in reducing depression and psychological distress at post-intervention [45]."

Your response is too large. Try shortening some answers.

5-ix) Describe use parameters

Describe use parameters (e.g., intended “doses” and optimal timing for use). Clarify what instructions or recommendations were given to the user, e.g., regarding timing, frequency, heaviness of use, if any, or was the intervention used ad libitum.

	1	2	3	4	5	
subitem not at all important	○	○	○	○	◉	essential

Clear selection

Does your paper address subitem 5-ix?

Copy and paste relevant sections from the manuscript (include quotes in quotation marks "like this" to indicate direct quotes from your manuscript), or elaborate on this item by providing additional information not in the ms, or briefly explain why the item is not applicable/relevant for your study

"participants were invited to engage with their allocated intervention for ten-minutes per day, every day, for the initial 30-day study period. At 35-days post-randomisation participants were emailed a link to complete T2 assessment on Qualtrics and invited to continue engaging with their allocated intervention for ten-minutes per day for the remaining 90-day study period."

5-x) Clarify the level of human involvement

Clarify the level of human involvement (care providers or health professionals, also technical assistance) in the e-intervention or as co-intervention (detail number and expertise of professionals involved, if any, as well as “type of assistance offered, the timing and frequency of the support, how it is initiated, and the medium by which the assistance is delivered”. It may be necessary to distinguish between the level of human involvement required for the trial, and the level of human involvement required for a routine application outside of a RCT setting (discuss under item 21 – generalizability).

	1	2	3	4	5	
subitem not at all important	○	○	○	○	◉	essential

Clear selection

Your response is too large. Try shortening some answers.

Does your paper address subitem 5-x?

Copy and paste relevant sections from the manuscript (include quotes in quotation marks "like this" to indicate direct quotes from your manuscript), or elaborate on this item by providing additional information not in the ms, or briefly explain why the item is not applicable/relevant for your study

"The research team were available to answer technical questions/ queries via email. No further support was provided."

5-xi) Report any prompts/reminders used

Report any prompts/reminders used: Clarify if there were prompts (letters, emails, phone calls, SMS) to use the application, what triggered them, frequency etc. It may be necessary to distinguish between the level of prompts/reminders required for the trial, and the level of prompts/reminders for a routine application outside of a RCT setting (discuss under item 21 – generalizability).

	1	2	3	4	5	
subitem not at all important	○	○	○	○	◉	essential

Clear selection

Does your paper address subitem 5-xi? *

Copy and paste relevant sections from the manuscript (include quotes in quotation marks "like this" to indicate direct quotes from your manuscript), or elaborate on this item by providing additional information not in the ms, or briefly explain why the item is not applicable/relevant for your study

For the intervneiton: "The research team were available to answer technical questions/ queries via email. No further support was provided."

For study assessments: "Participants who did not complete assessments within one week of them being sent were reminded to do so via email. One reminder email was sent to complete T1 assessments and a maximum of four reminder emails at weekly intervals were sent for T2 and T3 assessments."

Your response is too large. Try shortening some answers.

5-xii) Describe any co-interventions (incl. training/support)

Describe any co-interventions (incl. training/support): Clearly state any interventions that are provided in addition to the targeted eHealth intervention, as ehealth intervention may not be designed as stand-alone intervention. This includes training sessions and support [1]. It may be necessary to distinguish between the level of training required for the trial, and the level of training for a routine application outside of a RCT setting (discuss under item 21 – generalizability.

	1	2	3	4	5	
subitem not at all important	◉	○	○	○	○	essential

Clear selection

Does your paper address subitem 5-xii? *

Copy and paste relevant sections from the manuscript (include quotes in quotation marks "like this" to indicate direct quotes from your manuscript), or elaborate on this item by providing additional information not in the ms, or briefly explain why the item is not applicable/relevant for your study

This item is not relevant to the current study

6a) Completely defined pre-specified primary and secondary outcome measures, including how and when they were assessed

Your response is too large. Try shortening some answers.

Does your paper address CONSORT subitem 6a? *

Copy and paste relevant sections from the manuscript (include quotes in quotation marks "like this" to indicate direct quotes from your manuscript), or elaborate on this item by providing additional information not in the ms, or briefly explain why the item is not applicable/relevant for your study

Your response is too large. Try shortening some answers.

"Participants completed the following measures at Time 1 (T1), Time 2 (T2) and Time 3 (T3) unless stated otherwise.
• Short version of the Depression, Anxiety and Stress Scales (DASS-21; [46]). The Stress subscale was the primary outcome with T3 being the primary end point.
• Short Warwick Edinburgh Mental Wellbeing Scale (SWEMWBS; [47])
• Maslach Burnout Inventory [5]
• 15-item version (minus 'observe') of the Five Facets of Mindfulness Questionnaire (FFMQ-15; [48])
• Self-Compassion Scale-Short-Form (SCS-SF; [49]
• Compassionate Love Scale (CLS; [50])
• Penn State Worry Questionnaire (PSWQ, [51]
• Brooding subscale of the Ruminative Response Scale (RRS Brooding; [52])
• Sickness absence measured at T1 and T3 was assessed using one-item that asked participants to report how many days they had been absent from work due to sickness during the previous three months.
• Demographic information assessed at T1 including participants' age, gender, marital status, number of children under 18 years, number of children aged 18 years or over, NHS job role, trust and team, number of hours worked per week in said NHS job role, highest level of education, individual and household annual incomes, ethnicity, and perceived relative socio-economic status (SES), with response options from 1 (lowest) to 10 (highest) perceived SES [53].
• Intervention expectancy at T1 (Credibility/Expectancy Questionnaire, CEQ; [54])
• Self-reported intervention engagement at T2 and T3: (1) formal engagement: self-reported average number of days/week spent following a guided mindfulness meditation on Headspace/following a recommended stress-management/wellbeing strategy on the Moodzone webpage; and (2) informal engagement: self-reported average number of days/week they brought mindfulness to a daily activity or brought the recommended stress-management/wellbeing strategies from Moodzone into their daily life. At T2, these questions were asked in relation to the previous month and at T3 they were asked in relation to the previous three months.
• Intervention evaluations at T2 and T3: Participants were asked how likely they were to recommend the intervention to friends and family; how much they really felt that their allocated intervention had helped their wellbeing; and how likely they were to continue practicing mindfulness (Headspace participants) or stress management/wellbeing strategies (Moodzone participants) over the following six-months.
• Hypothesis guess at T3: Participants were asked to state what they thought the purpose of the study was.
• Intervention deviations at T3: Participants were asked to indicate whether or not they had engaged with the alternative study intervention during the course of the study.
• Prior mindfulness experience at T3: Participants were asked to indicate their experiences of mindfulness prior to the study, including MBCT/MBSR, MBSH, Headspace, and how often they had practiced mindfulness.
• Serious adverse events were recorded in line with National Institute for Health Research (NIHR) Good Clinical Practice guidelines [55].
• Participants were also asked to indicate the extent to which they agreed/disagreed that they had experienced "lasting bad effects" from using their allocated intervention (based on Crawford et al [56]). If participants agreed or strongly agreed, they were asked to provide further details."

Your response is too large. Try shortening some answers.

6a-i) Online questionnaires: describe if they were validated for online use and apply CHERRIES items to describe how the questionnaires were designed/deployed

If outcomes were obtained through online questionnaires, describe if they were validated for online use and apply CHERRIES items to describe how the questionnaires were designed/deployed [9].

	1	2	3	4	5	
subitem not at all important	○	○	○	○	◉	essential

Clear selection

Does your paper address subitem 6a-i?

Copy and paste relevant sections from manuscript text

"The Checklist for Reporting Results of Internet E-Surveys (CHERRIES) was adhered to and the majority of measures (including the primary outcome) were validated for online delivery."

6a-ii) Describe whether and how “use” (including intensity of use/dosage) was defined/measured/monitored

Describe whether and how “use” (including intensity of use/dosage) was defined/measured/monitored (logins, logfile analysis, etc.). Use/adoption metrics are important process outcomes that should be reported in any ehealth trial.

	1	2	3	4	5	
subitem not at all important	○	○	○	○	◉	essential

Clear selection

Your response is too large. Try shortening some answers.

Does your paper address subitem 6a-ii?

Copy and paste relevant sections from manuscript text

"Self-reported intervention engagement at T2 and T3: (1) formal engagement: self-reported average number of days/week spent following a guided mindfulness meditation on Headspace/following a recommended stress-management/wellbeing strategy on the Moodzone webpage; and (2) informal engagement: self-reported average number of days/week they brought mindfulness to a daily activity or brought the recommended stress-management/wellbeing strategies from Moodzone into their daily life. At T2, these questions were asked in relation to the previous month and at T3 they were asked in relation to the previous three months."

6a-iii) Describe whether, how, and when qualitative feedback from participants was obtained

Describe whether, how, and when qualitative feedback from participants was obtained (e.g., through emails, feedback forms, interviews, focus groups).

	1	2	3	4	5	
subitem not at all important	○	○	◉	○	○	essential

Clear selection

Does your paper address subitem 6a-iii?

Copy and paste relevant sections from manuscript text

We did not obtain qualitative feedback from participants

6b) Any changes to trial outcomes after the trial commenced, with reasons

Your response is too large. Try shortening some answers.

Does your paper address CONSORT subitem 6b? *

Copy and paste relevant sections from the manuscript (include quotes in quotation marks "like this" to indicate direct quotes from your manuscript), or elaborate on this item by providing additional information not in the ms, or briefly explain why the item is not applicable/relevant for your study

Not changes to trial outcomes were made after the trial commenced

7a) How sample size was determined

NPT: When applicable, details of whether and how the clustering by care provides or centers was addressed

7a-i) Describe whether and how expected attrition was taken into account when calculating the sample size

Describe whether and how expected attrition was taken into account when calculating the sample size.

	1	2	3	4	5	
subitem not at all important	○	○	○	○	◉	essential

Clear selection

Does your paper address subitem 7a-i?

Copy and paste relevant sections from manuscript title (include quotes in quotation marks "like this" to indicate direct quotes from your manuscript), or elaborate on this item by providing additional information not in the ms, or briefly explain why the item is not applicable/relevant for your study

""Sample size calculations were conducted using G*Power [42] and indicated that 527 complete cases per study arm (1054 total) would be needed to detect a small between-groups difference of d = .20 (Pp = .05; 90% power; two-tailed) on the primary outcome (stress at Time 3), with this estimate based on a meta-analysis of MBSH on stress outcomes [37]. A conservative estimate of 50% study drop-out rate was assumed [36], giving a total required sample size of 2108 (n = 1054 per arm)."

7b) When applicable, explanation of any interim analyses and stopping guidelines

Your response is too large. Try shortening some answers.

Does your paper address CONSORT subitem 7b? *

Copy and paste relevant sections from the manuscript (include quotes in quotation marks "like this" to indicate direct quotes from your manuscript), or elaborate on this item by providing additional information not in the ms, or briefly explain why the item is not applicable/relevant for your study

Interim analyses were not conducted.

8a) Method used to generate the random allocation sequence

NPT: When applicable, how care providers were allocated to each trial group

Does your paper address CONSORT subitem 8a? *

Copy and paste relevant sections from the manuscript (include quotes in quotation marks "like this" to indicate direct quotes from your manuscript), or elaborate on this item by providing additional information not in the ms, or briefly explain why the item is not applicable/relevant for your study

"Participants completed T1 measures and were allocated automatically to Headspace or Moodzone, using 1:1 block randomisation with a block size of 4 by Qualtrics. To ensure allocation concealment, members of the research team responsible for collecting data and communicating with participants were blind to block size."

8b) Type of randomisation; details of any restriction (such as blocking and block size)

Does your paper address CONSORT subitem 8b? *

Copy and paste relevant sections from the manuscript (include quotes in quotation marks "like this" to indicate direct quotes from your manuscript), or elaborate on this item by providing additional information not in the ms, or briefly explain why the item is not applicable/relevant for your study

""Participants completed T1 measures and were allocated automatically to Headspace or Moodzone, using 1:1 block randomisation with a block size of 4 by Qualtrics."

Your response is too large. Try shortening some answers.

9) Mechanism used to implement the random allocation sequence (such as sequentially numbered containers), describing any steps taken to conceal the sequence until interventions were assigned

Does your paper address CONSORT subitem 9? *

Copy and paste relevant sections from the manuscript (include quotes in quotation marks "like this" to indicate direct quotes from your manuscript), or elaborate on this item by providing additional information not in the ms, or briefly explain why the item is not applicable/relevant for your study

"Participants completed T1 measures and were allocated automatically to Headspace or Moodzone, using 1:1 block randomisation with a block size of 4 by Qualtrics. To ensure allocation concealment, members of the research team responsible for collecting data and communicating with participants were blind to block size."

10) Who generated the random allocation sequence, who enrolled participants, and who assigned participants to interventions

Does your paper address CONSORT subitem 10? *

Copy and paste relevant sections from the manuscript (include quotes in quotation marks "like this" to indicate direct quotes from your manuscript), or elaborate on this item by providing additional information not in the ms, or briefly explain why the item is not applicable/relevant for your study

"Participants completed T1 measures and were allocated automatically to Headspace or Moodzone, using 1:1 block randomisation with a block size of 4 by Qualtrics. To ensure allocation concealment, members of the research team responsible for collecting data and communicating with participants were blind to block size."

11a) If done, who was blinded after assignment to interventions (for example, participants, care providers, those assessing outcomes) and how

NPT: Whether or not administering co-interventions were blinded to group assignment

Your response is too large. Try shortening some answers.

11a-i) Specify who was blinded, and who wasn't

Specify who was blinded, and who wasn't. Usually, in web-based trials it is not possible to blind the participants [1, 3] (this should be clearly acknowledged), but it may be possible to blind outcome assessors, those doing data analysis or those administering co-interventions (if any).

	1	2	3	4	5	
subitem not at all important	○	○	○	○	◉	essential

Clear selection

Does your paper address subitem 11a-i? *

Copy and paste relevant sections from the manuscript (include quotes in quotation marks "like this" to indicate direct quotes from your manuscript), or elaborate on this item by providing additional information not in the ms, or briefly explain why the item is not applicable/relevant for your study

"To ensure allocation concealment, members of the research team responsible for collecting data and communicating with participants were blind to block size."

and

"To improve trial quality and blind participants to study condition and direction of study hypotheses, advertisements about the study simply referred to both conditions as "online interventions to reduce NHS staff stress" and details of the alternative/non-allocated intervention was not communicated to participants until T3 assessments (after outcome and engagement measures had been taken). As all assessments were completed online without researchers present, the potential for researcher bias to influence assessment outcomes was minimised. All but the mediation analysis was conducted blind to study arm."

11a-ii) Discuss e.g., whether participants knew which intervention was the "intervention of interest" and which one was the "comparator"

Informed consent procedures (4a-ii) can create biases and certain expectations - discuss e.g., whether participants knew which intervention was the "intervention of interest" and which one was the "comparator".

	1	2	3	4	5	
subitem not at all important	○	○	○	○	◉	essential

Clear selection

Your response is too large. Try shortening some answers.

Does your paper address subitem 11a-ii?

Copy and paste relevant sections from the manuscript (include quotes in quotation marks "like this" to indicate direct quotes from your manuscript), or elaborate on this item by providing additional information not in the ms, or briefly explain why the item is not applicable/relevant for your study

"To improve trial quality and blind participants to study condition and direction of study hypotheses, advertisements about the study simply referred to both conditions as “online interventions to reduce NHS staff stress” and details of the alternative/non-allocated intervention was not communicated to participants until T3 assessments (after outcome and engagement measures had been taken)."

11b) If relevant, description of the similarity of interventions

(this item is usually not relevant for ehealth trials as it refers to similarity of a placebo or sham intervention to a active medication/intervention)

Does your paper address CONSORT subitem 11b? *

Copy and paste relevant sections from the manuscript (include quotes in quotation marks "like this" to indicate direct quotes from your manuscript), or elaborate on this item by providing additional information not in the ms, or briefly explain why the item is not applicable/relevant for your study

The active control intervnention was described as follows:

"Moodzone

The NHS Moodzone psychoeducational digital platform [41] was utilised as an active control. At the time of recruitment, the website offered a range of evidence-based psychosocial recommendations, advice and guidance on how to effectively manage work-related stress and mental health difficulties. The initial webpage was divided into the following sections: 'What causes work stress?', 'How to manage work stress', 'Learn to speak out', 'Spot the signs of work stress', and 'Who else can help with work stress?'; each providing information and/ or recommendations or guidance relevant to the respective question. Moodzone also included information, videos and audio-tracks/podcasts and links to other related resources. Participants were invited to engage with the Moodzone website for 10-minutes a day for the duration of the study. It should be noted that while very similar content is still available [44] , the Moodzone website utilised in the study is no longer active. As with Headspace, a ’live’ non-static version of Moodzone was utilised in the study, meaning that participants could access new and/or changing content as it became available. Prior to the present study, adequately powered trials of Moodzone had not been undertaken. However, related evidence from a meta-analysis of RCTs identified a significant small effect (d = .20, Pp = .04) of passive psychoeducational interventions compared to control conditions in reducing depression and psychological distress at post-intervention [45]."

Your response is too large. Try shortening some answers.

12a) Statistical methods used to compare groups for primary and secondary outcomes

NPT: When applicable, details of whether and how the clustering by care providers or centers was addressed

Your response is too large. Try shortening some answers.

Does your paper address CONSORT subitem 12a? *

Copy and paste relevant sections from the manuscript (include quotes in quotation marks "like this" to indicate direct quotes from your manuscript), or elaborate on this item by providing additional information not in the ms, or briefly explain why the item is not applicable/relevant for your study

Your response is too large. Try shortening some answers.

The information from the manuscript is too large for this section so please see the manuscript for full details

"Data Analysis Plan

Handling Missing Data

There were a minimal number of items missing at the item level and missing values for missing items were imputed (using a single imputation) using predictive mean matching in mice [65]. At the scale level, multiple imputation was used to handle missing values. Further details are given in Supplementary Material 2.

Model Selection

Because participants were nested within job roles (Level 3), there are good reasons to model variation in intervention effects between job roles [66]. In such a model there is participant-level randomization to intervention arms and job role acts as a crossed effect. We can think of time (i) as being nested within participants (j), nested within job roles (k), but the effect of treatment arm occurs at level 2 (the participant level), not Level 3 (the job role level), of the hierarchy. This situation is described by the following model:
Level 1:

[see manuscript]

This saturated model includes random effects for time, trial arm and their interaction at Level 3. However, this model resulted in convergence problems that yielded erratic estimates of the random effects involving trial arm in the raw sample and nearly all imputed samples. Based on this pre-analysis, a simpler model seemed more appropriate in which only time was treated as a random effect and only at Level 2. However, to model Level 3 variability in outcomes, a random intercept (at Level 3) was included. This simpler model converged in all imputed samples. The resulting model can be described as follows (notice at level 3 two random effects have been knocked out):
Level 1:

[see manuscript]

To sum up, hypotheses were tested using a growth model fit as a general linear mixed model (GLMM) with observations (Level 1) nested within participants (Level 2) nested within job roles (Level 3). Time (time from baseline that responses were recorded) and trial arm are predictors. The effect of the intervention was quantified and tested with the interaction between time and trial arm, which shows the degree to which the change in the outcome over time is different in the two trial arms. Between-group effects were reported separately at T2 and T3 in the event of significant (Pp < .05) trial arm x time interactions.
The primary analysis was conducted on the ITT sample with the multiply imputed data sets. Secondary analysis was conducted on the per protocol sample (formal engagement T1-T2 on at least 3 days/week; [67]) with the multiply imputed data sets."

Your response is too large. Try shortening some answers.

12a-i) Imputation techniques to deal with attrition / missing values

Imputation techniques to deal with attrition / missing values: Not all participants will use the intervention/comparator as intended and attrition is typically high in ehealth trials. Specify how participants who did not use the application or dropped out from the trial were treated in the statistical analysis (a complete case analysis is strongly discouraged, and simple imputation techniques such as LOCF may also be problematic [4]).

	1	2	3	4	5	
subitem not at all important	○	○	○	○	●	essential

Clear selection

Does your paper address subitem 12a-i? *

Copy and paste relevant sections from the manuscript (include quotes in quotation marks "like this" to indicate direct quotes from your manuscript), or elaborate on this item by providing additional information not in the ms, or briefly explain why the item is not applicable/relevant for your study

"Handling Missing Data

There were a minimal number of items missing at the item level and missing values for missing items were imputed (using a single imputation) using predictive mean matching in mice [65]. At the scale level, multiple imputation was used to handle missing values. Further details are given in Supplementary Material 2."

12b) Methods for additional analyses, such as subgroup analyses and adjusted analyses

Does your paper address CONSORT subitem 12b? *

Copy and paste relevant sections from the manuscript (include quotes in quotation marks "like this" to indicate direct quotes from your manuscript), or elaborate on this item by providing additional information not in the ms, or briefly explain why the item is not applicable/relevant for your study

Subgroup analyses were not conducted

Your response is too large. Try shortening some answers.

X26) REB/IRB Approval and Ethical Considerations [recommended as subheading under "Methods"] (not a CONSORT item)

X26-i) Comment on ethics committee approval

	1	2	3	4	5	
subitem not at all important	○	○	○	○	◉	essential

Clear selection

Does your paper address subitem X26-i?

Copy and paste relevant sections from the manuscript (include quotes in quotation marks "like this" to indicate direct quotes from your manuscript), or elaborate on this item by providing additional information not in the ms, or briefly explain why the item is not applicable/relevant for your study

""Ethical approval (Reference: ER/HT207/8) was provided by the University of Sussex and study approval was granted by the Health Research Authority (Reference: 16/HRA/5525)."

x26-ii) Outline informed consent procedures

Outline informed consent procedures e.g., if consent was obtained offline or online (how? Checkbox, etc.?), and what information was provided (see 4a-ii). See [6] for some items to be included in informed consent documents.

	1	2	3	4	5	
subitem not at all important	○	○	○	○	◉	essential

Clear selection

Your response is too large. Try shortening some answers.

Does your paper address subitem X26-ii?

Copy and paste relevant sections from the manuscript (include quotes in quotation marks "like this" to indicate direct quotes from your manuscript), or elaborate on this item by providing additional information not in the ms, or briefly explain why the item is not applicable/relevant for your study

"Potential participants were directed to the study website hosted by Qualtrics XM [57] where they could read the participant information and confirm eligibility and informed consent. "

X26-iii) Safety and security procedures

Safety and security procedures, incl. privacy considerations, and any steps taken to reduce the likelihood or detection of harm (e.g., education and training, availability of a hotline)

	1	2	3	4	5	
subitem not at all important	○	○	○	○	◉	essential

Clear selection

Does your paper address subitem X26-iii?

Copy and paste relevant sections from the manuscript (include quotes in quotation marks "like this" to indicate direct quotes from your manuscript), or elaborate on this item by providing additional information not in the ms, or briefly explain why the item is not applicable/relevant for your study

Details of who participants can contact if distress are shown in the Participant Information Sheet (Appendix 2).

RESULTS

13a) For each group, the numbers of participants who were randomly assigned, received intended treatment, and were analysed for the primary outcome

NPT: The number of care providers or centers performing the intervention in each group and the number of patients treated by each care provider in each center

Your response is too large. Try shortening some answers.

Does your paper address CONSORT subitem 13a? *

Copy and paste relevant sections from the manuscript (include quotes in quotation marks "like this" to indicate direct quotes from your manuscript), or elaborate on this item by providing additional information not in the ms, or briefly explain why the item is not applicable/relevant for your study

This information is shown in the CONSORT diagram.

13b) For each group, losses and exclusions after randomisation, together with reasons

Does your paper address CONSORT subitem 13b? (NOTE: Preferably, this is shown in a CONSORT flow diagram) *

Copy and paste relevant sections from the manuscript (include quotes in quotation marks "like this" to indicate direct quotes from your manuscript), or elaborate on this item by providing additional information not in the ms, or briefly explain why the item is not applicable/relevant for your study

This is shown in the CONSORT diagram

13b-i) Attrition diagram

Strongly recommended: An attrition diagram (e.g., proportion of participants still logging in or using the intervention/comparator in each group plotted over time, similar to a survival curve) or other figures or tables demonstrating usage/dose/engagement.

	1	2	3	4	5	
subitem not at all important	○	○	○	○	○	essential

Does your paper address subitem 13b-i?

Copy and paste relevant sections from the manuscript or cite the figure number if applicable (include quotes in quotation marks "like this" to indicate direct quotes from your manuscript), or elaborate on this item by providing additional information not in the ms, or briefly explain why the item is not applicable/relevant for your study

This is not included in the current study.

Your response is too large. Try shortening some answers.

14a) Dates defining the periods of recruitment and follow-up

Does your paper address CONSORT subitem 14a? *

Copy and paste relevant sections from the manuscript (include quotes in quotation marks "like this" to indicate direct quotes from your manuscript), or elaborate on this item by providing additional information not in the ms, or briefly explain why the item is not applicable/relevant for your study

"Recruitment took place between 21st February 2017 and 18th September 2018." and "At 125-days post-randomisation, participants were emailed a link to complete the T3 assessment on Qualtrics, with T3 completed on average at 4.5 months post-randomisation."

14a-i) Indicate if critical "secular events" fell into the study period

Indicate if critical "secular events" fell into the study period, e.g., significant changes in Internet resources available or "changes in computer hardware or Internet delivery resources"

	1	2	3	4	5	
subitem not at all important	○	○	○	○	◉	essential

Clear selection

Does your paper address subitem 14a-i?

Copy and paste relevant sections from the manuscript (include quotes in quotation marks "like this" to indicate direct quotes from your manuscript), or elaborate on this item by providing additional information not in the ms, or briefly explain why the item is not applicable/relevant for your study

This is not revelant to the study.

14b) Why the trial ended or was stopped (early)

Your response is too large. Try shortening some answers.

Does your paper address CONSORT subitem 14b? *

Copy and paste relevant sections from the manuscript (include quotes in quotation marks "like this" to indicate direct quotes from your manuscript), or elaborate on this item by providing additional information not in the ms, or briefly explain why the item is not applicable/relevant for your study

this is not relevant to the current study

15) A table showing baseline demographic and clinical characteristics for each group

NPT: When applicable, a description of care providers (case volume, qualification, expertise, etc.) and centers (volume) in each group

Does your paper address CONSORT subitem 15? *

Copy and paste relevant sections from the manuscript (include quotes in quotation marks "like this" to indicate direct quotes from your manuscript), or elaborate on this item by providing additional information not in the ms, or briefly explain why the item is not applicable/relevant for your study

This is included in the demograhpics table

15-i) Report demographics associated with digital divide issues

In ehealth trials it is particularly important to report demographics associated with digital divide issues, such as age, education, gender, social-economic status, computer/Internet/ehealth literacy of the participants, if known.

	1	2	3	4	5	
subitem not at all important	○	○	○	○	◉	essential

Clear selection

Does your paper address subitem 15-i? *

Copy and paste relevant sections from the manuscript (include quotes in quotation marks "like this" to indicate direct quotes from your manuscript), or elaborate on this item by providing additional information not in the ms, or briefly explain why the item is not applicable/relevant for your study

This information is included in the demographics table.

Your response is too large. Try shortening some answers.

16) For each group, number of participants (denominator) included in each analysis and whether the analysis was by original assigned groups

16-i) Report multiple "denominators" and provide definitions

Report multiple "denominators" and provide definitions: Report N's (and effect sizes) "across a range of study participation [and use] thresholds" [1], e.g., N exposed, N consented, N used more than x times, N used more than y weeks, N participants "used" the intervention/comparator at specific pre-defined time points of interest (in absolute and relative numbers per group). Always clearly define "use" of the intervention.

	1	2	3	4	5	
subitem not at all important	○	○	○	○	◉	essential

Clear selection

Does your paper address subitem 16-i? *

Copy and paste relevant sections from the manuscript (include quotes in quotation marks "like this" to indicate direct quotes from your manuscript), or elaborate on this item by providing additional information not in the ms, or briefly explain why the item is not applicable/relevant for your study

This information is provided in the demographics table and the main analysis is described as intention to treat.

16-ii) Primary analysis should be intent-to-treat

Primary analysis should be intent-to-treat, secondary analyses could include comparing only "users", with the appropriate caveats that this is no longer a randomized sample (see 18-i).

	1	2	3	4	5	
subitem not at all important	○	○	○	○	◉	essential

Clear selection

Your response is too large. Try shortening some answers.

Does your paper address subitem 16-ii?

Copy and paste relevant sections from the manuscript (include quotes in quotation marks "like this" to indicate direct quotes from your manuscript), or elaborate on this item by providing additional information not in the ms, or briefly explain why the item is not applicable/relevant for your study

The primary analysis is defined as intention to treat :

"Primary Outcome (Stress)

Intention to Treat Analysis Table 2 shows that the main effects of trial arm"

17a) For each primary and secondary outcome, results for each group, and the estimated effect size and its precision (such as 95% confidence interval)

Does your paper address CONSORT subitem 17a? *

Copy and paste relevant sections from the manuscript (include quotes in quotation marks "like this" to indicate direct quotes from your manuscript), or elaborate on this item by providing additional information not in the ms, or briefly explain why the item is not applicable/relevant for your study

This information is shown in the included tables.

17a-i) Presentation of process outcomes such as metrics of use and intensity of use

In addition to primary/secondary (clinical) outcomes, the presentation of process outcomes such as metrics of use and intensity of use (dose, exposure) and their operational definitions is critical. This does not only refer to metrics of attrition (13-b) (often a binary variable), but also to more continuous exposure metrics such as “average session length”. These must be accompanied by a technical description how a metric like a “session” is defined (e.g., timeout after idle time) [1] (report under item 6a).

	1	2	3	4	5	
subitem not at all important	○	○	○	○	◉	essential

Clear selection

Your response is too large. Try shortening some answers.

Does your paper address subitem 17a-i?

Copy and paste relevant sections from the manuscript (include quotes in quotation marks "like this" to indicate direct quotes from your manuscript), or elaborate on this item by providing additional information not in the ms, or briefly explain why the item is not applicable/relevant for your study

Engagement (days/week) with each intervention is reported in the Results section.

17b) For binary outcomes, presentation of both absolute and relative effect sizes is recommended

Does your paper address CONSORT subitem 17b? *

Copy and paste relevant sections from the manuscript (include quotes in quotation marks "like this" to indicate direct quotes from your manuscript), or elaborate on this item by providing additional information not in the ms, or briefly explain why the item is not applicable/relevant for your study

this is not relevant to the current study

18) Results of any other analyses performed, including subgroup analyses and adjusted analyses, distinguishing pre-specified from exploratory

Does your paper address CONSORT subitem 18? *

Copy and paste relevant sections from the manuscript (include quotes in quotation marks "like this" to indicate direct quotes from your manuscript), or elaborate on this item by providing additional information not in the ms, or briefly explain why the item is not applicable/relevant for your study

This is not relevant to the current study

Your response is too large. Try shortening some answers.

18-i) Subgroup analysis of comparing only users

A subgroup analysis of comparing only users is not uncommon in ehealth trials, but if done, it must be stressed that this is a self-selected sample and no longer an unbiased sample from a randomized trial (see 16-iii).

	1	2	3	4	5	
subitem not at all important	○	○	○	○	◉	essential

Clear selection

Does your paper address subitem 18-i?

Copy and paste relevant sections from the manuscript (include quotes in quotation marks "like this" to indicate direct quotes from your manuscript), or elaborate on this item by providing additional information not in the ms, or briefly explain why the item is not applicable/relevant for your study

Per protocol analyses are reported in the Results section and per protocol findings in the Appendix.

19) All important harms or unintended effects in each group

(for specific guidance see CONSORT for harms)

Does your paper address CONSORT subitem 19? *

Copy and paste relevant sections from the manuscript (include quotes in quotation marks "like this" to indicate direct quotes from your manuscript), or elaborate on this item by providing additional information not in the ms, or briefly explain why the item is not applicable/relevant for your study

Negative effects are reported in the Results and in the Appendix

Your response is too large. Try shortening some answers.

19-i) Include privacy breaches, technical problems

Include privacy breaches, technical problems. This does not only include physical “harm” to participants, but also incidents such as perceived or real privacy breaches [1], technical problems, and other unexpected/unintended incidents. “Unintended effects” also includes unintended positive effects [2].

	1	2	3	4	5	
subitem not at all important	○	○	○	○	◉	essential

Clear selection

Does your paper address subitem 19-i?

Copy and paste relevant sections from the manuscript (include quotes in quotation marks "like this" to indicate direct quotes from your manuscript), or elaborate on this item by providing additional information not in the ms, or briefly explain why the item is not applicable/relevant for your study

This is not relevant to the curent study

19-ii) Include qualitative feedback from participants or observations from staff/researchers

Include qualitative feedback from participants or observations from staff/researchers, if available, on strengths and shortcomings of the application, especially if they point to unintended/unexpected effects or uses. This includes (if available) reasons for why people did or did not use the application as intended by the developers.

	1	2	3	4	5	
subitem not at all important	○	○	○	○	◉	essential

Clear selection

Does your paper address subitem 19-ii?

Copy and paste relevant sections from the manuscript (include quotes in quotation marks "like this" to indicate direct quotes from your manuscript), or elaborate on this item by providing additional information not in the ms, or briefly explain why the item is not applicable/relevant for your study

This information was not recorded in the current study.

Your response is too large. Try shortening some answers.

DISCUSSION

22) Interpretation consistent with results, balancing benefits and harms, and considering other relevant evidence

NPT: In addition, take into account the choice of the comparator, lack of or partial blinding, and unequal expertise of care providers or centers in each group

22-i) Restate study questions and summarize the answers suggested by the data, starting with primary outcomes and process outcomes (use)

Restate study questions and summarize the answers suggested by the data, starting with primary outcomes and process outcomes (use).

	1	2	3	4	5	
subitem not at all important	○	○	○	○	◉	essential

Clear selection

Does your paper address subitem 22-i? *

Copy and paste relevant sections from the manuscript (include quotes in quotation marks "like this" to indicate direct quotes from your manuscript), or elaborate on this item by providing additional information not in the ms, or briefly explain why the item is not applicable/relevant for your study

Findings are summarised at the start of the Discussion

22-ii) Highlight unanswered new questions, suggest future research

Highlight unanswered new questions, suggest future research.

	1	2	3	4	5	
subitem not at all important	○	○	○	○	◉	essential

Clear selection

Your response is too large. Try shortening some answers.

Does your paper address subitem 22-ii?

Copy and paste relevant sections from the manuscript (include quotes in quotation marks "like this" to indicate direct quotes from your manuscript), or elaborate on this item by providing additional information not in the ms, or briefly explain why the item is not applicable/relevant for your study

Gaps not addressed in the current study are highlighted in the Discussion.

20) Trial limitations, addressing sources of potential bias, imprecision, and, if relevant, multiplicity of analyses

20-i) Typical limitations in ehealth trials

Typical limitations in ehealth trials: Participants in ehealth trials are rarely blinded. Ehealth trials often look at a multiplicity of outcomes, increasing risk for a Type I error. Discuss biases due to non-use of the intervention/usability issues, biases through informed consent procedures, unexpected events.

	1	2	3	4	5	
subitem not at all important	○	○	○	○	◉	essential

Clear selection

Does your paper address subitem 20-i? *

Copy and paste relevant sections from the manuscript (include quotes in quotation marks "like this" to indicate direct quotes from your manuscript), or elaborate on this item by providing additional information not in the ms, or briefly explain why the item is not applicable/relevant for your study

A range of limitations are specified in the Discussion.

21) Generalisability (external validity, applicability) of the trial findings

NPT: External validity of the trial findings according to the intervention, comparators, patients, and care providers or centers involved in the trial

Your response is too large. Try shortening some answers.

21-i) Generalizability to other populations

Generalizability to other populations: In particular, discuss generalizability to a general Internet population, outside of a RCT setting, and general patient population, including applicability of the study results for other organizations

	1	2	3	4	5	
subitem not at all important	○	○	○	○	◉	essential

Clear selection

Does your paper address subitem 21-i?

Copy and paste relevant sections from the manuscript (include quotes in quotation marks "like this" to indicate direct quotes from your manuscript), or elaborate on this item by providing additional information not in the ms, or briefly explain why the item is not applicable/relevant for your study

Implications of study findings are stated, including the limits to this.

21-ii) Discuss if there were elements in the RCT that would be different in a routine application setting

Discuss if there were elements in the RCT that would be different in a routine application setting (e.g., prompts/reminders, more human involvement, training sessions or other co-interventions) and what impact the omission of these elements could have on use, adoption, or outcomes if the intervention is applied outside of a RCT setting.

	1	2	3	4	5	
subitem not at all important	○	○	○	○	◉	essential

Clear selection

Does your paper address subitem 21-ii?

Copy and paste relevant sections from the manuscript (include quotes in quotation marks "like this" to indicate direct quotes from your manuscript), or elaborate on this item by providing additional information not in the ms, or briefly explain why the item is not applicable/relevant for your study

Routine implementation of Headspace and how this might differ in routine pratice are explored in the Discussion.

Your response is too large. Try shortening some answers.

OTHER INFORMATION

23) Registration number and name of trial registry

Does your paper address CONSORT subitem 23? *

Copy and paste relevant sections from the manuscript (include quotes in quotation marks "like this" to indicate direct quotes from your manuscript), or elaborate on this item by providing additional information not in the ms, or briefly explain why the item is not applicable/relevant for your study

"Trial registration: The study was prospectively registered on the International Standard Randomised Controlled Trial Number (ISCTN) Register [1]"

24) Where the full trial protocol can be accessed, if available

Does your paper address CONSORT subitem 24? *

Cite a Multimedia Appendix, other reference, or copy and paste relevant sections from the manuscript (include quotes in quotation marks "like this" to indicate direct quotes from your manuscript), or elaborate on this item by providing additional information not in the ms, or briefly explain why the item is not applicable/relevant for your study

"Trial registration: The study was prospectively registered on the International Standard Randomised Controlled Trial Number (ISCTN) Register [1]"

25) Sources of funding and other support (such as supply of drugs), role of funders

Your response is too large. Try shortening some answers.

Does your paper address CONSORT subitem 25? *

Copy and paste relevant sections from the manuscript (include quotes in quotation marks "like this" to indicate direct quotes from your manuscript), or elaborate on this item by providing additional information not in the ms, or briefly explain why the item is not applicable/relevant for your study

""Funding: This study was supported by a PhD studentship awarded to the first author jointly funded by Headspace and by the ESRC (ES/J500173/1). Data analysis and writing of the manuscript were conducted independently from Headspace and Headspace did not have any influence over the decision to publish findings."

X27) Conflicts of Interest (not a CONSORT item)

X27-i) State the relation of the study team towards the system being evaluated

In addition to the usual declaration of interests (financial or otherwise), also state the relation of the study team towards the system being evaluated, i.e., state if the authors/evaluators are distinct from or identical with the developers/sponsors of the intervention.

	1	2	3	4	5	
subitem not at all important	○	○	○	○	◉	essential

Clear selection

Does your paper address subitem X27-i?

Copy and paste relevant sections from the manuscript (include quotes in quotation marks "like this" to indicate direct quotes from your manuscript), or elaborate on this item by providing additional information not in the ms, or briefly explain why the item is not applicable/relevant for your study

""Declaration of Interests: The first author was awarded a PhD studentship for this work by Headspace. The second author has received research and consultancy funding from digital healthcare companies including Headspace. The third author declares no conflicts of interest. The last author is Research Lead for [institution name removed to preserve anonymity] and has received research funding, including from NIHR and Headspace, to evaluate mindfulness-based interventions"

Your response is too large. Try shortening some answers.

As a result of using this checklist, did you make changes in your manuscript? *

- ○ yes, major changes
- ◉ yes, minor changes
- ○ no

What were the most important changes you made as a result of using this checklist?

Adding in the participant information sheet and consent form as appendices

How much time did you spend on going through the checklist INCLUDING making changes in your manuscript *

It took about 4 hours to go through the checklist

As a result of using this checklist, do you think your manuscript has improved? *

- ◉ yes
- ○ no
- ○ Other:

Your response is too large. Try shortening some answers.

Would you like to become involved in the CONSORT EHEALTH group?

This would involve for example becoming involved in participating in a workshop and writing an "Explanation and Elaboration" document

- ○ yes
- ◉ no
- ○ Other:

Clear selection

Any other comments or questions on CONSORT EHEALTH

It would be helpful to be able to save reponses when in progress to avoid potentially losing responses (e.g. if network connection drops). Also, the form requires pasting from the manuscript but about half way through a warning came up saying the length was exceeded so that pasting from the manuscript was no longer possible. Otherwise though this was a helpful process.

STOP - Save this form as PDF before you click submit

To generate a record that you filled in this form, we recommend to generate a PDF of this page (on a Mac, simply select "print" and then select "print as PDF") before you submit it.

When you submit your (revised) paper to JMIR, please upload the PDF as supplementary file.

Don't worry if some text in the textboxes is cut off, as we still have the complete information in our database. Thank you!

Final step: Click submit !

Click submit so we have your answers in our database!

Submit Clear form

Never submit passwords through Google Forms.

This content is neither created nor endorsed by Google. Report Abuse - Terms of Service - Privacy Policy

Google Forms

Your response is too large. Try shortening some answers.
